# Fragment-Based Discovery of KLK6 and KLK7 Inhibitors

**DOI:** 10.1021/acs.jcim.6c02059

**Published:** 2026-08-05

**Authors:** Renato Ferreira de Freitas, Feryel Soualmia, Rilès Boumali, Yasmine Harrak, Chahrazade El Amri

**Affiliations:** † Centro de Ciências Naturais e Humanas, 585876 Universidade Federal do ABC, Rua Arcturus 3, São Bernardo do Campo, São Paulo 09606-070, Brazil; ‡ Programa de Pós-Graduação em Ciências Farmacêuticas, Escola de Farmácia, Universidade Federal de Ouro Preto, Ouro Preto, Minas Gerais 35400-000, Brazil; § Sorbonne Université, CNRS, Inserm, Development, 27063 Adaptation and Ageing, Dev2A, F-75005 Paris, France; ∥ Sorbonne Université, CNRS, Inserm, Institut de Biologie Paris-Seine, IBPS, F-75005 Paris, France

## Abstract

Human tissue kallikreins are serine proteases implicated in the pathogenesis of neurodegenerative diseases, skin disorders, and various cancers. Despite their therapeutic relevance, the development of selective small-molecule inhibitors for these enzymes has been limited compared to other serine proteases. To address this, we implemented a fragment-based virtual screening (FBVS) strategy to identify chemically tractable starting points for the inhibition of KLK6 and KLK7. The computational workflow, combining structure-based docking tailored to serine protease active sites, was experimentally validated using *in vitro* enzymatic assays. Notably, the approach yielded hit rates (IC_50_ ≤ 100 μM) ranging from 4% (KLK7) to 21.6% (KLK6), underscoring both the robustness of the screening cascade and the druggability of these targets. Preliminary structure–activity relationship analysis identified fragment **69** as a potent and ligand-efficient KLK6 inhibitor (IC_50_ = 2.1 μM, LE = 0.43). For KLK7, a distinct neutral 2-hydroxyquinoline fragment (**38**) was validated as a ligand-efficient hit (IC_50_ = 43.3 μM; LE = 0.46). Overall, this study demonstrates that kallikrein-related peptidases are amenable to fragment-based discovery and provides chemically simple, highly ligand-efficient fragments that serve as promising starting points for lead optimization targeting kallikrein-driven pathologies.

## Graphical abstract

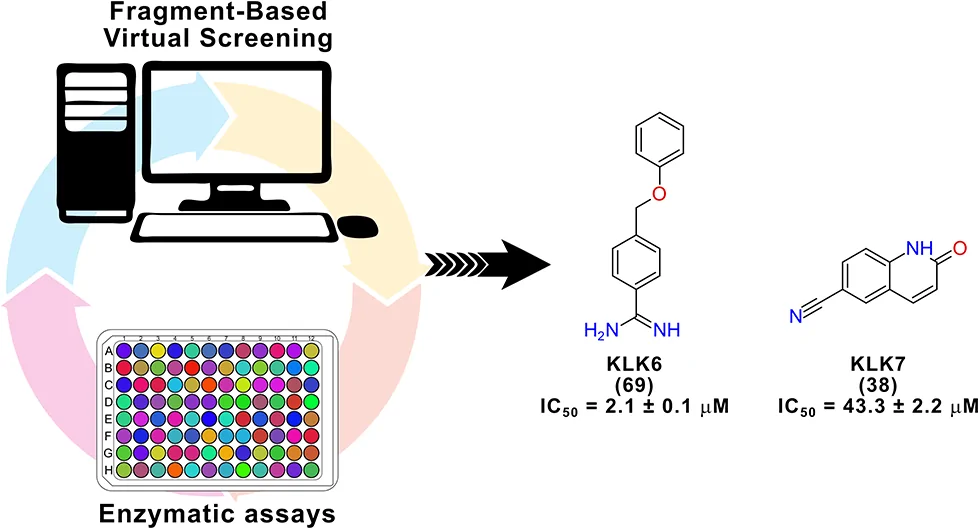

## Introduction

The enzymes KLK6 and KLK7 are members of the kallikrein-related peptidases (KLK) family, which encompasses 15 members (KLK1–KLK15).^1^ KLK7, together with KLK5, is best known for its involvement in desquamation, promoting the shedding of superficial corneocytes by degrading corneodesmosomal cadherins.^2,3^ In Netherton syndrome (NS), mutations in the SPINK5 gene result in the absence or drastic reduction of the serine protease inhibitor lympho-epithelial Kazal-type related inhibitor (LEKTI) expression.^4,5^ This increases the proteolytic activity of several KLKs, including KLK5 and KLK7, resulting in a skin disease characterized by severe inflammation and excessive skin scaling.^6^ KLK6 is the predominant secreted serine protease in the central nervous system and has been consistently associated with neurodegenerative disorders, including Alzheimer’s and Parkinson’s diseases, as well as multiple sclerosis.^7^ Beyond its elevated expression in pathological states, KLK6 has been shown in vitro to proteolytically process myelin basic protein, α-synuclein, and the β-amyloid precursor protein, thereby implicating it directly in demyelination and in the modulation of amyloidogenic and synuclein aggregation pathways.^8–10^ In addition to their involvement in neurodegenerative and skin diseases, KLK6 and KLK7 are also associated with the development of several types of cancer, such as pancreatic, colon, and ovarian cancer.^11,12^

With growing evidence that KLK6 and KLK7 dysregulation plays a key role in neurological diseases, skin diseases, and cancer, the development of inhibitors targeting these enzymes has emerged as a promising therapeutic strategy to modulate their activity and potentially mitigate the progression of these disorders. However, unlike other trypsin-like serine proteases, the development of kallikrein inhibitors has been lagging behind.^13–17^ When we started this project, small-molecule inhibitors of KLK6 were typically limited by modest potency, insufficient selectivity, or a peptidomimetic character. In contrast, better success has been achieved with the chymotrypsin-like KLK7, which has unique substrate specificity compared with other KLKs.

Traditionally, small-molecule inhibitors of KLK6 and KLK7 have been discovered through high-throughput screening, natural product sources, focused protease libraries, and virtual screening approaches.^14,16,18–23^ However, as serine proteases recognize extended peptide substrates along highly conserved binding grooves, achieving both high potency and subtype selectivity with small, drug-like molecules is inherently more challenging than for enzymes that catalyze well-defined, compact molecules. Here, we adopted fragment-based virtual screening (FBVS) as an alternative to the discovery route. Fragment-based screening has successfully enabled the identification of several serine protease inhibitors.^24–27^ The primary objective is often to identify alternative S1 binding groups that can replace highly basic moieties, such as amidines, which are prevalent in inhibitors of trypsin-like serine proteases. Fragment screening enabled the identification of a neutral heterocycle that binds within the S1 pocket of factor VIIa (FVIIa). Incorporation of these fragments into various chemotypes resulted in neutral, potent, selective, and permeable FVIIa inhibitors.^28,29^ Additionally, a fragment-based lead discovery (FBLD) strategy was employed to identify two novel FXIa P1 fragments, which were subsequently optimized to produce potent and selective inhibitors.^30^ Finally, screening a focused and diverse fragment library was instrumental in developing the first potent noncovalent reversible inhibitors of complement factor D.^31^

Although fragment-based screening is now established as a powerful tool to discover inhibitors for serine proteases, we describe here the first report of a fragment-based campaign against proteins of the kallikrein-related peptidase family. The group of fragments identified here may stimulate additional work to uncover alternative binding modes and chemically tractable starting points for targeting KLK6 and KLK7.

## Results and Discussion

### Retrospective Validation

The ability of rDock^32,33^ to dock fragment-like compounds was evaluated by redocking of fragments at the S1 pocket of KLK6 (PDB ID: 4D8N) and KLK7 (PDB ID: 5FAH). For KLK6, the root-mean-square deviation (RMSD) of the predicted binding pose compared to the experimental pose of the fragment was 0.7 Å (Figure S1). The top-scored pose of a manually constructed fragment inhibitor of KLK7 was in good agreement with its cognate X-ray counterpart, with an RMSD value of 1.3 Å (Figure S1). These results show that rDock successfully reproduced the experimental binding poses of both fragments, even with a more stringent RMSD cutoff of 1.5 Å.^34^

Next, the performance of rDock in discriminating between active and decoy compounds was evaluated using the receiver operating characteristic (ROC) curve and the Boltzmann-enhanced discrimination of receiver-operating characteristic (BEDROC). The area under the ROC curve (AUC-ROC) for KLK6 was 1.0 (Figure S2). The early retrieval was also excellent with a BEDROC value of 0.98. The discriminatory power of rDock for the KLK7 data set was also good (AUC = 0.75), but the early enrichment was relatively low (BEDROC = 0.15) (Figure S2). These findings indicate that rDock is suitable for use in prospective studies.

### Identification of Hit Fragments

To examine the chemical tractability of KLK6 and KLK7, a library with ∼1.5 million commercial fragments was virtually screened against the three enzymes. For the virtual screening of the library, we decided to limit the fragment docking only to the S1 pocket. This choice was based on the analysis of 375 crystal structures of serine proteases in complex with fragment ligands, including six kallikrein structures, which showed that fragment binding predominantly occurs within the primary specificity (S1) pocket. Figure S3 (Supporting Information) presents representative crystal structures of serine protease-fragment complexes illustrating this binding mode.

The rDock program^32,33^ was used to dock the fragment library into the KLK6 structure with PDB ID 4D8N.^21^ After removing duplicate molecules, the best 100000 were saved, and two strategies were used to further filter this data set. The first one was to select fragments that formed a neutral hydrogen bond with residue D189. Using a python script, 1104 fragments were selected, and only 369 were commercially available at our cutoff price (<100 USD). After a visual inspection of this subset, six molecules were selected (Figure 1a). The second strategy focused on the selection of fragments containing the amidine group linked to an aromatic ring. Among the top 100000 fragments, only 898 met this requirement. Of these fragments, 211 were commercially available and were grouped based on their maximum common substructure (MCS) using the libMCS program (ChemAxom). Finally, after a visual inspection, 12 fragments were selected for purchase (Figure 1a).

**Figure 1:**
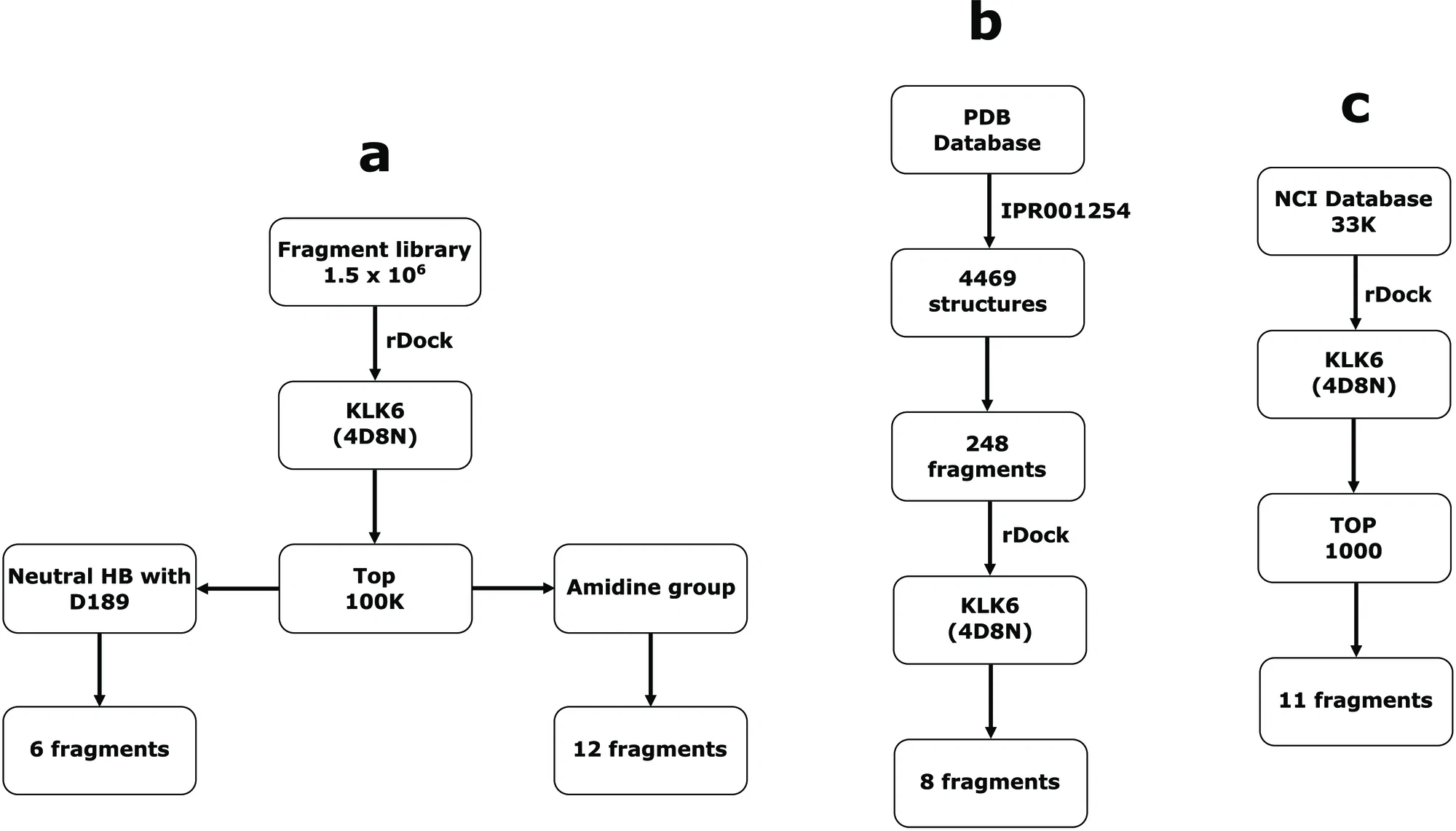
Fragment-based virtual screening workflow used to identify KLK6 inhibitors from (a) commercial fragment library, (b) PDB database, and (c) NCI database.

Kallikrein-related peptidases, like other (chymo)­trypsin-like serine proteases, have a similar substrate-binding site and a conserved tertiary structure (Figure S1). Fragments that bind to other trypsin-like serine proteases, especially in the conserved S1 pocket, are likely to interact with KLK6/KLK7 as well, thereby reducing its enzymatic activity. Thus, we took advantage of the large amount of structural information on serine proteases in complex with fragments available in the PDB database.^35^ For this purpose, a search was performed using the InterPro number IPR001254, which represents proteins that contain a trypsin-like serine protease domain. This search yielded 4469 structures, of which only those with a heavy atom count (HAC) of 5–18 were kept. Next, structures with irrelevant fragments (salts, carbohydrates and analogues, buffers, etc.), fragments with atoms other than C, H, N, O, S, X (halogen), without rings in the structure, and duplicates were removed. After applying these filters, the final collection containing 248 fragments was docked to the KLK6 structure (4D8N) using rDock, the results were visually inspected, and eight fragments were purchased (Figure 1b).

In addition to the commercial fragment library, a subset of the National Cancer Institute (NCI) database (https://dtp.cancer.gov/) containing only fragment-like compounds (∼33K) was docked against KLK6 using the structure 4D8N. After a visual inspection of the top 1000 compounds, 11 fragments were ordered from NCI for the *in vitro* activity testing (Figure 1c).

The 5FAH structure was used as the receptor for the docking of KLK7.^36^ The 20,000 fragments that had the best scores were selected, and this subset was filtered using four strategies. The first one consisted of the search for fragments that had a Cl or Br atom at a distance ≤ 4 Å from the side chain of residue A190. This search yielded 992 fragments, of which 534 were commercially available. These fragments were clustered, and 15 were selected after a visual inspection (Figure 2). A new search looked for fragments in which an ethynyl or nitrile group was located at a distance ≤ 3.6 Å from the side chain of amino acid A190. This search yielded 775 fragments, which 371 were commercially available. After a visual inspection of the results, 14 fragments were finally selected (Figure 2). We assumed that fragments containing a Me, Cl or Br atom in the vicinity of A190 would expel the water molecule in this region. This would lead to an additional increase in the binding energy of these fragments.

**Figure 2:**
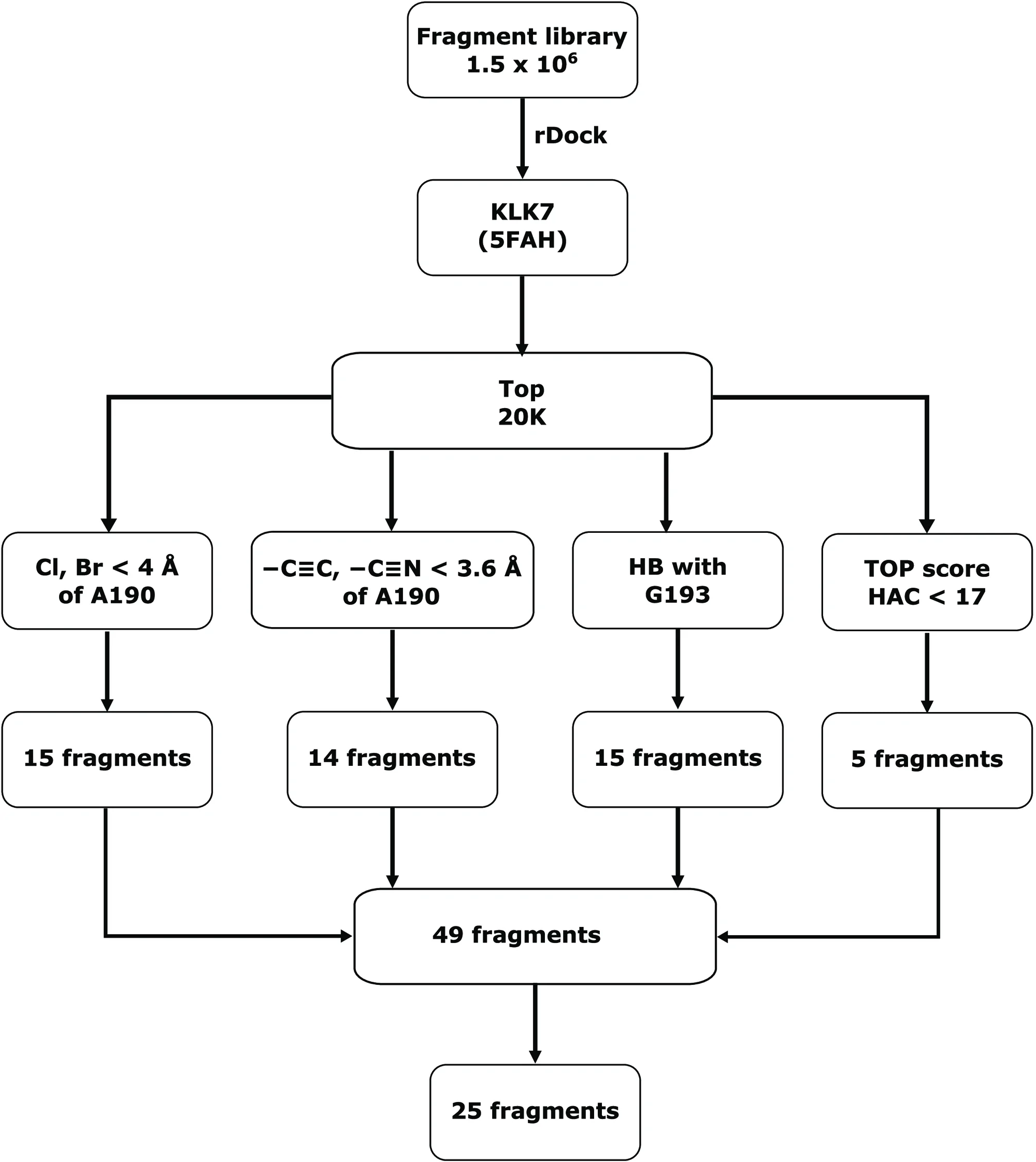
Fragment-based virtual screening workflow used to identify KLK7 inhibitors.

In the third selection, we looked for fragments that bind to the S1 pocket and form a hydrogen bond with residue G193 at the oxyanion hole. This search resulted in 1595 fragments, but only 877 were available. A visual inspection selected 15 fragments (Figure 2). The fourth selection focused on selecting the top-scoring fragments with HAC < 17. Of the 586 fragments found, 115 were available. After a visual inspection, five were selected (Figure 2). Finally, the 49 fragments selected for KLK7 according to the criteria above were combined, and 25 fragments were purchased after a final visual analysis of their poses and structures (Figure 2).

### Screening of the Fragment Library

#### KLK6 In Vitro Inhibition

The 37 fragments selected against KLK6 were initially evaluated in a single-point inhibition assay at 100 μM. Eleven compounds inhibited KLK6 by more than 30% and were subsequently subjected to IC_50_ determination, yielding eight compounds with IC_50_ ≤ 100 μM (hit rate = 21.6%). This set was also screened against KLK5, KLK7 and KLK8. Based on their chemical structure, these compounds were clustered into three groups: amidines (1–20), positively charged amines (21–26), and neutral amines (27–37) (Table 1 and Table S1).

**Table 1:**
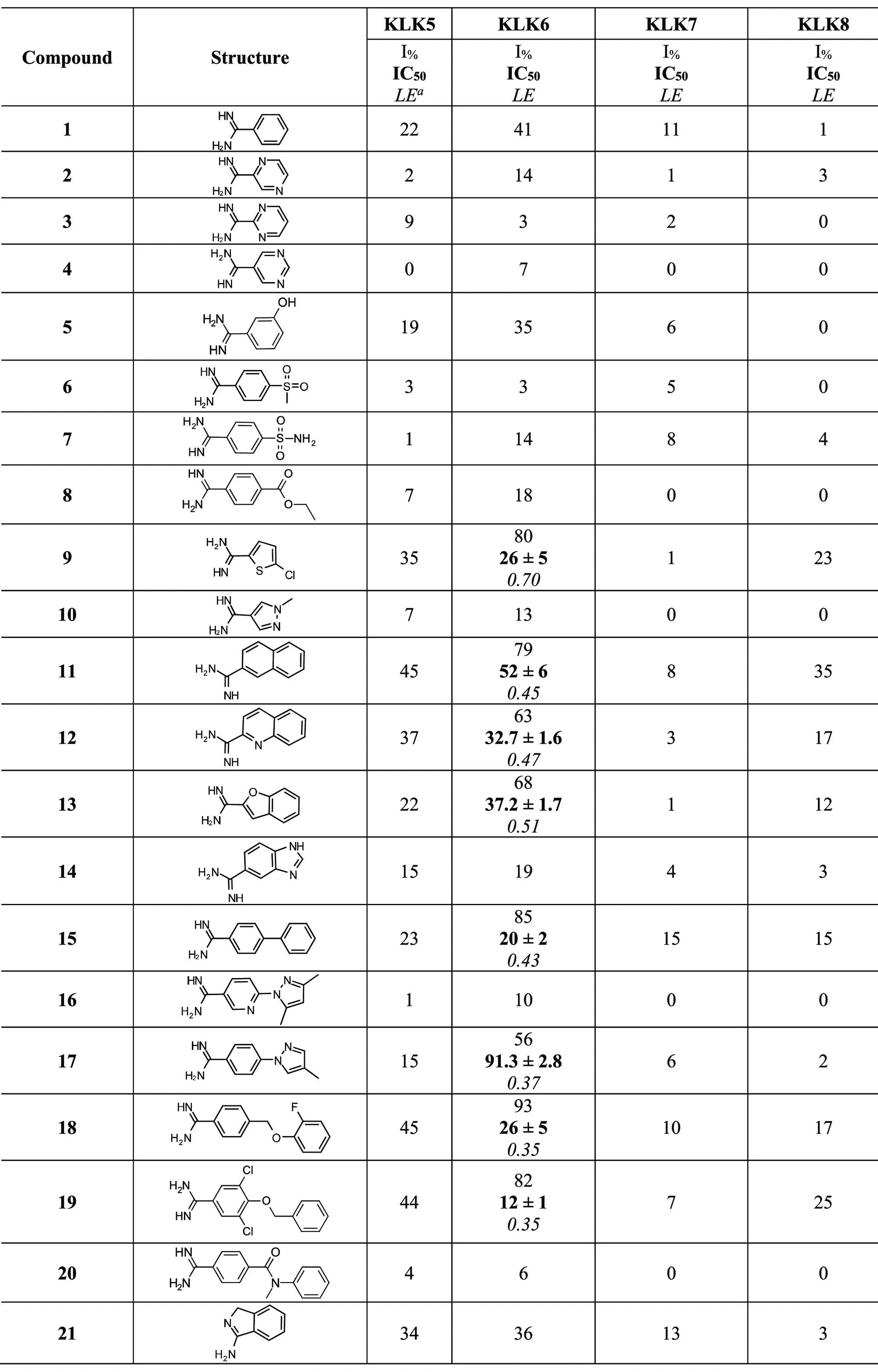
Structure, Percentage of Inhibition at 100 μM (I_%_), IC_50_ (μM) and Ligand Efficiency (LE) of Representative Fragments Selected against KLK6^a^

a Ligand efficiency (in kcal mol^–1^ atom^–1^) was calculated as LE = (1.37 × pIC_50_)/HA; HA = heavy atom; IC_50_ determination experiments were performed at least in duplicate, and the values are presented as mean ± SD.

Of the 20 fragments containing the amidine group, 10 inhibited KLK6 ≥ 30%. It is worth noting that although the S1 pockets of KLK5 and KLK6 are identical, only five fragments (**9**, **11**, **12**, **18**, and **19**) inhibited KLK5 ≥ 30%. A closer look reveals that the activity of these fragments against KLK5 is usually half that observed against KLK6. Also, only two residues in KLK8 differ from KLK6 in the S1 pocket: S190 and V213 are both replaced by threonine. Although these changes did not alter the pocket size, only fragment **11** inhibited KLK8 (I_%_ = 35%). This unexpected selectivity might be driven by differences in the electrostatic potential at or near the S1 pocket of these proteins.^37^

Based on their structure, the amidine fragments can be further grouped into three clusters: monocyclic (**1**–**10**), fused bicyclic (**11**–**14**), and isolated bicyclic (**15**–**20**). Benzamidine (**1**) was used as a positive control as it is a typical inhibitor of trypsin-like enzymes. Previously, this fragment was found to inhibit KLK5 with an IC_50_ of 501 μM.^38^ In another work, benzamidine inhibited KLK6 by 20% at 63 μM.^39^ These results are in agreement with the values reported here. The bioisosteric substitution of two carbon atoms in **1** by nitrogen in fragments **2**–**4** drastically reduces or abolishes the inhibition of KLK6. This shows that the electrostatic properties of the molecules as well as their shape influence the molecular recognition.^40^ As observed with complement factor D (FD), electrostatic interactions with D189 residue are not enough to provide high-affinity fragments for the three trypsin-like KLKs (5, 6 and 8) studied here.^41^

Compared with **1**, the introduction of a hydroxyl group (**5**) at the *meta* position of benzamidine slightly decreases the inhibition against KLK5/KLK6. However, the introduction of a methanesulfonyl (**6**), sulfonamide (**7**), or ethyl acetate group (**8**) eliminates the inhibition against KLK6.

Amidinothiophene **9** was the most active monocyclic fragment, inhibiting KLK6 with an IC_50_ of 26 μM. Additionally, it has a weaker inhibitory effect on KLK5 (35%) and KLK8 (23%), and was 2x more active against KLK6 than **1** (80% vs 41%). Previous research identified a class of KLK6 inhibitors with an amidinothiophene as the P1 group and a pyrrolidinone-sulphonamide scaffold.^22^ An X-ray structure of a compound from this series complexed with KLK6 revealed that the amidinothiophene moiety binds in the S1 pocket as expected. The best inhibitor in the series had an IC_50_ of 1.5 μM, which corresponds to a ligand efficiency (LE) of 0.20.

Fragment **9** is an attractive candidate for further optimization, since it has a high ligand efficiency (0.70), which is much higher than that of the inhibitor series discussed above. This can be explained by the fact that it is small, charged, and binds in a highly charged and well-buried binding site. The predicted binding pose of this fragment shows that it forms an electrostatic interaction with the carboxylate group of D189 and two hydrogen bonds with S190 and the backbone of N217 (Figure 3). Growing the fragment using chlorine as an exit vector could be a promising strategy to improve potency.

**Figure 3:**
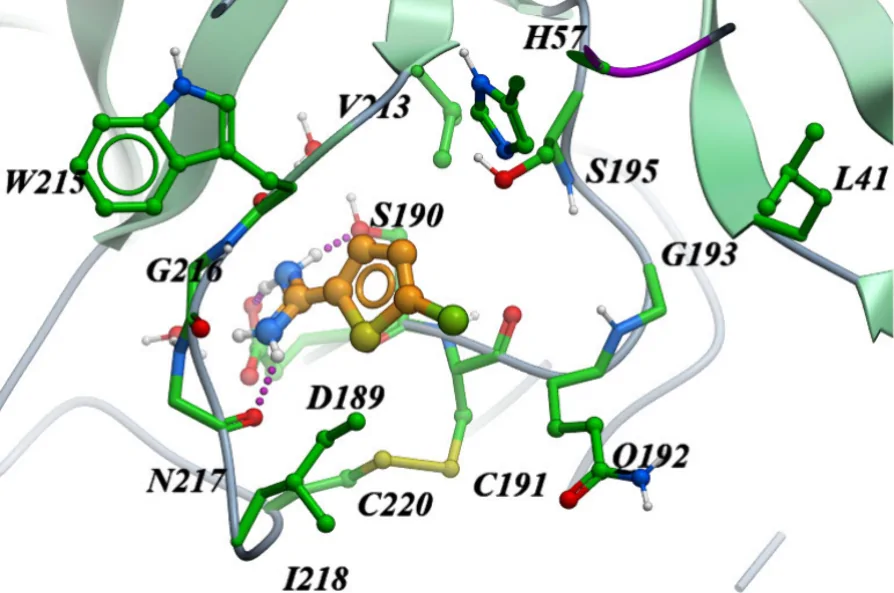
Predicted binding mode of fragment **9** (orange) against KLK6. Residues in the active site are shown as green sticks. Dashed lines represent intermolecular hydrogen bonds.

Three of the four fused bicyclic fragments (**11**–**13**) inhibit KLK6 by more than 60%. Fragment **11** has an IC_50_ and LE of 52 μM and 0.45, respectively. The introduction of a nitrogen into the aromatic ring in **12** results in a better IC_50_ (32.7 μM) and LE (0.47). Additionally, the benzofuran fragment **13** showed a potency similar to **12**, but with a higher LE.

Regarding the isolated bicyclic fragments (**15**–**20**), four (out of six) inhibited KLK6. Fragment **15** inhibited the enzyme with an IC_50_ and LE of 20 μM and 0.43, respectively. Replacing the terminal phenyl ring of **15** with a 4-methyl-1*H*-pyrazole (**17**) or 3,5-dimethyl-1*H*-pyrazole (**16**) results in a weak or inactive compound.

Fragments **18** and **19** with two phenyl rings separated by a two atoms linker, inhibited KLK6 with IC_50_ values of 26 and 12 μM, respectively. Both have the same LE (0.35), and they also inhibit KLK5 with lower potency. In a previous work, **18** inhibited thrombin by ∼50% at 100 μM.^42^ The other fragment of this group (**20**), with an amide linker was completely inactive.

The predicted binding poses of **18** and **19** are similar, with the benzamidine group binding at the S1 pocket, and forming the same interactions. The difference lies in the conformation of terminal phenyl ring. The analysis of the top docked poses generated by rDock (100 runs) shows that these two fragments can adopt two conformations: “down” or “up”. In the “down” conformation (**18**, Figure 4a), the 2-fluorophenol group is pointing to the oxyanion hole with the fluorine making interactions with the NH groups of G193 and S195. In the “up” conformation (**19**, Figure 4b), the terminal phenyl ring is directed toward H57. We did not observe a clear preference for one or another conformation.

**Figure 4:**
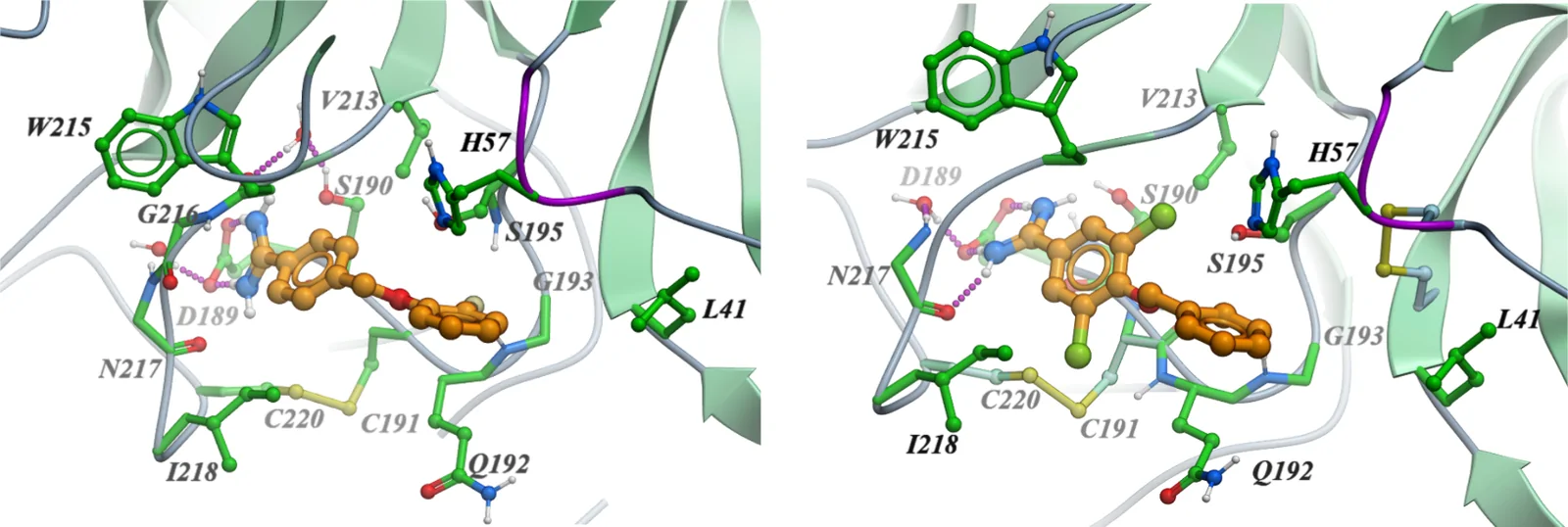
Predicted binding poses of fragments **18** and **19**. Both fragments can adopt a conformation where the terminal phenyl is pointing down (**18**) or up (**19**).

The screening of the fragments with a primary or secondary amine (**21**–**26**, Table S1) revealed that only one of them (**21**, Table 1) was active at 100 μM. This compound inhibited KLK5 and KLK6 by 34 and 36%, respectively. The structure of this fragment revealed that it actually has a masked amidine group with one of the nitrogen atoms cyclized in a five-membered ring. Compared to benzamidine (**1**), the cyclization led to a decrease in the calculated p*K*_*a*_ (calculated using MarvinSketch 23.16.0 by Chemaxon) of the nitrogen from 11.5 to 10.4. Previously, **21** was found to be an inhibitor of thrombin (*K*_d_ = 124 μM) and endothiapepsin (*K*_d_ = 1700 μM).^43,44^ Also, **21** was identified as a P1 group of furin, which was then incorporated into peptidic inhibitors, resulting in compounds with inhibition constants <10 pM.^45^ The crystal structures of **21** in complex with furin showed that it occupies an almost identical binding pose compared to benzamidine, and that its binding mode is maintained when it is introduced into the larger inhibitor.

None of the 11 fragments containing a neutral hydrogen bond donor inhibited the trypsin-like enzymes (KLK5, 6 and 8) by more than 30% when tested at 100 μM (Table S1). Fragments **29** and **36** were the only ones that showed some inhibition of KLK6 (20%) and KLK8 (25%). Interestingly, fragment **28** inhibited KLK7 by 31%. This enzyme has a chymotrypsin-like S1 pocket, where N189 and A190 replace D189 and S190, respectively, in the trypsin-like enzymes (KLK5, 6 and 8).^46^ This results in a more hydrophobic S1 pocket which is well suited for accommodation of fragments with polar groups, but not charged.

#### KLK7 In Vitro Inhibition

Of the 25 fragments selected against KLK7, three inhibited the enzyme by more than 30% at 100 μM: **38** (80%), **39** (44%) and **44** (54%) (Table 2). Fragments **38**–**40** are highly similar, and all have the 2-hydroxyquinoline ring in their structure. However, while **38** is potent and has high LE (IC_50_ = 43.3 μM; LE = 0.46), **39** is a weak KLK7 inhibitor, and **40** is inactive. An explanation for these differences in activity seems to be related to the substitution pattern of the 2-hydroxyquinoline ring. Fragments **39** and **40** have a methyl, and an ethoxy group at position 4 of the ring, respectively. On the other hand, **38** has a hydrogen at this position. The nitrile at position 6 also appears to be important for the activity, as its replacement by a bromine (**39**) or methoxy (**40**) reduces or abolishes the inhibition of KLK7. Nevertheless, both the nitrile and the 2-hydroxyquinoline ring are required for activity, since other fragments (**41**–**43**) that share structural similarities with **38** were inactive.

**Table 2:**
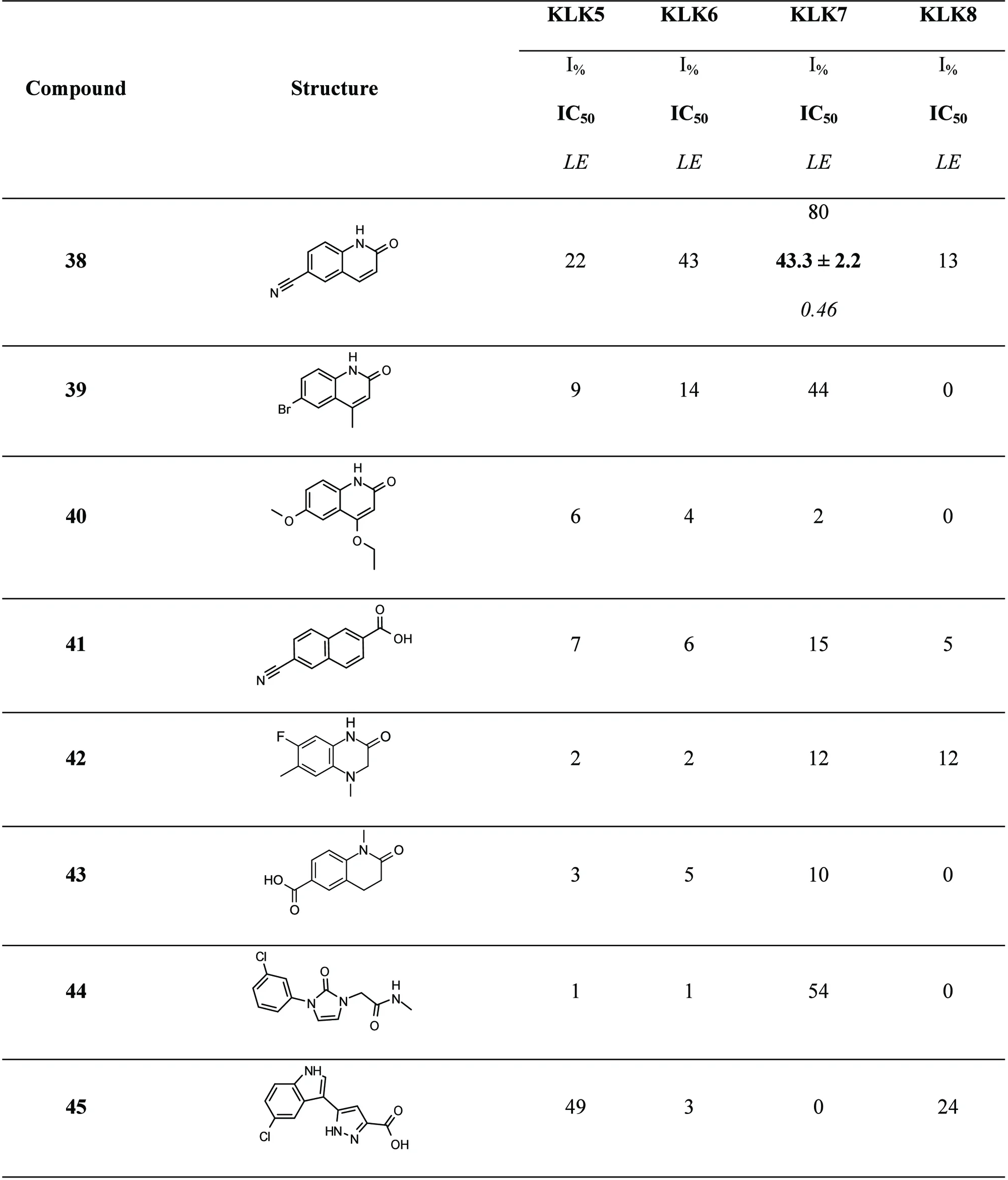
Structure, Percentage of Inhibition at 100 μM (I_%_), IC_50_ (μM) and Ligand Efficiency (LE) of Representative Fragments Selected against KLK7

In the predicted binding mode of **38**, the lactam is pointing toward N189, and the nitrogen forms a hydrogen bond with the backbone of T217 (Figure 5). The carbonyl of the fragment also forms two hydrogen bonds with water molecules located deeply in the S1 pocket. The nitrile is oriented toward the oxyanion hole and is ∼3.1–3.3 Å from the NH of S195 and G193.

**Figure 5:**
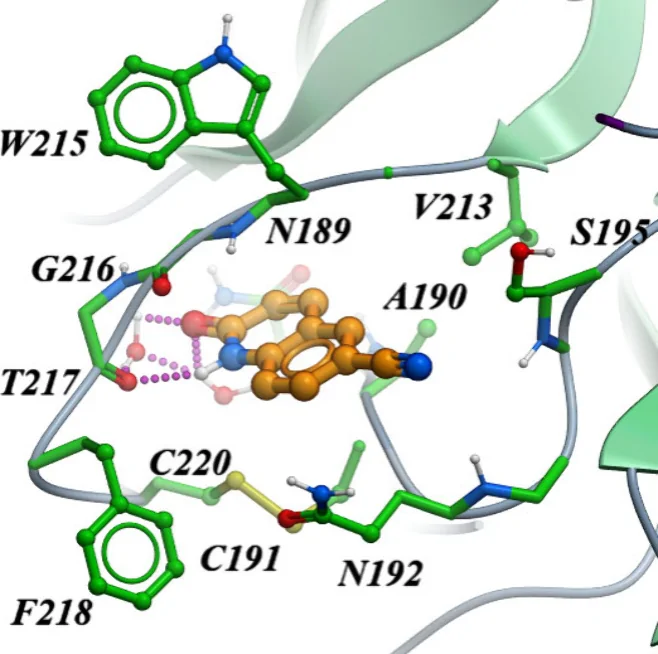
Predicted binding pose of fragment **38**.

It is worth mentioning that a fragment similar to **38** (without the nitrile group) was screened before against carbonic anhydrase, and showed low nM−μM inhibitory activity against many isoforms of this protein.^47^ Perhaps more relevant for the present work was that Fjellström et al. identified an analog of **38**, which has a chlorine substituting the nitrile, as an inhibitor of activated factor XI (FXIa) with a *K*_*i*_ of 70 μM (LE = 0.44).^48^ The crystal structure of this fragment was solved and showed that the chloro atom is directed almost perpendicular to the aromatic ring of Y228, and replaces a highly conserved water molecule (Figure S2a). An additional interaction with FXIa is observed between the quinolinone NH group and the backbone carbonyl of G218.

The S1 pockets of KLK7 and FXIa are very similar, and both have A190 replacing the typical serine. The difference is that N189, V213, and F218 in KLK7 are replaced by D189, T213, and G218, respectively, in FXIa (Figure S2b). These differences may explain why **38** shows a predicted binding mode in KLK7 distinct from its analog in FXIa. To further investigate this, we used rDock to dock **38** and save 100 poses. The top 47 poses share the same binding pose (“pose 1”), as shown in Figure 5, with an average RMSD of 0.2 Å and a mean docking score of −25.5 kcal/mol. Only two poses (out of 100) showed a binding mode of **38** similar to that of its analog in FXIa (“pose 2”), with an average RMSD of 1.0 Å. The stability of both binding poses (poses 1 and 2) was evaluated by running three independent molecular dynamics simulations (20 ns each). For pose 1, all three runs remain mostly within ∼1.0–2.0 Å RMSD (1.48 ± 0.13Å), which is generally consistent with a stable binding mode (Figure S3). In pose 2, the fragment RMSD values span a broader range than pose 1. While the mean RMSD is similar (1.56 ± 0.63 Å), large fluctuations up to ∼3.2–3.4 Å appear, particularly in run-3 and at the end of run-1 (Figure S3). This indicates a less stable binding mode. Finally, the MM/GBSA calculations yielded a more favorable average binding free energy for pose 1 (−20.5 ± 2.5 kcal/mol) than for pose 2 (−17.2 ± 0.5 kcal/mol). However, the observed difference of 3.3 kcal/mol is comparable to the uncertainty associated with MM/GBSA calculations and was not statistically significant across the three replicas. Therefore, the MM/GBSA results support, but do not conclusively establish, a preference for pose 1. Because conformational entropy was not included, the reported values should be regarded as relative binding free energies.

In one of our strategies to identify active fragments against KLK7, we selected fragments with the predicted ability to bind to the S1 pocket of the enzyme and extend into the oxyanion hole by forming hydrogen bonds with G193 or S195. To test these in silico predictions, we ordered 16 fragments (Table S2), but only fragment **44** (Table 2) showed activity at 100 μM against KLK7 (I_%_ = 54%). All other fragments were inactive or significantly weaker. Finally, fragment **45** inhibited KLK5 (I_%_ = 49%) and KLK8 (I_%_ = 24%), but not KLK7.

### SAR by Catalog

Based on the preliminary SAR analysis, the larger number of active KLK6 fragments identified in our screen and the low number of KLK6 inhibitors currently reported in the literature, we decided to pursue a hit expansion campaign for seven inhibitors (**9**, **11**, **12**, **15**, **17**, **18**, and **19**) of this enzyme. Similarity and substructure searches of commercially available analogs were performed following the “SAR by catalog” approach.^49^ Molecules with an identical substructure as the parent fragment were searched using Arthor (arthor.docking.org) and ChemSpace (https://chem-space.com).^50^ Searching for similar molecules within the Enamine REAL database was conducted using SmallWorld (sw.docking.org).^50^

The searches failed to find interesting analogs for fragment **9**, and returned only one analog for the fragment match-pair **11**–**12** (**62**) and one for **15** (**63**) (Table 3). Among the fragments **11**, **12**, and **62**, the presence and the position of a nitrogen in the ring affects the activity. When the nitrogen is absent (**11**) or at position 2 (**62**) the compounds display similar potency. However, the fragment **12**, which has the nitrogen at position 1 is ∼1.5× more potent than the previous two fragments. Compared with **15**, the potency of **63** is almost 4× lower (20 vs 72.9 μM). These results are in line with our previous observations that the isosteric substitution of carbon by nitrogen in the aromatic rings results in weaker inhibition of KLK6.

**Table 3:**
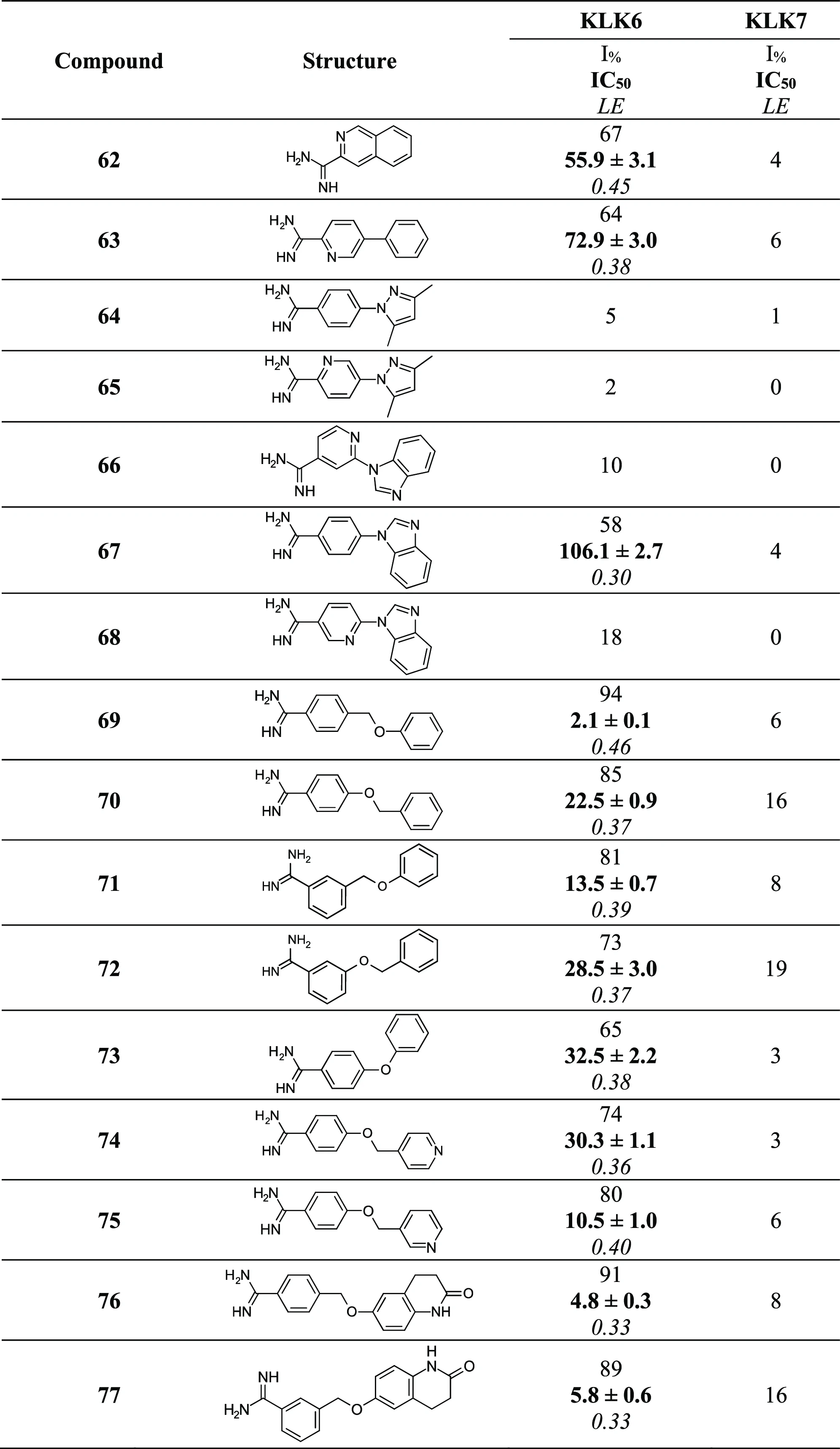
Structure, Percentage of Inhibition at 100 μM (I_%_), IC_50_ (μM) and Ligand Efficiency (LE) of the KLK6 Hit Analogs

We tested five analogs (**64**–**68**) of fragment **17** for their activity against KLK6 (Table 3). Very closely analogs of **17** such as **64** and **65**, were completely inactive against KLK6. Only **67** was active with an IC_50_ of 106.1 μM. Fragment **68**, which differs from **67** by only a central ring carbon substitution for a nitrogen was almost inactive.

The seven analogs of **18** and **19** were all active against KLK6 (Table 3). Fragment **69**, which is the unsubstituted analog of **18**, was ∼12-fold more potent (IC_50_ = 2.1 μM). Removing the two chlorines in **19**, as seen in **70** (IC_50_ = 22.5 μM), resulted in 2-fold loss of potency. The fragments **69** and **70** are constitutional isomers, and they differ in how the ether group is bonded to the benzamidine group. In **69** the substituent is a phenoxymethyl group, while in **70** it is a benzyloxy group. Although small, this difference is enough to make **69** ∼ 11 times more potent than **70**. In a previous work, **69** and **70** showed antitrypanosomal activity (IC_50_ = 3.71 and 1.84 μM, respectively) against *Trypanosoma brucei rhodesiense*.^51^ Also, **69** was found to be a reversible competitive inhibition (*K*_*i*_ = 2.6 μM) of boar spermatozoa acrosin.^52^

To further understand the increased potency of **69** vs **70**, we calculated some properties important to drug design. Properties such as MW (226.3 Da), HBD (2), HBA (1), RB (4), and PSA (59 Å^2^) were equal. Similarly, logP (2.22 vs 2.47) and logD (0.46 vs 0.57) values were almost identical. Also, we performed an exhaustive docking for each fragment (*N* = 100 runs) with rDock. The results showed that two clusters correspond to 70 and 83 poses of **69** and **70**, respectively. The most frequent cluster features the fragments in an extended conformation (Figure S7). In the second most frequent cluster, the fragments adopt an L-shape conformation (Figure S7). Since the activity drop of **70** could not be explained from the calculated properties or the exhaustive docking, we suspected ligand strain as a potential cause. Quantum-mechanical (QM) calculations indeed revealed a major difference in the energy profile of torsion *T*_1_ (Figure 6a) between the two compounds. The calculated torsion energy profile of **69** shows a weak rotameric preference, implying low rotational barriers and fewer restrictive conformations. The global minimum occurs at *T*_1_ = 205°, while *T*_1_ = 270° is just 0.6 kcal/mol above the minimum. The preferred low-energy conformation of **70** is at *T*_1_ = 180°, indicating a strong preference for the ether substituent to be in plane with the aromatic ring system, which orients the oxygen lone pair to interact with the aromatic π cloud (Figure 6a). The proposed bound structure of **69** has a *T*_1_ = 121° (Figure 6b), whereas forcing **70** to adopt this conformation would result in a considerable ligand strain (2.9 kcal/mol). Finally, the electronegative oxygen atom and the inherent C–O dipole moment affect the dipole moment of benzamidine group in **70**, which could also be contributing to the reduced potency of this fragment.

**Figure 6:**
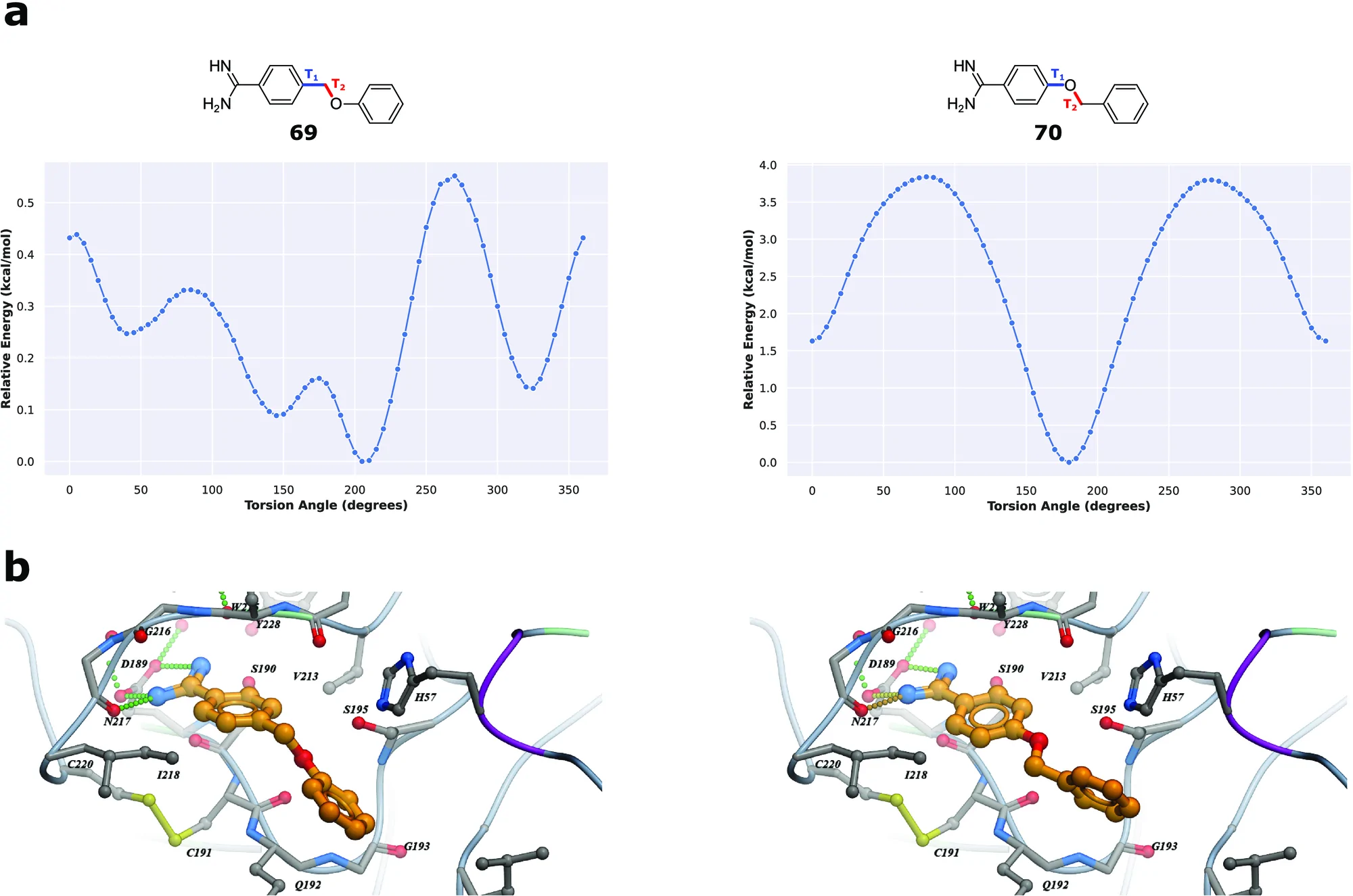
(a) Quantum mechanical (B3LYP/6-311G**) torsion scan for compounds **69** and **70**. Torsion *T*_1_ (blue) was scanned from 0 to 360° in steps of 5°. Torsion *T*_2_ (red) was kept constant at −180°. (b) Predicted binding mode of fragments **69** and **70**. The torsion *T*_1_ in **69** and **70** are 121° and 178°, respectively.

Next, we evaluated the effect of moving the ether group to the *meta* position. The potency of **69** decreases approximately seven-times when the phenoxymethyl group moves to the *meta* position in the fragment **71**. Compared with **70**, a less pronounced reduction in potency was also observed for **72** (IC_50_: 22.5 μM vs. 28.5 μM), which has the benzyloxy group at the *meta* position.

A significant decrease in potency was observed when the two-atom linker was reduced to one in fragment **73**. In addition, replacing the distal phenyl ring with pyridine has contradictory effects in the analogues of fragment **70**. When the nitrogen is at the *para*-position (**74**) in relation to the ether linker, the potency decreases. But when the nitrogen is at the *meta*-position (**75**), the potency increases by around two times.

Based on the promising results with **69** (IC_50_ = 2.1 μM; LE = 0.46), two additional compounds were ordered to expand the fragment and form new interactions within adjacent subpockets. The substitution of the distal phenyl ring by tetrahydroquinolin-2-one in compound **76** increased the heavy-atom (HA) count to 22. Despite this, the potency/LE of this compound (IC_50_ = 4.8 μM; LE = 0.33) was slightly lower than that of **69**, which has only 17 HA. Compound **77** has the methoxy-tetrahydroquinolin-2-one group in the *meta* position and shows a potency similar (IC_50_ = 5.8 μM) to that of **76**. Compared with **71** (its phenyl match-pair), **77** was ∼2× more potent, but had a lower LE (0.39 vs 0.33). These results indicate that replacing the phenyl ring with tetrahydroquinolin-2-one fails to capture additional interactions with KLK6, and further medicinal chemistry optimization of **69** into a lead-like compound is warranted.

### KLK6 Mechanism of Inhibition

Following initial IC_50_ determination for hit prioritization, compounds **69**, **76** and **77** were selected for independent mechanistic and kinetic characterization (Figure 7). A jump dilution (1:100) of the enzyme–inhibitor complex instantly restored over 85% of enzyme activity, indicating a reversible noncovalent inhibition of KLK6 by these compounds. Dixon plots were generated to identify the type of inhibition and experimentally determine the inhibition constant (*K*_*i*_). The plots for **69**, **76**, and **77** showed all straight lines crossing above the *x*-axis, confirming competitive inhibition, which was also supported by Lineweaver–Burk plots (not shown). The *K*_*i*_ values were around 7.5 (**69**), 8 (**76**), and 10 μM (**77**).

**Figure 7:**
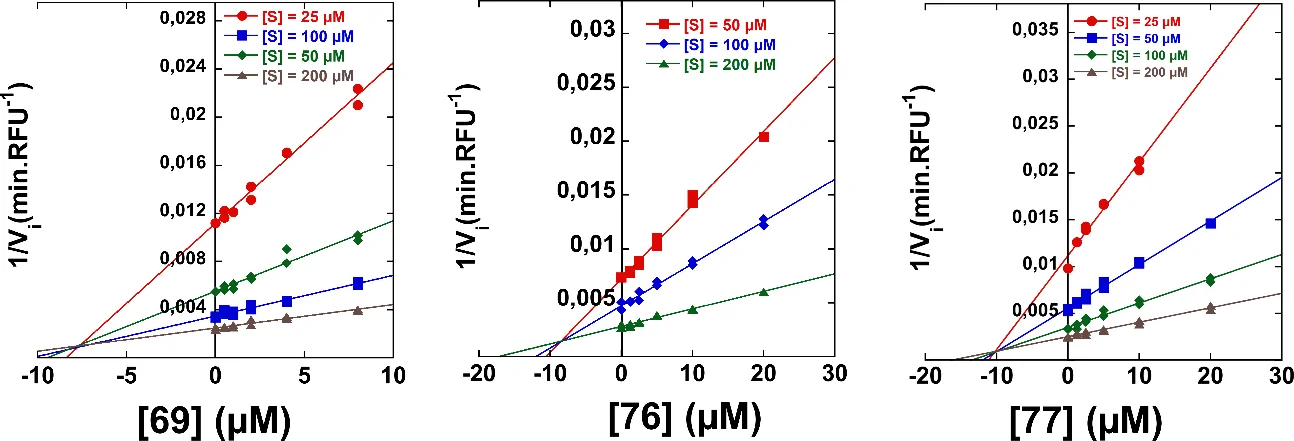
Mechanism of KLK6 inhibition by compounds **69**, **76**, and **77**. Dixon plot with Boc-QAR-AMC (25 to 200 μM) in 50 mM Tris, 1 M citrate, 0.05% Brij-35; pH 7.

### KLK6 Selectivity Profiling

Selectivity toward KLK6 of compounds **69**, **76**, and **77** was first assessed at 20 μM against a restricted panel of closely related kallikreins (KLK1, KLK4, KLK5, KLK7, and KLK8). Under these conditions, compound **69** displayed a clear preference for KLK6, with minimal inhibition of the other KLKs (<10%), suggesting a favorable selectivity window within this limited panel (Table 4). Compounds **76** and **77** also inhibited KLK6 strongly (85.6% and 97.2%, respectively) as expected, although a more pronounced cross-inhibition of KLK5 was observed.

**Table 4:** Percentage Inhibition of KLK1, KLK4, KLK5, KLK6, KLK7, and KLK8 by Compounds **69**, **76**, and **77** Determined at 20 μM under Standard Assay Conditions^a^

		inhibition at 20 μM (%)				
compound	KLK1	KLK4	KLK5	KLK6	KLK7	KLK8
**69**	<5	<5%	11	72.7	<5	7.5
**76**	23.1	nd	58.9	85.6	<5	16.1
**77**	6.9	nd	67.0	97.2	12.1	5.2

a nd: not determined.

While such restricted panels are commonly used in the literature to illustrate selectivity within the KLK family, they may overestimate subtype discrimination (Figure 8a). Therefore, compound **69** was further evaluated against a broader panel of trypsin-like serine proteases (Figure 8b). *K*_*i*_ measurements confirmed a preference for KLK6 over KLK5, KLK14, plasmin and matriptase, with selectivity ratios ranging from ∼4- to ∼ 9-fold. In contrast, compound **69** displayed comparable potency toward trypsin 3, highlighting the challenge of achieving selectivity within this highly conserved protease family.

**Figure 8:**
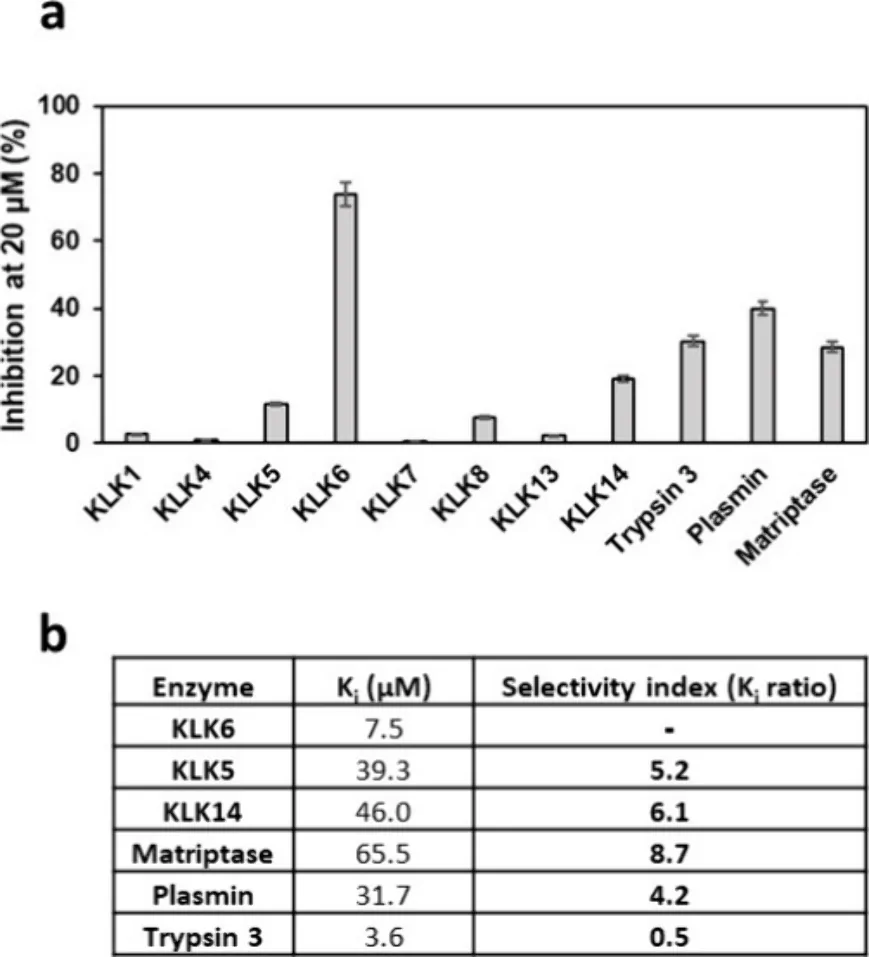
Selectivity profile of compound **69**. (a) Single-point inhibition measured at 20 μM against a panel of KLKs and additional trypsin-like serine proteases. (b) *K_i_* values determined by kinetic analyses for KLK6 and selected proteases from the initial screening. Selectivity indices correspond to the ratio *K*_*i*_^protease^/*K*_*i*_^KLK6^.

## Conclusion and Future Implications

This study reports the first fragment-based virtual screening campaign targeting members of the kallikrein-related peptidase family. By integrating large-scale structure-based fragment docking with focused enzymatic validation, we identified chemically simple and ligand-efficient fragments engaging the conserved S1 pocket of KLK6 and KLK7. Fragment **69**, in particular, emerged as a noncovalent competitive KLK6 inhibitor with low micromolar potency and high ligand efficiency, establishing a robust starting point for further optimization. Additional kinetic profiling revealed a ∼4- to ∼9-fold preference for KLK6 over KLK5, KLK14, plasmin and matriptase, while also highlighting residual cross-reactivity with trypsin 3. Such cross-reactivity is consistent with the conserved nature of the Asp189 S1 pocket among trypsin-like serine proteases, while differences in the surrounding binding site likely contribute to the distinct inhibitory profiles observed across the protease panel.

The predominance of benzamidine-derived KLK6 hits further illustrates the central role of this binding region in fragment recognition. While this canonical interaction efficiently anchors small ligands, the intrinsic basicity and polarity associated with benzamidine chemotypes may limit downstream drug-like properties. Overcoming this limitation will likely require moving beyond exclusive reliance on the primary specificity pocket.

Emerging structural and computational studies on multimeric enzymes suggest that conformational plasticity and transiently formed subsites, so-called *cryptic* pockets, can be revealed upon ligand binding or through dynamic fluctuations of surface loops.^53–55^ Although the detection of cryptic pockets in monomeric enzymes such as serine proteases remains challenging due to limited large-scale conformational rearrangements, recent advances in deep learning–based structure prediction and AI-driven dynamics analysis are increasingly enabling the identification of transient, druggable binding sites.^56–58^ Specifically, flexible loop areas shaping the S2/S3 subsites or solvent-exposed clefts may provide opportunities to access less conserved structural features and improve subtype selectivity. Fragment-based approaches are particularly well-suited to uncover these shallow or transient pockets due to their ability to detect low-affinity binding events that may not be apparent in traditional screenings.

The KLK7 campaign yielded a limited number of active fragments, which may reflect the chemical space explored in the present study and, consequently, limited the extent of characterization reported here. Nevertheless, validated hits were identified through complementary screening strategies, providing a foundation for future optimization studies.

Collectively, these findings demonstrate that KLK6 and KLK7 are tractable targets for fragment-based discovery and provide ligand-efficient starting points that can be further elaborated to probe both canonical and cryptic binding opportunities. Such strategies may ultimately enable the development of selective and pharmacologically viable kallikrein inhibitors.

## Materials and Methods

### Materials

All of the reagents and solvents were purchased from commercial vendors and used without further purification. Screened compounds were obtained from MolPort and ChemSpace. All purchased hit compounds were used without any further purification. The purity and identity were confirmed by the spectroscopic data provided by the suppliers (see the Supporting Information).

### Virtual Library

A virtual library of compounds was built using compounds from Aldrich Market Select, Molport, and ZINC20. Initially, the molecules present in each of the three databases were downloaded and saved in a single file in the SMILES format (∼29 million compounds). Then, we used the software Standardizer (ChemAxon) to standardize the compounds. In this step, explicit hydrogen atoms and certain fragments (counterions, solvents, etc.) were removed, as well as any information about the stereochemistry of the molecule. The charged molecules were converted into their neutral forms and the aromatic rings and tautomers were represented in a canonical form. The resulting collection contained approximately 11.6 million compounds. Next, the Filter program (OpenEye) was used to select compounds that follow the rule of three.^59^ In the next step, the Lilly Medchem-Rules program was used to remove undesirable structures.^60^ Those that passed this filter were then submitted to the OMEGA software (OpenEye) to generate a single low-energy conformation.^61,62^ The final collection of compounds has approximately 1.5 million compounds.

We also used the 284 K chemical library obtained from the Developmental Therapeutics Program, National Cancer Institute (DTP-NCI) (https://dtp.cancer.gov/) to perform a virtual screening against KLK6. This library was prepared by using the same protocol discussed above.

### Molecular Docking

After a search in the Protein Data Bank, two crystallographic structures were selected for docking studies.^63^ These structures are 4D8N (KLK6) and 5FAH (KLK7).^21,36^ Then, for each structure, the program Protoss was used to add hydrogen atoms, determine protonation states and tautomers, and remove alternative conformations of the residues.^64^ The SZYBKI program (OpenEye) was used to minimize the structure of the complex, to refine the position of the hydrogen atoms, and to remove structural stresses.^65^ All water molecules were removed, except those located in the active site and containing two or more hydrogen bonds with amino acid residues.

Docking calculations were carried out with rDock.^32,33^ The binding site, which was restricted to the S1 pocket of the proteins, was defined based on the cocrystallized inhibitor using the reference ligand method, with a cavity radius of 6.0 Å and a small sphere radius of 1.5 Å. Cavity mapping employed a 0.5 Å resolution grid and the RbtCavityGridSF scoring function. For KLK6, pharmacophoric restraints were applied to residue D189 to ensure that the generated poses have the hydrogen bonds interactions observed in the crystallographic structures between the inhibitor and enzyme. The standard docking protocol, i.e., three stages of Genetic Algorithm (GA) search (GA1, GA2 and GA3), followed by low temperature Monte Carlo (MC) and Simplex minimization (MIN) stages, was used to generate low energy ligand poses. Docking scores were calculated by the default scoring function (SF3). Each compound was subjected to 25 independent docking runs.

### Torsion Scan

A dihedral angle scan was carried out using the python script Psi4PerformTorsionScan.py available in the MayaChemTools package.^66^ This script uses PSI4 and RDKit to perform a torsion scan around torsion angles specified using SMILES/SMARTS patterns.^67,68^ The torsion T_1_ was scanned from 0 to 360° in steps of 5° for both compounds (**69** and **70**). At each interval, a constrained geometry optimization was carried out at B3LYP/6–31G**. For both systems, the dihedral angle *T*_2_ was kept constant to 180°.

### Enzyme Kinetics Assays

Recombinant human kallikreins and proteases were obtained from Bio-Techne and used at a concentration of 1 nM or less. Fluorogenic peptide substrates (Bachem and AAT Bioquest), H-PFR-AMC (for KLK1), Boc-VPR-AMC (for KLK4, KLK8, KLK13), Boc-QAR-AMC (for KLK5, KLK6, KLK14, trypsin 3, matriptase), D-VLK-AMC (for plasmin) and MeO-Succ-RPY-AMC (for KLK7) all linked to the fluorophore 7-amino-4-methylcoumarin (AMC), were used. All assays were conducted in kinetics buffer 50 mM Tris, Citrate sodium 1M, pH 7.0 supplemented with Brij-35 0.05% to minimize compund aggregation. *K*_*M*_ values determined for the enzyme–substrate pairs used under these conditions are presented in Table S3. Inhibition assays were performed in triplicate over a range of inhibitor concentrations. Enzymes and inhibitors were preincubated for 15 min at 37 °C before adding the fluorogenic substrate. The initial reaction rate without inhibitor (*V*_0_) was set as 100% activity. Inhibition was calculated as % inhibition = 100 × (1 – *V*_i_/*V*_0_), where *V*_i_ is the initial rate in the presence of the inhibitor. IC_50_ values were determined for hit validation and compound prioritization. Selected compounds were subsequently characterized by independent kinetic experiments to determine *K*_*i*_ values. IC_50_ (concentration required for 50% inhibition) values were obtained by fitting the data to the equation: % inhibition = 100 × [I]/(IC_50_ + [I]). To assess reversibility, the jump dilution method was applied: enzyme–inhibitor mixtures were preincubated for 15 min and then diluted 100-fold into substrate-containing buffer. Residual activity was measured under standard assay conditions. Noncovalent inhibition mechanism and inhibition constant (*K*_*i*_) were determined by varying both substrate and inhibitor concentrations. Classical Lineweaver–Burk and Dixon plots were used for kinetic analysis. All experiments were independently performed at least two times and data were analyzed with Kalleidagraph software. No interference with AMC fluorescence was detected for hit compounds under assay conditions (Figure S8).

## Supplementary Material





## Data Availability

The data sets used (Aldrich Market Select, Molport, ZINC, and DTP-NCI) are publicly available. The software tools employed in this study, including RDKit (https://www.rdkit.org/), rDock (https://rdock.github.io/) MayaChemTools (https://www.mayachemtools.org/) are all freely accessible on their respective Web sites. OpenEye and ChemAxon are available through academic licenses.

## Notes

Retrospective validation protocol; Detailed molecular dynamics protocol; Tables with the structure and activity data for all compounds (Table S1 and S2); A figure showing the redocking (Figure S1) and ROC-AUC (Figure S2) results; A figure showing representative structures containing a trypsin-like serine protease domain (Figure S3); A figure comparing the tertiary structure of KLK6 against KLK7 and other serine proteases (Figure S4); A figure comparing the active site of KLK7 and factor XIa (Figure S5); A figure showing the RMSD of fragment **38** during the MD simulation (Figure S6); A figure showing the poses of the two most frequent cluster obtained after an exhaustive docking of fragments **69** and **70** (Figure S7); A figure showing fluorescence controls for compounds **38**, **69**, **76** and **77** under assay conditions (Figure S8). HRMS and HPLC data of selected hit compounds (Appendix I) (PDF)
Molecular formula strings of all compounds reported here, along with biological potency data (XLSX)
