# Supplementary material for: Fragment-Based Discovery of KLK6 and KLK7 Inhibitors
Source: J Chem Inf Model. 2026 Aug 5;66(17):11389–402. doi: 10.1021/acs.jcim.6c02059 (PMC13580116; doi:10.1021/acs.jcim.6c02059)
Supplement: Supplementary file 1 [file ci6c02059_si_001.pdf]

Support Information

Fragment-Based Discovery of KLK6 and KLK7 Inhibitors

Renato Ferreira de Freitas[1,2]*, Feryel Soualmia[3,4]*, Riles Boumali[3,4], Yasmine Harrak[3,4], Chahrazade El Amri[3,4]

[1]Centro de Ciências Naturais e Humanas, Universidade Federal do ABC, Rua Arcturus 3, São Bernardo do Campo, São Paulo, 09606-070, Brazil.

[2]Programa de Pós-Graduação em Ciências Farmacêuticas, Escola de Farmácia, Universidade Federal de Ouro Preto, Ouro Preto, Minas Gerais, 35400-000, Brazil.

[3]Sorbonne Université, CNRS, Inserm, Development, Adaptation and Ageing, Dev2A, F-75005 Paris, France.

[4]Sorbonne Université, CNRS, Inserm, Institut de Biologie Paris-Seine, IBPS, F-75005 Paris, France.

*Correspondence to: renato.f@ufabc.edu.br; feryel.soualmia@sorbonne-universite.fr

Supplementary methods

Retrospective validation

Redocking

The co-crystallized ligand from the KLK6 complex (PDB ID: 4D8N) was fragmented, and the resulting moiety was utilized for the redocking study with rDock.[1] We selected the structure of KLK7 in complex with a pyrrolidine-based compound (PDB ID: 5FAH).[2] We then fragmented this inhibitor and used the portion that binds to the S1 pocket for redocking. Starting with the SMILES of each fragment, a random ligand conformation, generated using OMEGA (OpenEye), was used for the docking calculations. The steps of protein and ligand preparation, as well as the rDock parameters, were the same as those described in the Methods section of the manuscript.

We calculated the root-mean-square deviation (RMSD) values between the top-scoring pose and the X-ray structure for each fragment using the open-source tool obrms from OpenBabel.[3]

ROC-AUC analysis

For the validation of rDock, a data set with six fragment inhibitors (pchembl: 4 – 8.8) of KLK6 (CHEMBL4448) was selected from the ChEMBL database.[4] For KLK7 (CHEMBL2443), 18 fragments were retrieved from ChEMBL. DUDE-Z decoys were generated for each of the 24 fragments.[5]

To quantify enrichment, we used the area under the curve (AUC) of the receiver operator characteristic (ROC) curve, which plots the true positive rate (% of ligands found) versus the false positive rate (% of decoys found). The *pROC* package was used to generate and analyze the ROC-AUC curves.[6] The R package *enrichvis* was used to calculate the Boltzmann-enhanced discrimination of receiver operating characteristic (BEDROC), which is used to assess the "early recognition".[7]

Molecular dynamics simulation

The docked poses of the ligand in complex with KLK7 were used as a starting conformation for the minimization and MD simulation. Missing atoms and hydrogens were added using the tleap program in Amber24. Amber ff99SB force field and GAFF2 force field were assigned to amino acid residues and to the ligands, respectively. [8] The GAFF2 parameters were generated using antechamber and parmchk programs. Partial atomic charges were derived using AM1-BCC methodology.[9] Each complex structure was solvated in an OPC water octahedral box extending at least 10 Å from the complex, and then neutralized by the addition of an appropriate number of monovalent counter-ions.

Each system was relaxed by two successive energy minimizations in pmemd program. First, the heavy atoms of the solute were kept fixed with 10.0 kcal/mol/Å^2 force constant while the hydrogen atoms and waters were relaxed by 1000 steps of steepest descent before switching to the conjugate gradient algorithm for the remaining 4000 steps. Then all atoms were relaxed using the same protocol.

Starting from the minimized system, each complex was equilibrated by five successive steps. First, it was gradually heated from 0 K to 300 K during 100 ps with a restrain force constant of 10.0 kcal/mol/Å^2 applied to the heavy atoms of the solute under NVT conditions. Next, a 100 ps MD simulation with a 10.0 kcal/mol/Å^2 restraint on the complex was carried out at a pressure of 1 atm and 300 K to equilibrate the density using Berendsen's barostat.[10] Finally, the complex was equilibrated through four additional 100 ps at 300 K and 1atm with a restraint force constant of 8.0, 6.0, 4.0 and 2.0 kcal/mol/Å^2,

respectively, applied to the heavy atoms of complex. The temperature was kept fixed at 300 K using a Langevin thermostat with a collision frequency of 2 ps^{-1}.[11]

After the equilibration phase, three independent molecular dynamics simulations of 20 ns each, at constant pressure with a target temperature of 300 K and pressure of 1 atm was conducted in pmemd.cuda program.[12] The SHAKE algorithm was employed to constrain all hydrogen atoms and the time step was set to 2.0 fs. [13] Particle mesh Ewald (PME) method was used to treat the long-range electrostatic interactions with a default cut-off of 8.0 Å. [12] Both energies and coordinates were saved every 5 ps, thus yielding an MD trajectory of 4000 frames, which was used in the post-production energetic analysis.

MMBGSA

In this work, a single trajectory approach was adopted in the calculation of the binding free energies between the receptor and inhibitor using 2000 snapshots. The MMPBSA.py script implemented in AmberTools 25 was used to perform the MM/GBSA calculations. All the required steps to estimate the binding free energy of the protein-ligand complexes are automatically performed with this script. The electrostatic portion of ΔG was calculated using the Generalized Born method (igb=66).[14] The nonpolar contribution was determined based on the solvent accessible surface area (SASA) using the LPCO method, where the surface tension constant was set to 0.0072 kcal/mol/$Å^2$. For simplicity, all changes in solute entropy were omitted.

Supplementary References

(1) Liang, G.; Chen, X.; Aldous, S.; Pu, S.-F.; Mehdi, S.; Powers, E.; Xia, T.; Wang, R. Human Kallikrein 6 Inhibitors with a Para-Amidobenzylanmine P1 Group Identified through Virtual Screening. *Bioorg. Med. Chem. Lett.* **2012**, *22* (7), 2450–2455. https://doi.org/10.1016/j.bmcl.2012.02.014.

(2) Maibaum, J.; Liao, S. M.; Vulpetti, A.; Ostermann, N.; Randl, S.; Rüdisser, S.; Lorthiois, E.; Erbel, P.; Kinzel, B.; Kolb, F. A.; Barbieri, S.; Wagner, J.; Durand, C.; Fettis, K.; Dussauge, S.; Hughes, N.; Delgado, O.; Hommel, U.; Gould, T.; Sweeney, A. Mac; Gerhartz, B.; Cumin, F.; Flohr, S.; Schubart, A.; Jaffee, B.; Harrison, R.; Risitano, A. M.; Eder, J.; Anderson, K. Small-Molecule Factor D Inhibitors Targeting the Alternative Complement Pathway. *Nature Chemical*

Biology 2016 12:12 **2016**, *12* (12), 1105–1110. https://doi.org/10.1038/nchembio.2208.

(3) O'Boyle, N. M.; Banck, M.; James, C. A.; Morley, C.; Vandermeersch, T.; Hutchison, G. R. Open Babel: An Open Chemical Toolbox. *J. Cheminform.* **2011**, *3* (1), 33. https://doi.org/10.1186/1758-2946-3-33.

(4) Zdrazil, B.; Felix, E.; Hunter, F.; Manners, E. J.; Blackshaw, J.; Corbett, S.; de Veij, M.; Ioannidis, H.; Lopez, D. M.; Mosquera, J. F.; Magarinos, M. P.; Bosc, N.; Arcila, R.; Kizilören, T.; Gaulton, A.; Bento, A. P.; Adasme, M. F.; Monecke, P.; Landrum, G. A.; Leach, A. R. The ChEMBL Database in 2023: A Drug Discovery Platform Spanning Multiple Bioactivity Data Types and Time Periods. *Nucleic Acids Res.* **2024**, *52* (D1), D1180–D1192. https://doi.org/10.1093/NAR/GKAD1004.

(5) Stein, R. M.; Yang, Y.; Balius, T. E.; O'Meara, M. J.; Lyu, J.; Young, J.; Tang, K.; Shoichet, B. K.; Irwin, J. J. Property-Unmatched Decoys in Docking Benchmarks. *J. Chem. Inf. Model.* **2021**, *61* (2), 699–714. https://doi.org/10.1021/ACS.JCIM.0C00598.

(6) Robin, X.; Turck, N.; Hainard, A.; Tiberti, N.; Lisacek, F.; Sanchez, J.-C.; Müller, M. PROC: An Open-Source Package for R and S+ to Analyze and Compare ROC Curves. *BMC Bioinformatics* **2011**, *12* (1), 77. https://doi.org/10.1186/1471-2105-12-77.

(7) Yabuuchi H. Enrichvis, Version 0.05. The Comprehensive R Archive Network 2011. Https://Cran.r-Project.Org/Web/Packages/Enrichvs/.

(8) Wang, J.; Wolf, R. M.; Caldwell, J. W.; Kollman, P. A.; Case, D. A. Development and Testing of a General Amber Force Field. *J. Comput. Chem.* **2004**, *25* (9), 1157–1174. https://doi.org/10.1002/jcc.20035.

(9) Jakalian, A.; Bush, B. L.; Jack, D. B.; Bayly, C. I. Fast, Efficient Generation of High-Quality Atomic Charges. AM1-BCC Model: I. Method. *J. Comput. Chem.* **2000**, *21* (2), 132–146. https://doi.org/10.1002/(SICI)1096-987X(20000130)21:2<132::AID-JCC5>3.0.CO;2-P.

(10) Berendsen, H. J. C.; Postma, J. P. M.; Van Gunsteren, W. F.; Dinola, A.; Haak, J. R. Molecular Dynamics with Coupling to an External Bath. *J. Chem. Phys.* **1984**, *81* (8), 3684–3690. https://doi.org/10.1063/1.448118.

(11) Pastor, R. W.; Brooks, B. R.; Szabo, A. An Analysis of the Accuracy of Langevin and Molecular Dynamics Algorithms. *Mol. Phys.* **1988**, *65* (6), 1409–1419. https://doi.org/10.1080/00268978800101881.

(12) Darden, T.; York, D.; Pedersen, L. Particle Mesh Ewald: An N·log(N) Method for Ewald Sums in Large Systems. *J. Chem. Phys.* **1993**, *98* (12), 10089–10092. https://doi.org/10.1063/1.464397.

(13) Ryckaert, J. P.; Ciccotti, G.; Berendsen, H. J. C. Numerical Integration of the Cartesian Equations of Motion of a System with Constraints: Molecular Dynamics of n-Alkanes. *J. Comput. Phys.* **1977**, *23* (3), 327–341. https://doi.org/10.1016/0021-9991(77)90098-5.

(14) Forouzesh, N.; Izadi, S.; Onufriev, A. V. Grid-Based Surface Generalized Born Model for Calculation of Electrostatic Binding Free Energies. *J. Chem. Inf. Model.* **2017**, *57* (10), 2505–2513. https://doi.org/10.1021/acs.jcim.7b00192.

Supplementary Tables

Table S1. Structure, percentage of inhibition ($I_{\%}$), IC_{50} and ligand efficiency (LE) of the fragments selected against KLK6.

**Compound**	**Structure**	**KLK5**	**KLK6**	**KLK7**	**KLK8**
		$I_{\%}$<br>**IC_{50}**<br>*LE*	$I_{\%}$<br>**IC_{50}**<br>*LE*	$I_{\%}$<br>**IC_{50}**<br>*LE*	$I_{\%}$<br>**IC_{50}**<br>*LE*
1	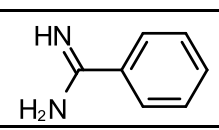	22	41	11	1
2	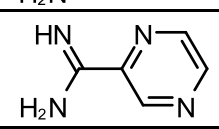	2	14	1	3
3	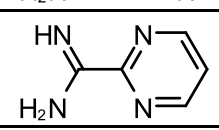	9	3	2	0
4	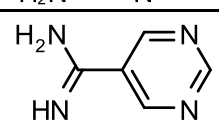	0	7	0	0
5	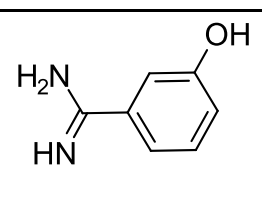	19	35	6	0
6	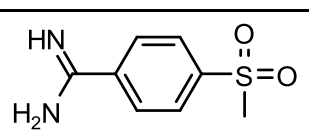	3	3	5	0

7	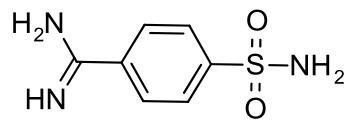	1	14	8	4
8	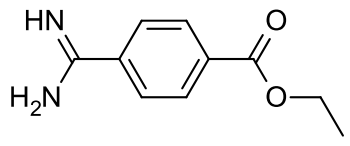	7	18	0	0
9	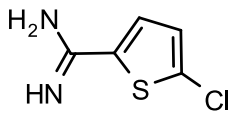	35	80<br>**26 ± 5**<br>*0.70*	1	23
10	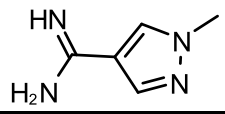	7	13	0	0
11	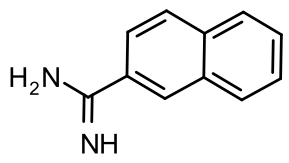	45	79<br>**52 ± 6**<br>*0.45*	8	35
12	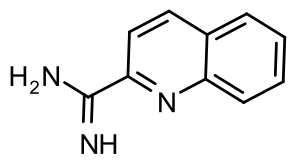	37	63<br>**32.7 ± 1.6**<br>*0.47*	3	17
13	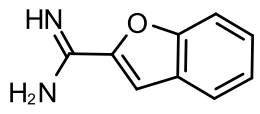	22	68<br>**37.2 ± 1.7**<br>*0.51*	1	12
14	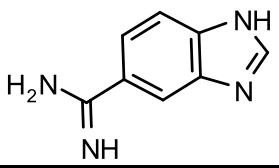	15	19	4	3
15	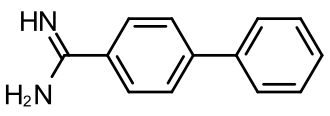	23	85<br>**20 ± 2**<br>*0.43*	15	15
16	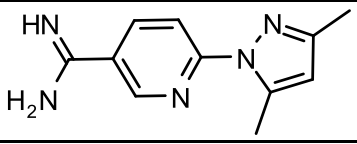	1	10	0	0
17	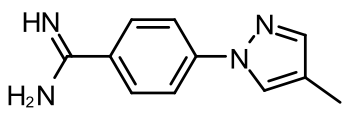	15	56<br>**91.3 ± 2.8**<br>*0.37*	6	2
18	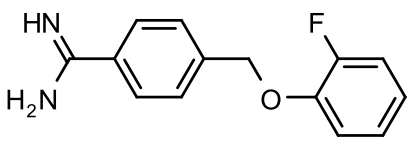	45	93<br>**26 ± 5**<br>*0.35*	10	17
19	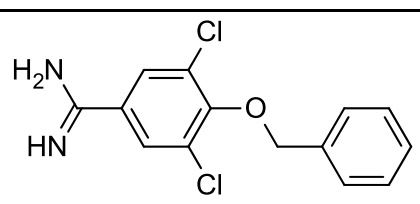	44	82<br>**12 ± 1**<br>*0.35*	7	25
20	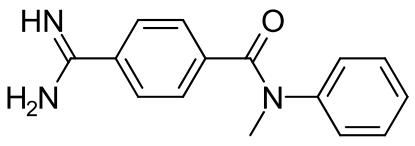	4	6	0	0
21	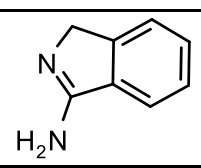	34	36	13	3
22	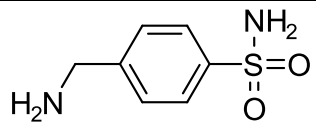	13	0	2	4
23	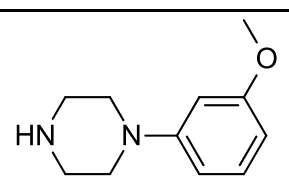	2	11	5	8
24	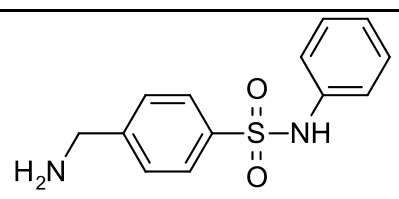	3	7	13	6
25	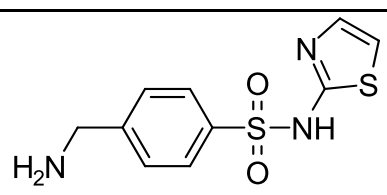	5	10	15	4
26	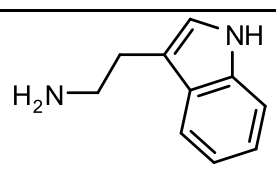	0	2	8	0
27	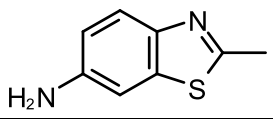	0	11	2	5

28	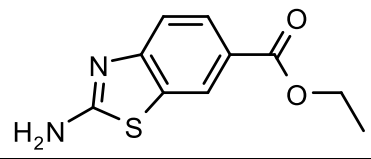	12	9	31	9
29	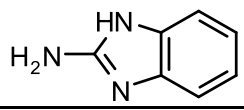	11	20	0	4
30	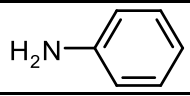	0	0	1	0
31	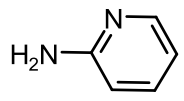	0	0	0	1
32	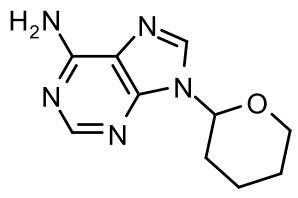	1	5	4	0
33	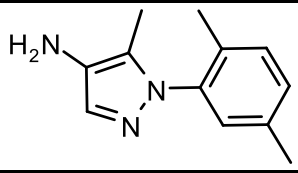	8	2	3	0
34	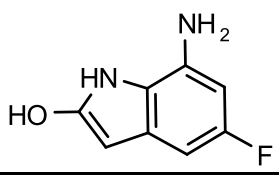	2	4	17	0
35	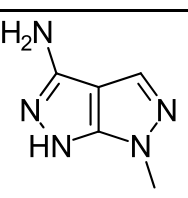	2	0	0	6
36	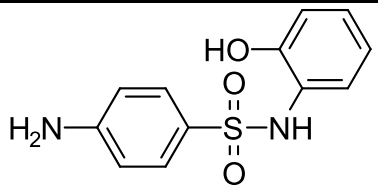	9	0	17	25
37	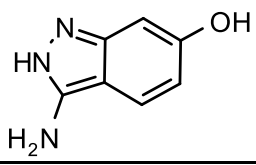	7	8	9	0

Table S2. Structure, percentage of inhibition ($I_{\%}$), IC_{50} and ligand efficiency (LE) of the fragments selected against KLK7.

**Compound**	**Structure**	**KLK5**	**KLK6**	**KLK7**	**KLK8**
		$I_{\%}$<br>**IC_{50}**<br>*LE*	$I_{\%}$<br>**IC_{50}**<br>*LE*	$I_{\%}$<br>**IC_{50}**<br>*LE*	$I_{\%}$<br>**IC_{50}**<br>*LE*
38	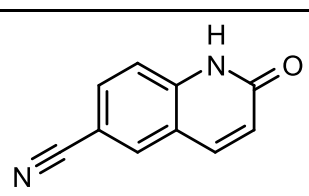	22	**43**	**80**<br>**43.3 ± 2.2**<br>**0.46**	13
39	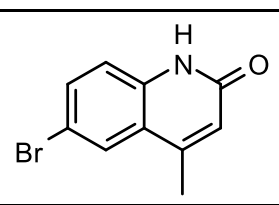	9	14	**44**	0
40	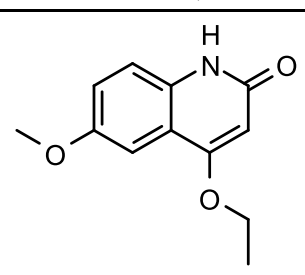	6	4	2	0
41	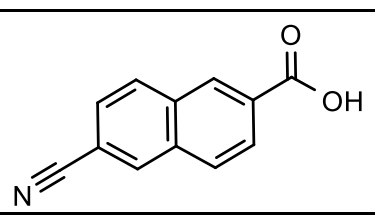	7	6	15	5
42	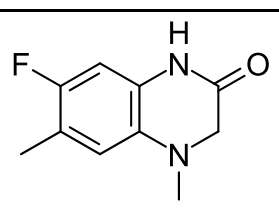	2	2	12	12
43	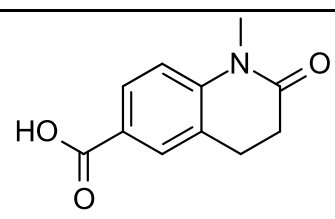	3	5	10	0
44	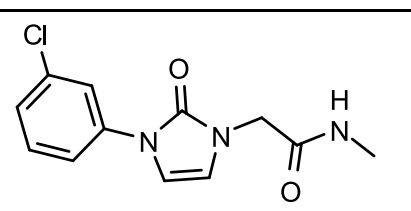	1	1	**54**	0

45	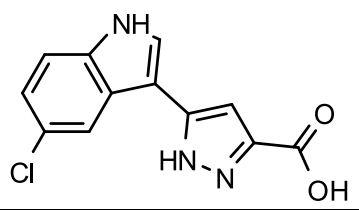	**49**	3	0	24
46	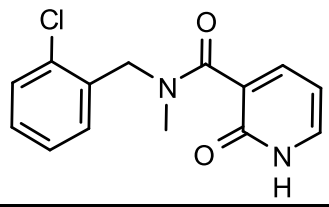	0	0	16	0
47	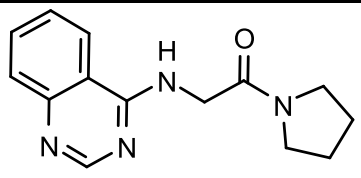	5	8	14	1
48	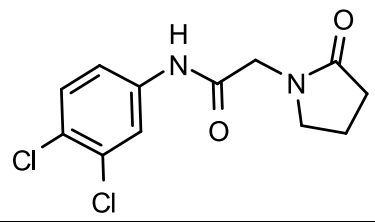	0	0	18	0
49	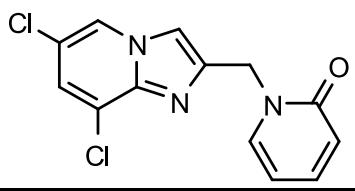	0	2	13	11
50	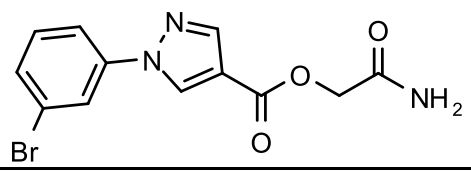	0	4	3	4
51	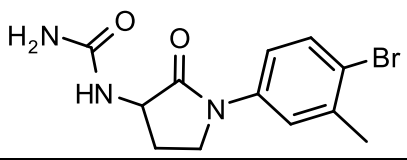	0	0	9	3
52	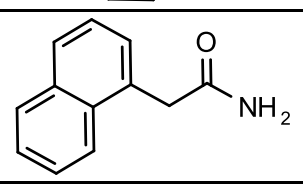	1	3	16	0
53	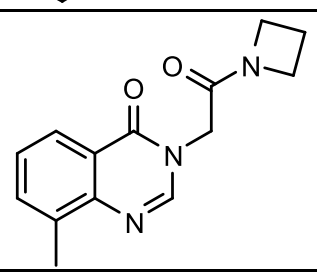	2	0	4	0
54	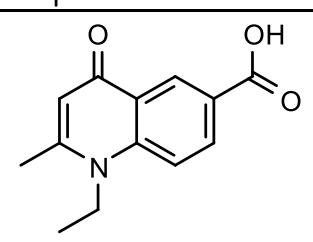	5	5	4	3
55	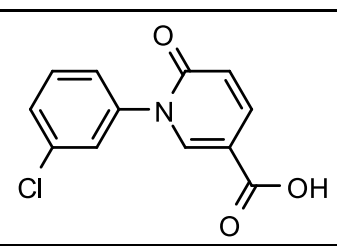	4	3	15	7
56	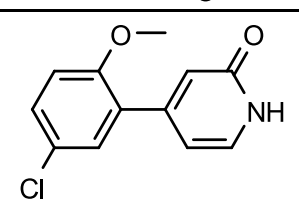	0	2	15	5
57	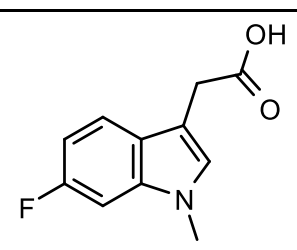	2	4	13	5
58	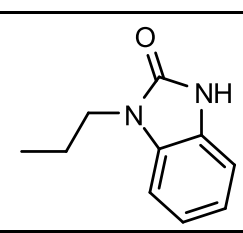	2	4	8	0
59	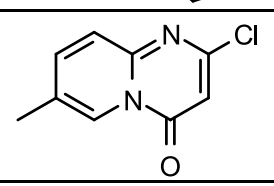	5	7	9	0
60	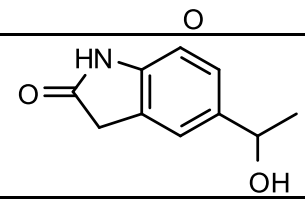	8	9	7	7
61	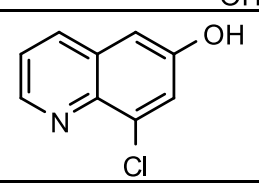	5	11	18	14

Table S3. K_M values determined for the enzyme-substrate pairs under the assay conditions described in the Materials and Methods section.

Enzyme	Substrate	K_M (µM)
KLK1	H-PFR-AMC	10.0
KLK4	Boc-VPR-AMC	4.3
KLK5	Boc-QAR-AMC	52.0
KLK6	Boc-QAR-AMC	150.0
KLK7	MeO-Suc-RPY-AMC	38.0
KLK8	Boc-VPR-AMC	22.6
KLK13	Boc-VPR-AMC	6.7
KLK14	Boc-QAR-AMC	110.0
Trypsin 3	Boc-QAR-AMC	3.2
Plasmin	Boc-VLK-AMC	50.0
Matriptase	Boc-QAR-AMC	15.0

Supplementary Figures

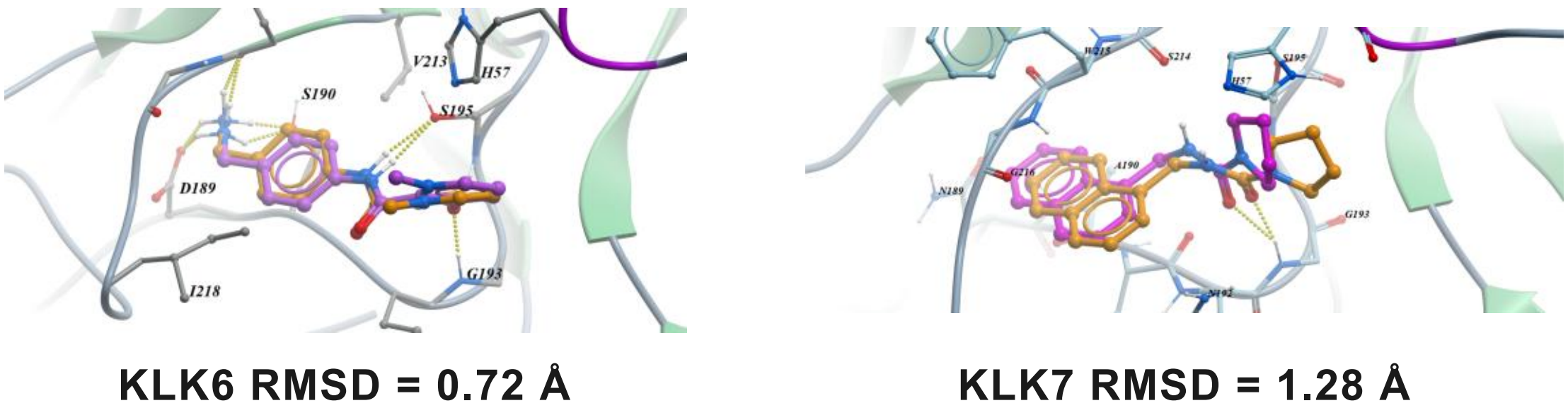

Figure S1. Top-ranked poses from redocking calculations of KLK6 (4D8N) and KLK7 (5FAH). Experimental and docked poses are represented in orange and magenta, respectively. Hydrogen bonding interactions are depicted as yellow dash lines.

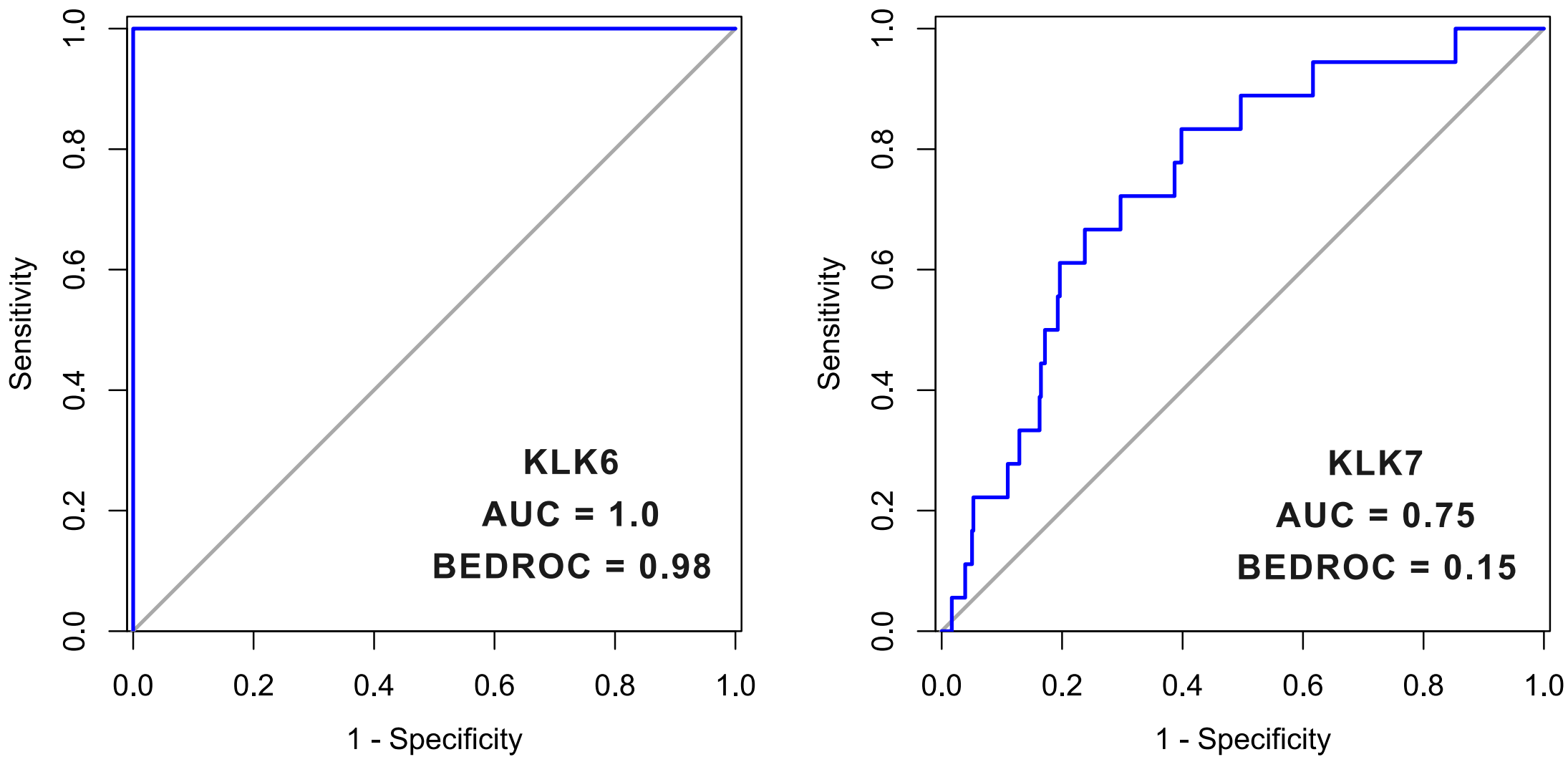

Figure S2. ROC curves (blue) for KLK6 and KLK7. The diagonal line (grey) represents a random-classification performance.

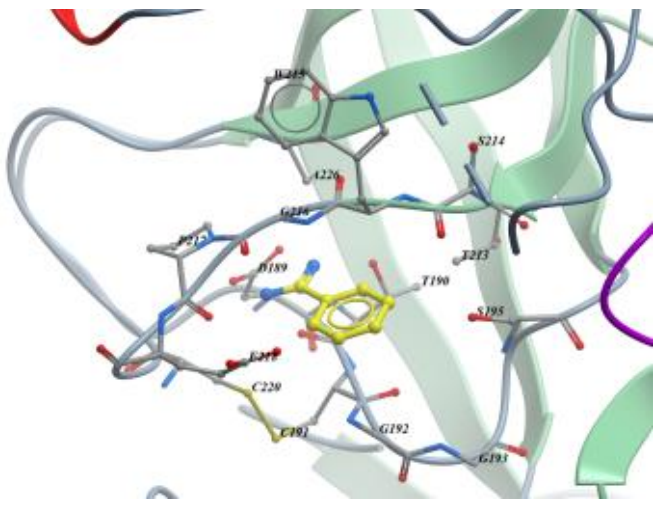

Kallikrein 2
(PDB ID: 4NFE)

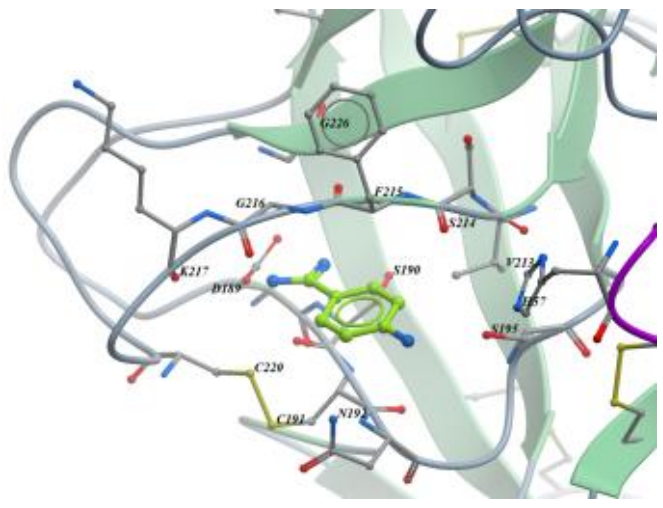

Kallikrein 4
(PDB ID: 2BDH)

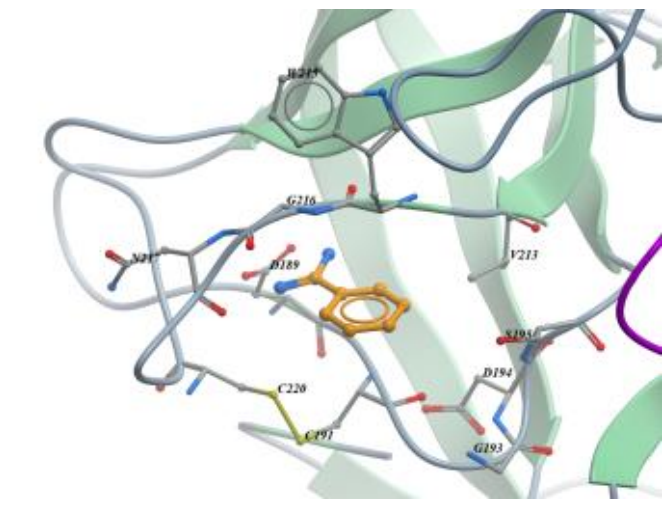

Kallikrein 6
(PDB ID: 1L2E)

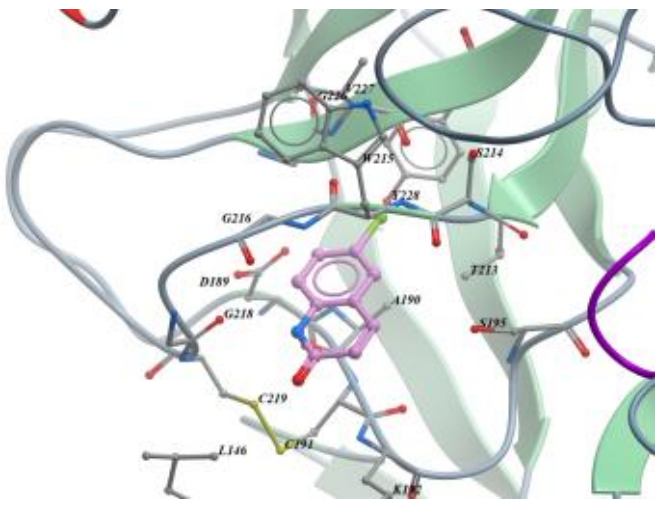

Factor XIa
(PDB ID: 4CR5)

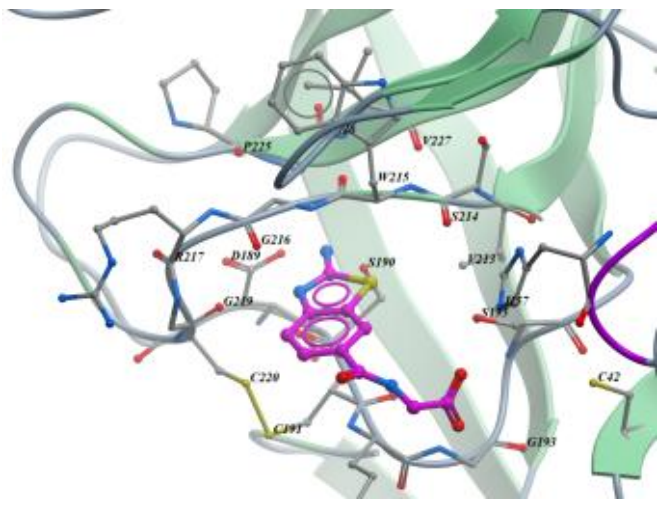

Urokinase
(PDB ID: 4H42)

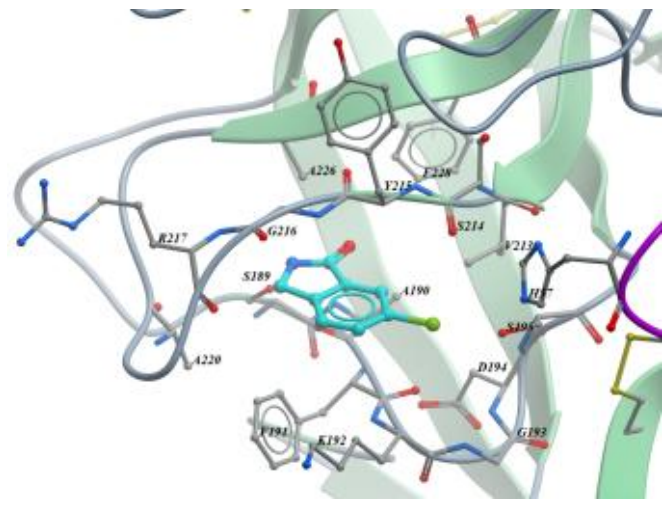

Chymase
(PDB ID: 4K5Z)

Figure S3. Representative structures that contain a trypsin-like serine protease domain (IPR001254). This figure shows that all fragments bind at the S1 pocket of the enzymes.

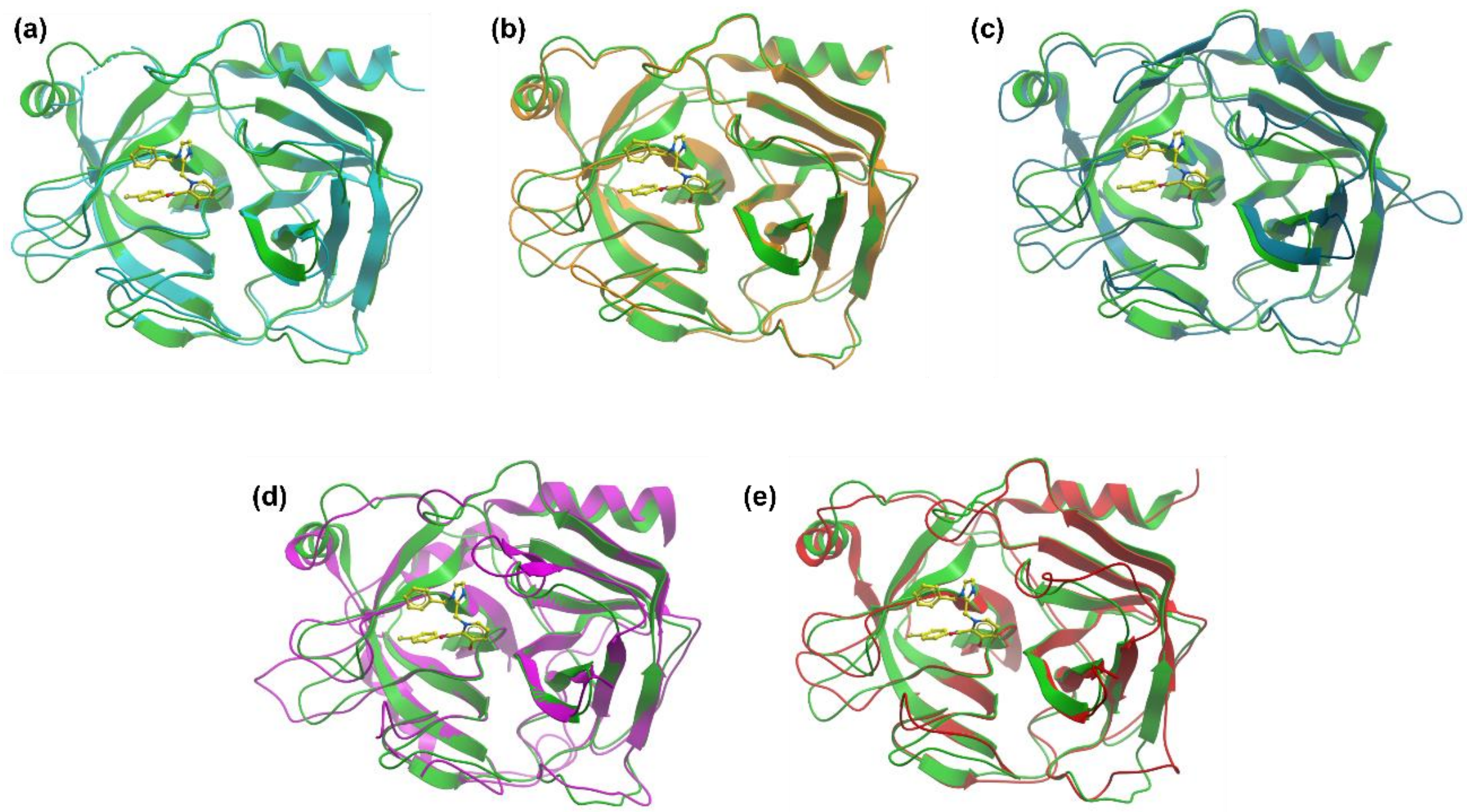

Figure S4. Structural alignment of KLK6 (green, PDB ID: 4D8N) and representative serine proteases: a) KLK7 (cyan, PDB ID: 5FAH); b) trypsin (orange, PDB ID: 4I8H); c) urokinase (blue, PDB ID:5YC6); d) thrombin (magenta, PDB ID: 4UEH); e) factor XIa (red, PDB ID: 4CR9). The ligand in complex with KLK6 is displayed as ball and sticks with yellow carbons.

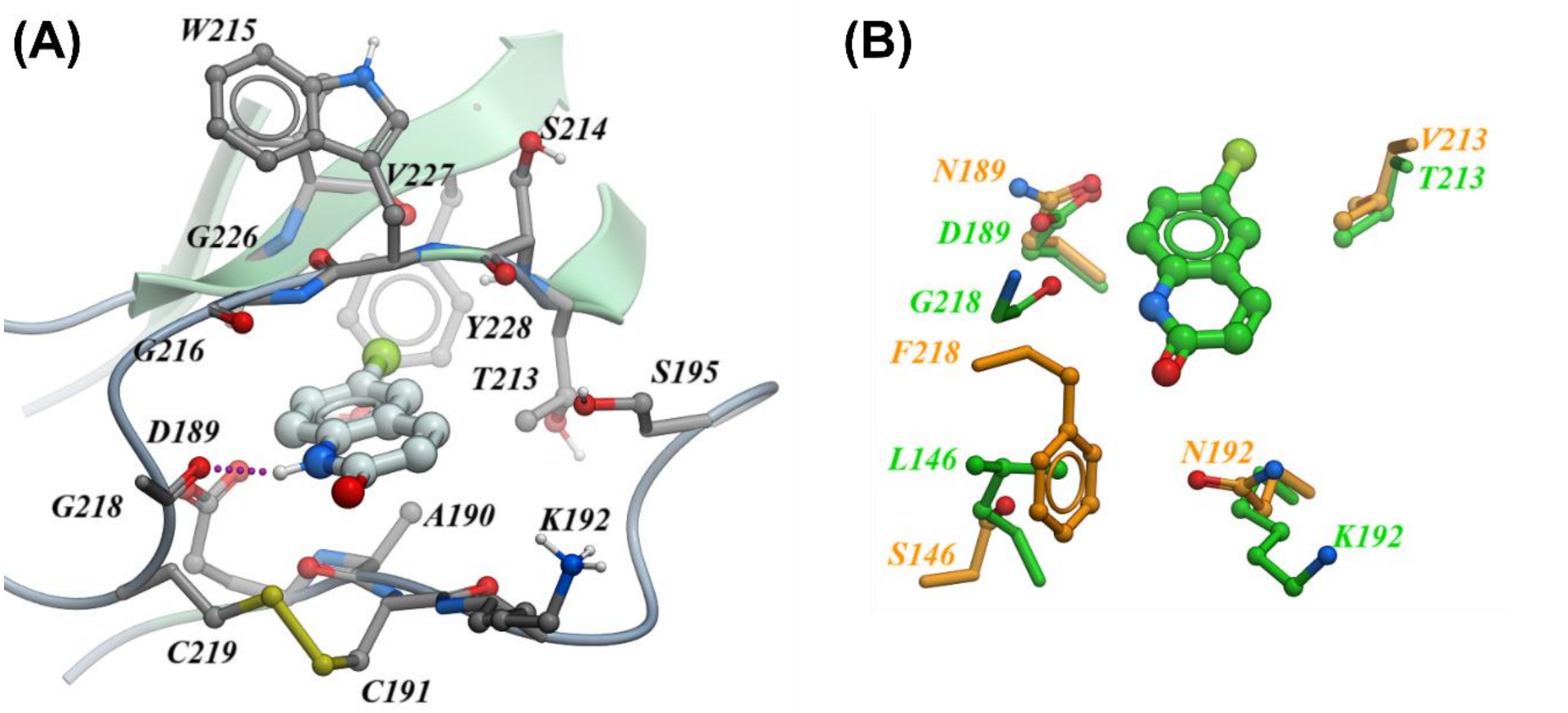

Figure S5. A) Experimental binding pose of the 2-hydroxyquinoline fragment in complex with FXIa (PDB ID: 4CR5); B) S1 pocket amino acids that differ between KLK7 (orange) and FXIa (green). The fragment is displayed as ball and sticks.

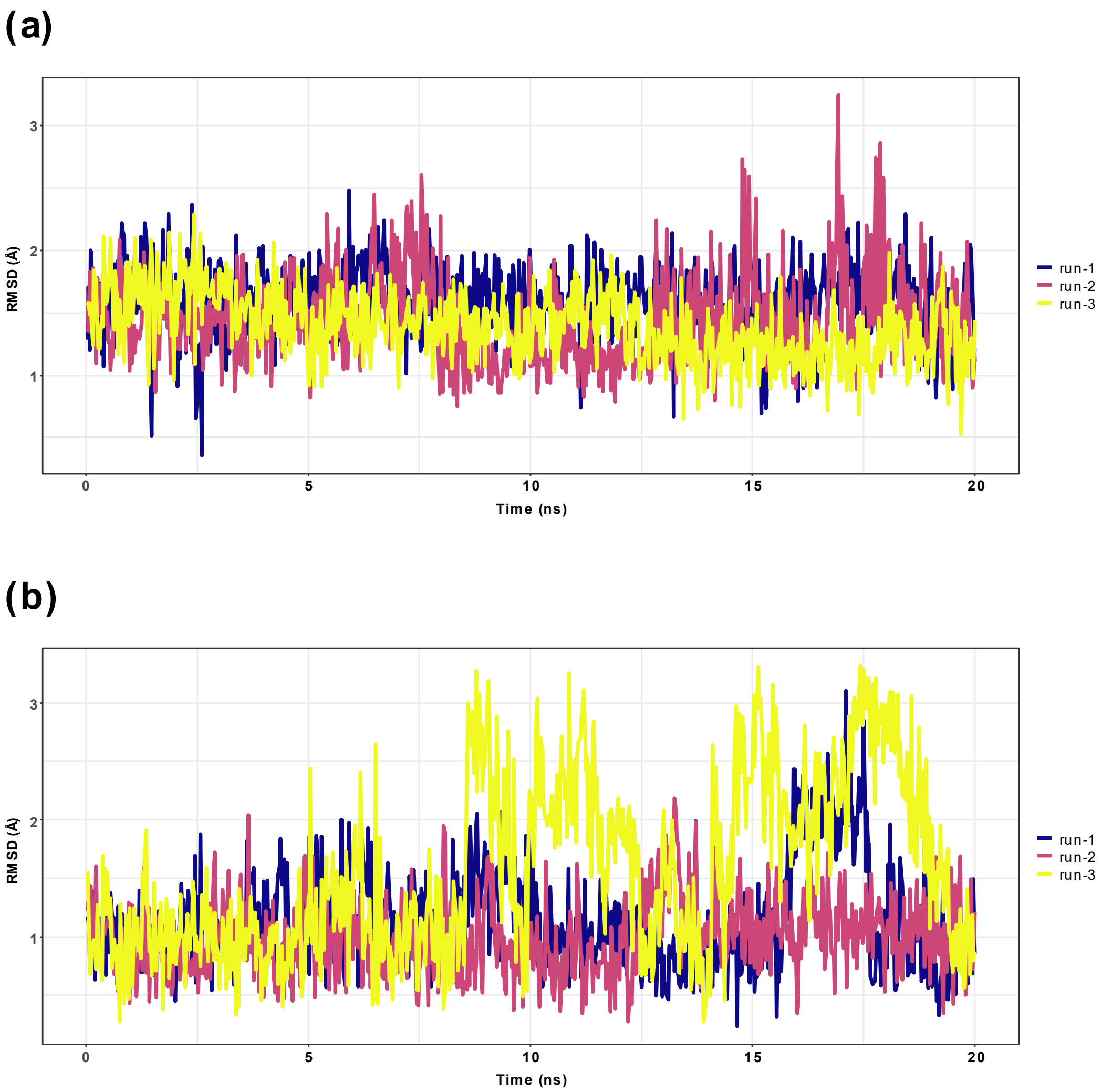

Figure S6. RMSD plot for the MD simulation of **38** in complex with KLK7 (5FAH): (a) pose 1; (b) pose 2.

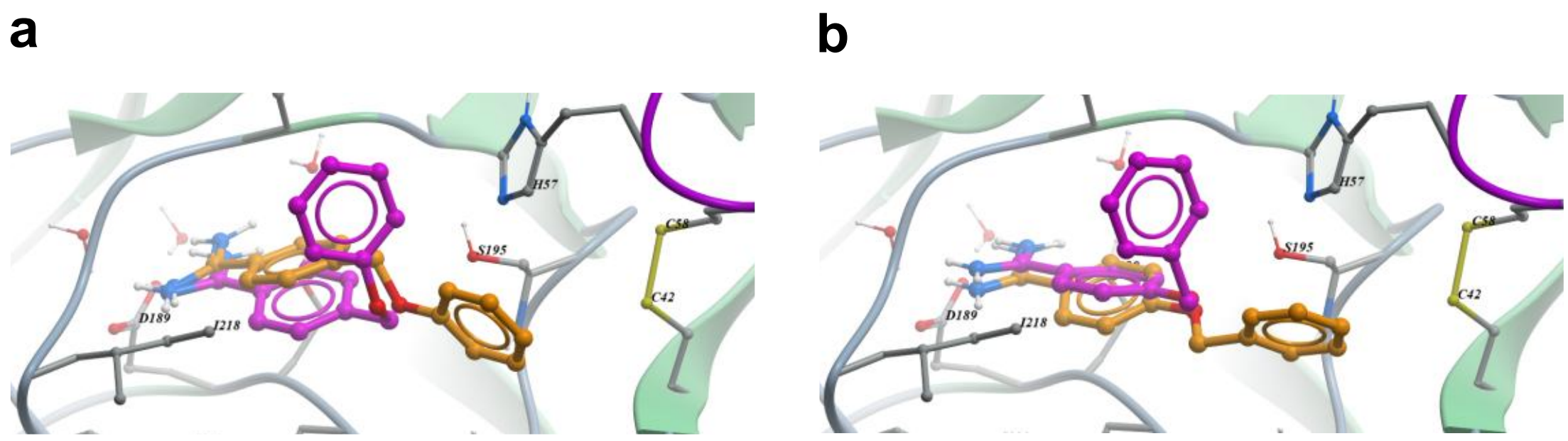

Figure S7. Representative poses of the two most frequent cluster obtained after an exhaustive docking with rDock for fragment (a) **69** and (b) **70**. The carbon atoms of the representative pose of the main cluster (**69** = 44 poses; **70** = 64 poses) are colored in orange. The carbon atoms of the representative pose of the second main cluster (**69** = 26 poses; **70** = 19 poses) are colored in magenta.

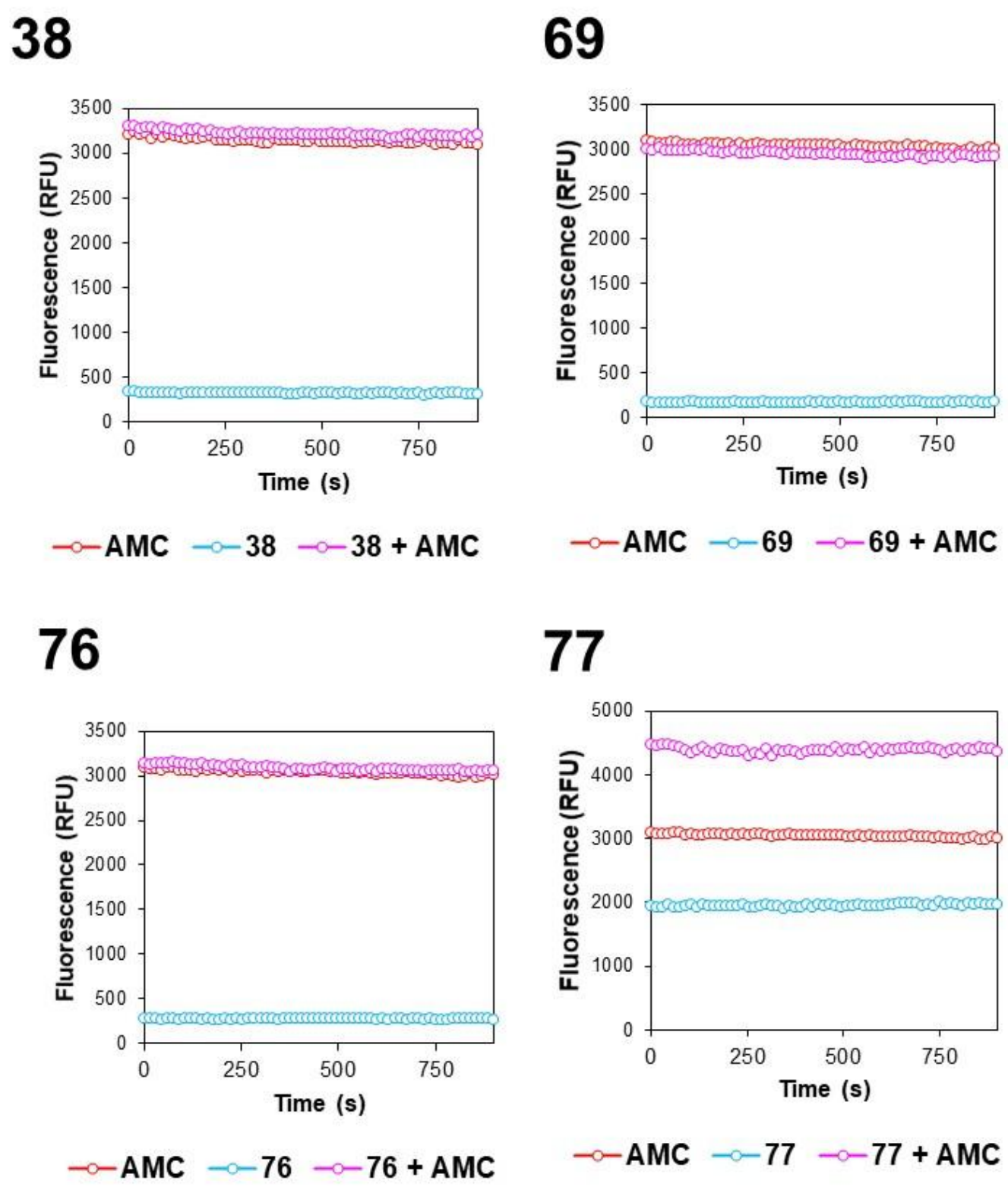

Figure S8. Fluorescence controls for compounds **38**, **69**, **76** and **77** under assay conditions. Fluorescence was monitored over time under assay conditions in the presence of free AMC (200 nM, red), compound alone (100 µM, blue), or compound plus AMC (200 nM AMC + 100 µM compound, purple). Measurements were performed in assay buffer (50 mM Tris, 1 M sodium citrate, 0.05% Brij-35, pH 7.0) in the presence of KLK6. The fluorescence signal measured for AMC remained stable over the course of the experiment and was not significantly altered by the presence of the compounds, indicating negligible quenching, enhancement, or inner-filter effects under the assay conditions used. The AMC concentration used is representative of the product levels generated during the initial-rate measurements.

APENDIX I

HRMS and HPLC spectra of 16

MaxPeak: 100.00%
Ret_Time: 0.638 min

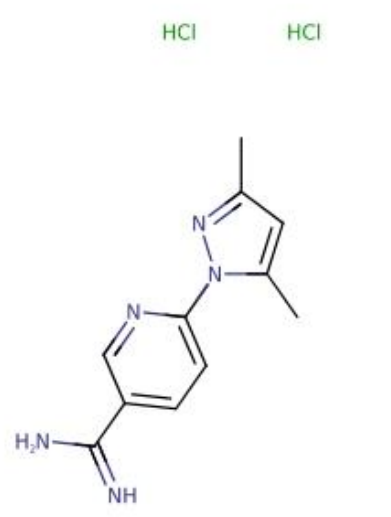

Mol Wt 288.18
Exact Mass 215.13

#	Time	Area%
1	0.638	100.00

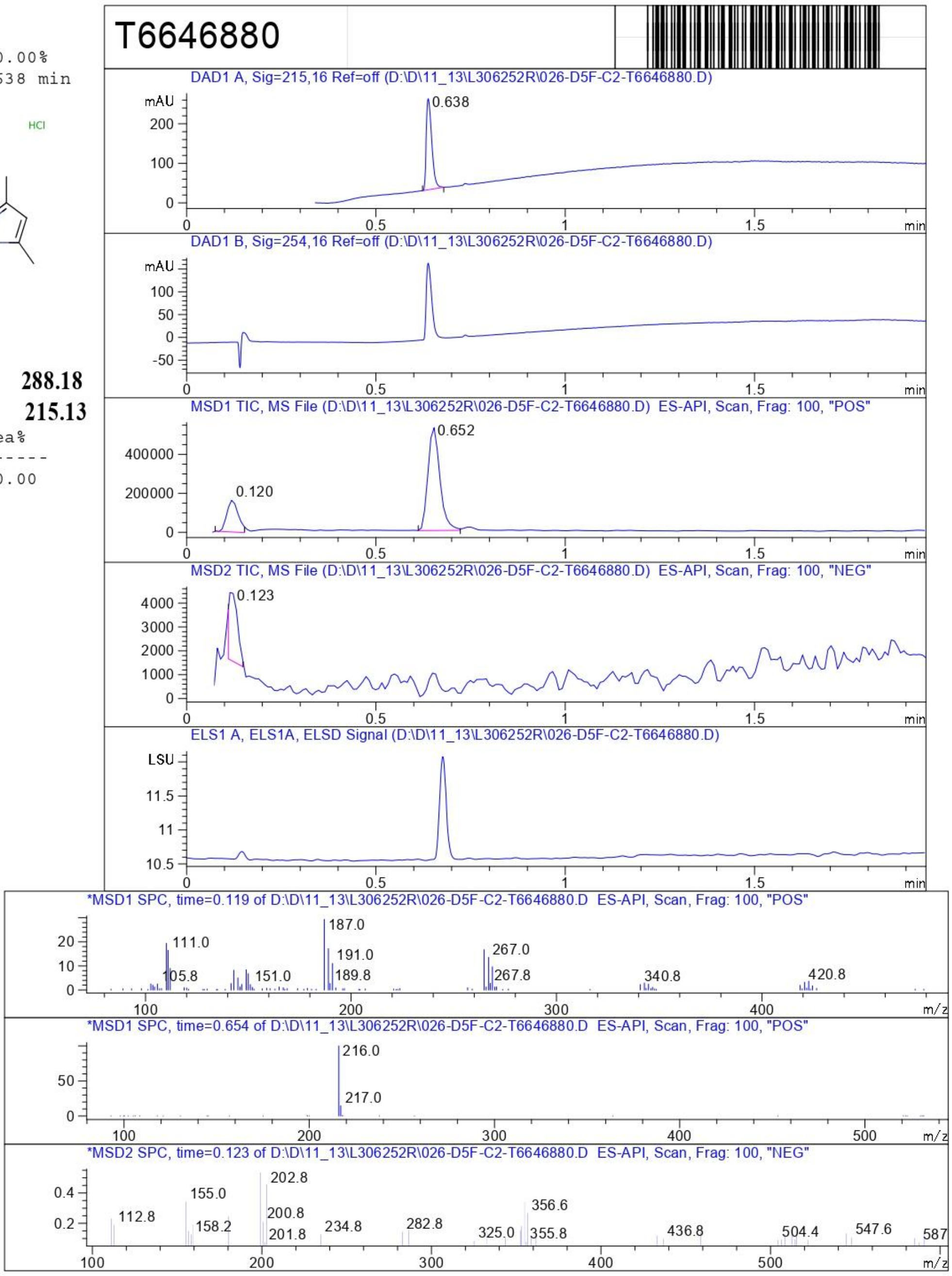

RT 0.120

RT 0.652

RT 0.123

Inj.Date 11/12/2020 N 13 Acq. Method C:\Chem32\-> ->

HRMS and HPLC spectra of 17

MaxPeak: 95.88%
Ret_Time: 0.759 min

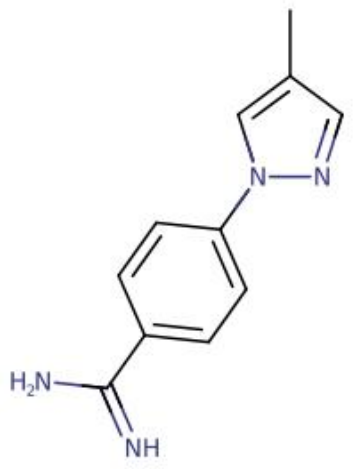

Mol Wt 200.24

Exact Mass 200.12

#	Time	Area%
1	0.759	95.88
2	0.963	4.12

RT 0.769

RT 0.775

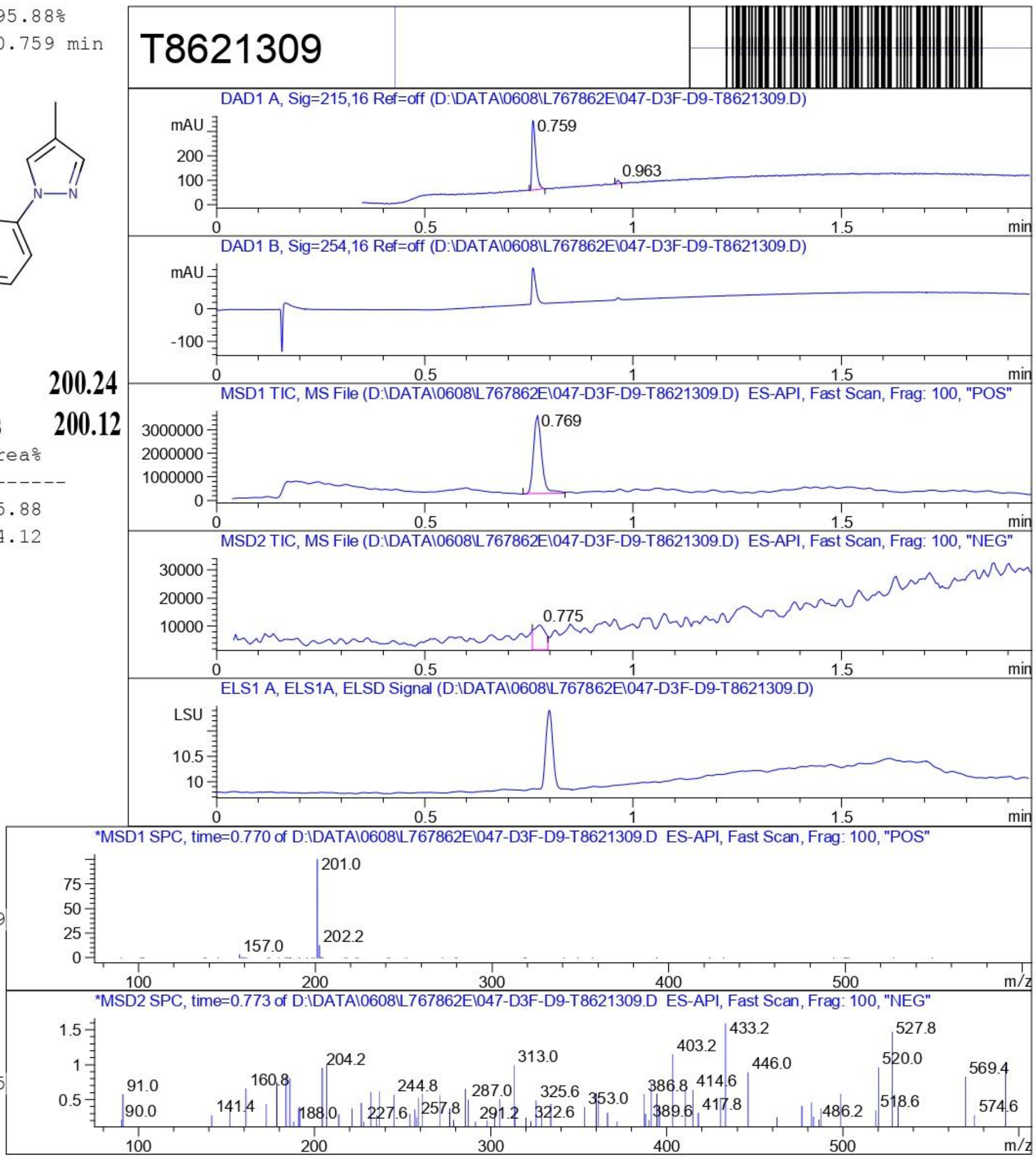

Inj.Date 6/7/2024 29 M
C:\Users\Public\Documents\ChemStation\1\Data\06_07\L767862E\SUPOR_96.M

HRMS and HPLC spectra of 18

MaxPeak: 94.38%
Ret_Time: 0.758 min

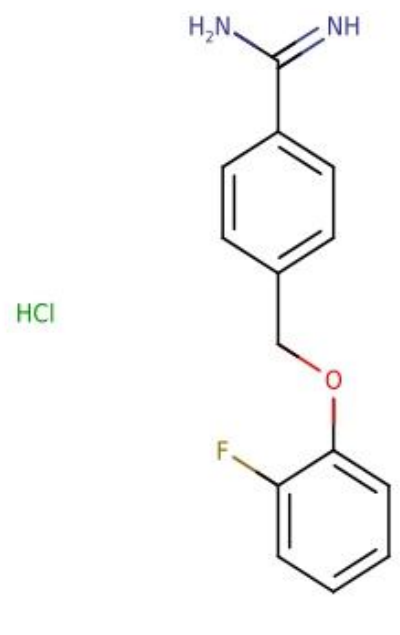

Mol Wt 280.73
Exact Mass 244.12

#	Time	Area%
1	0.758	94.38
2	0.998	5.62

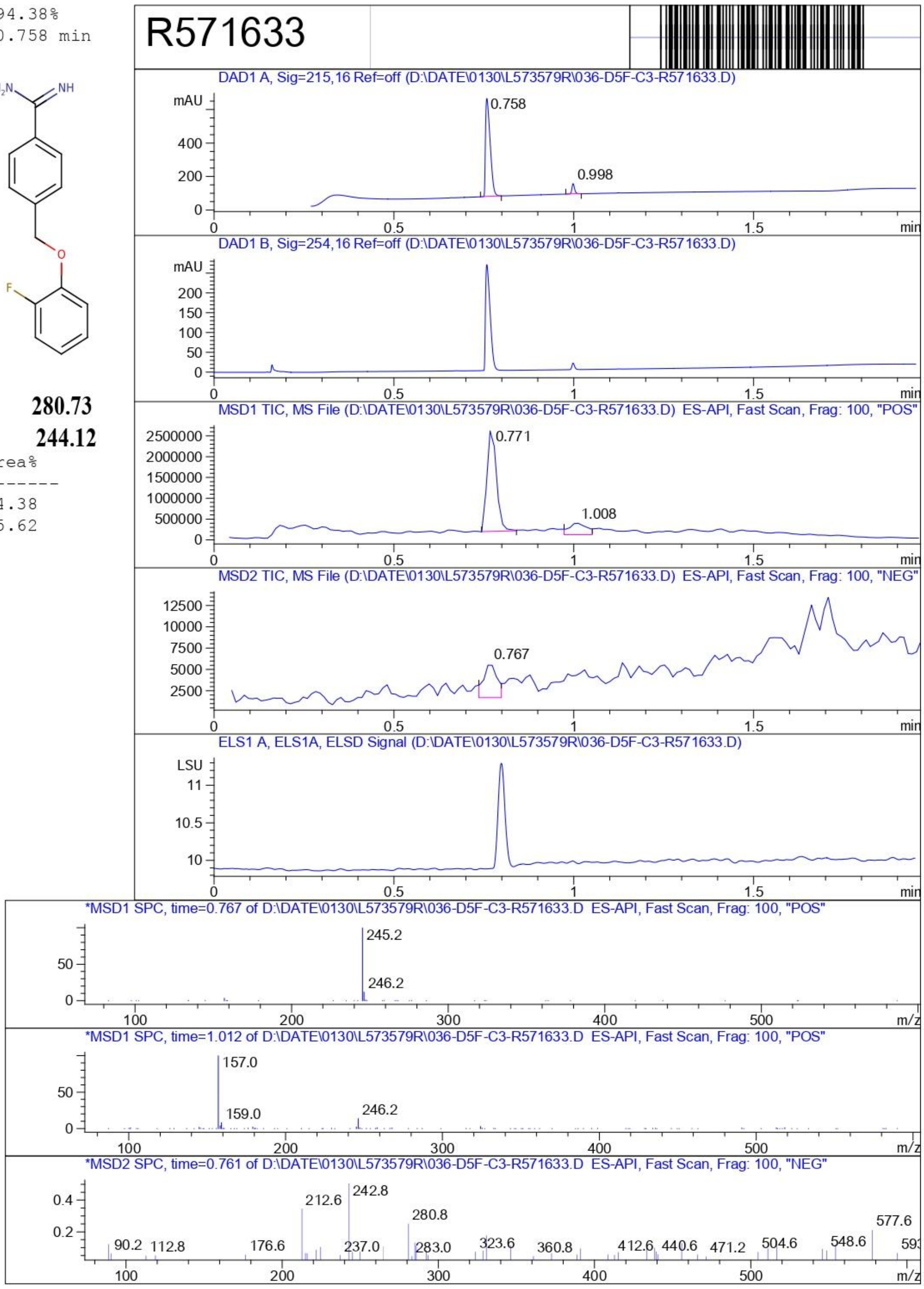

Inj.Date 1/30/2023 E Acq. Method C:\Users\ -> ->

HRMS and HPLC spectra of 62

MaxPeak: 100.00%
Ret_Time: 0.663 min

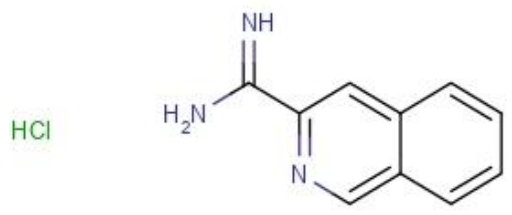

Mol Wt 207.66

Exact Mass 171.09

#	Time	Area%
1	0.663	100.00

RT 0.695

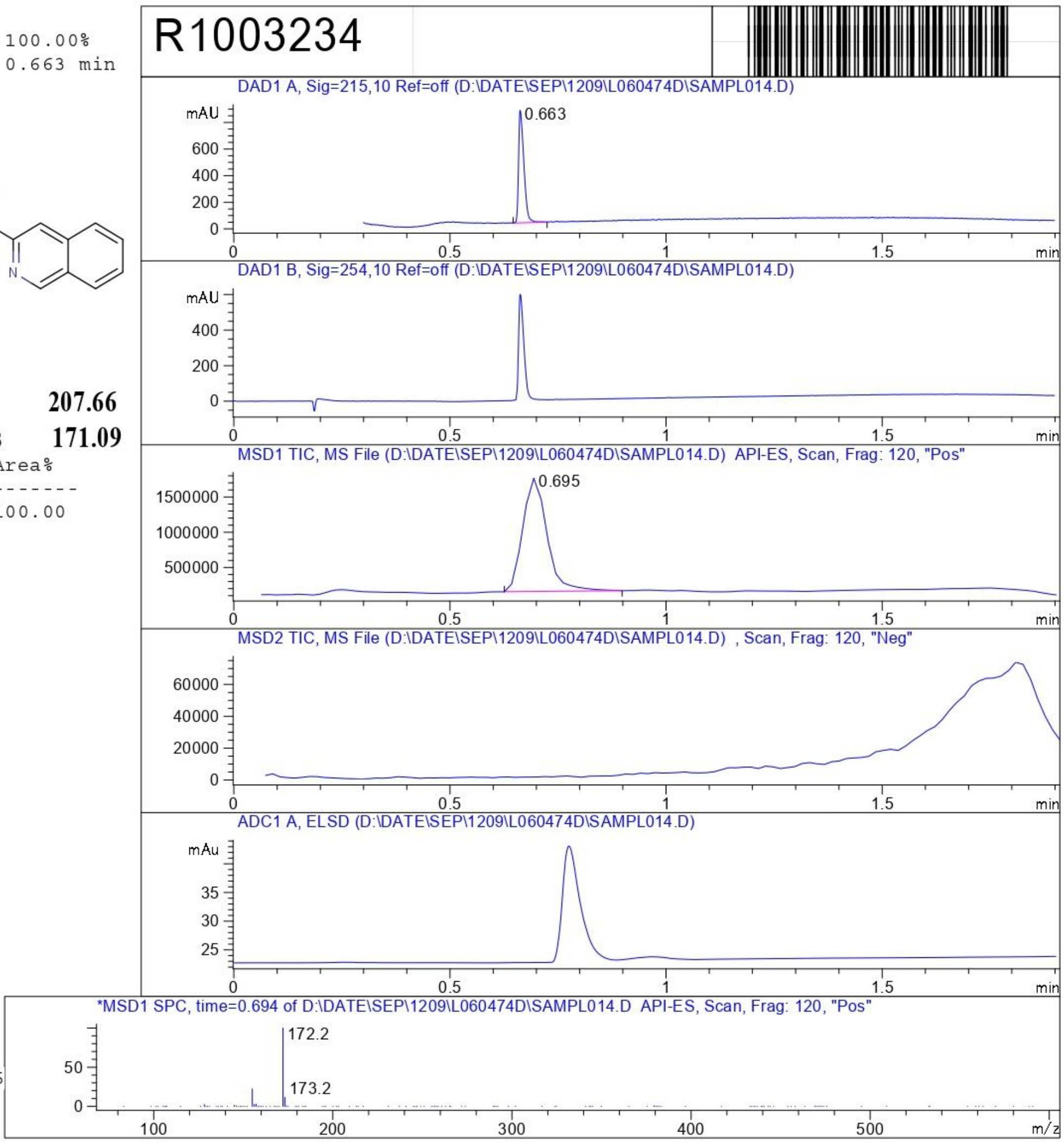

Inj.Date 9/12/2017 A P2-B-05 -SL- Acq. Method C:\HPCHEM\-> ->

HRMS and HPLC spectra of 64

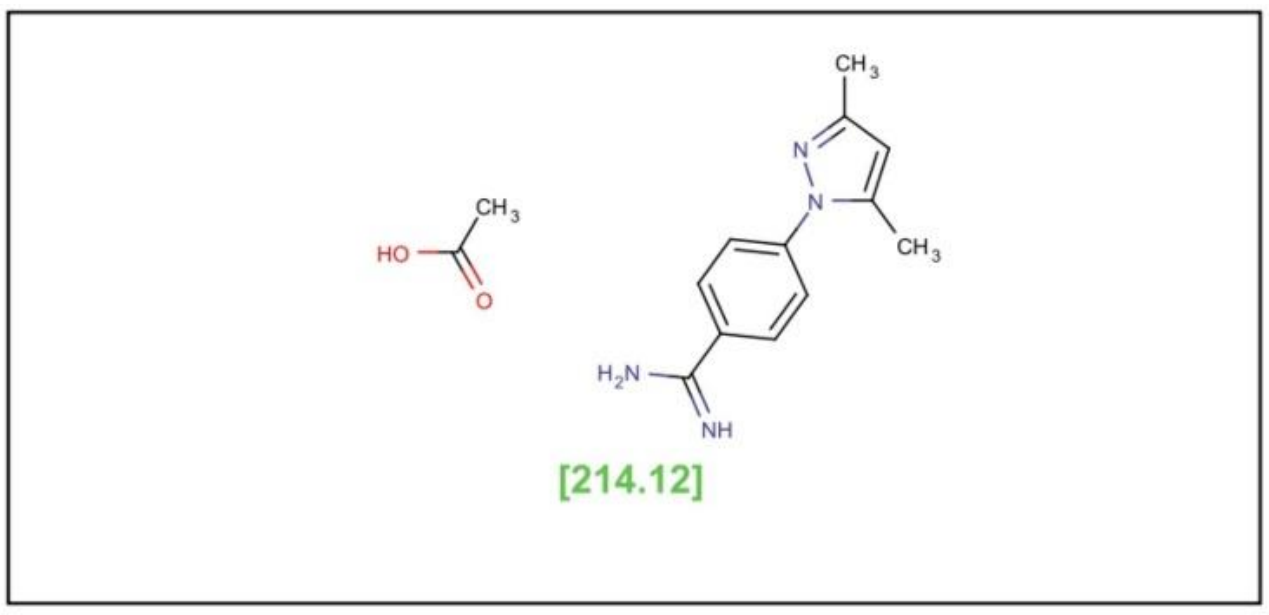

T8624414

L631332R
LCMS-7
SUPOR_96.M
15:54 04.07.2023
MaxPeak: 100.0%

#	RT	DAD1A	DAD1B	MSD1	MSD2	ELSD	MSD1 ions	MSD1 rt	MSD2 ions	MSD2 rt	Info
1	0.744	100.0%	100.0%	100.0%	---	100.0%	215.2(97),198.2(3)	0.752	---	---	P +H+

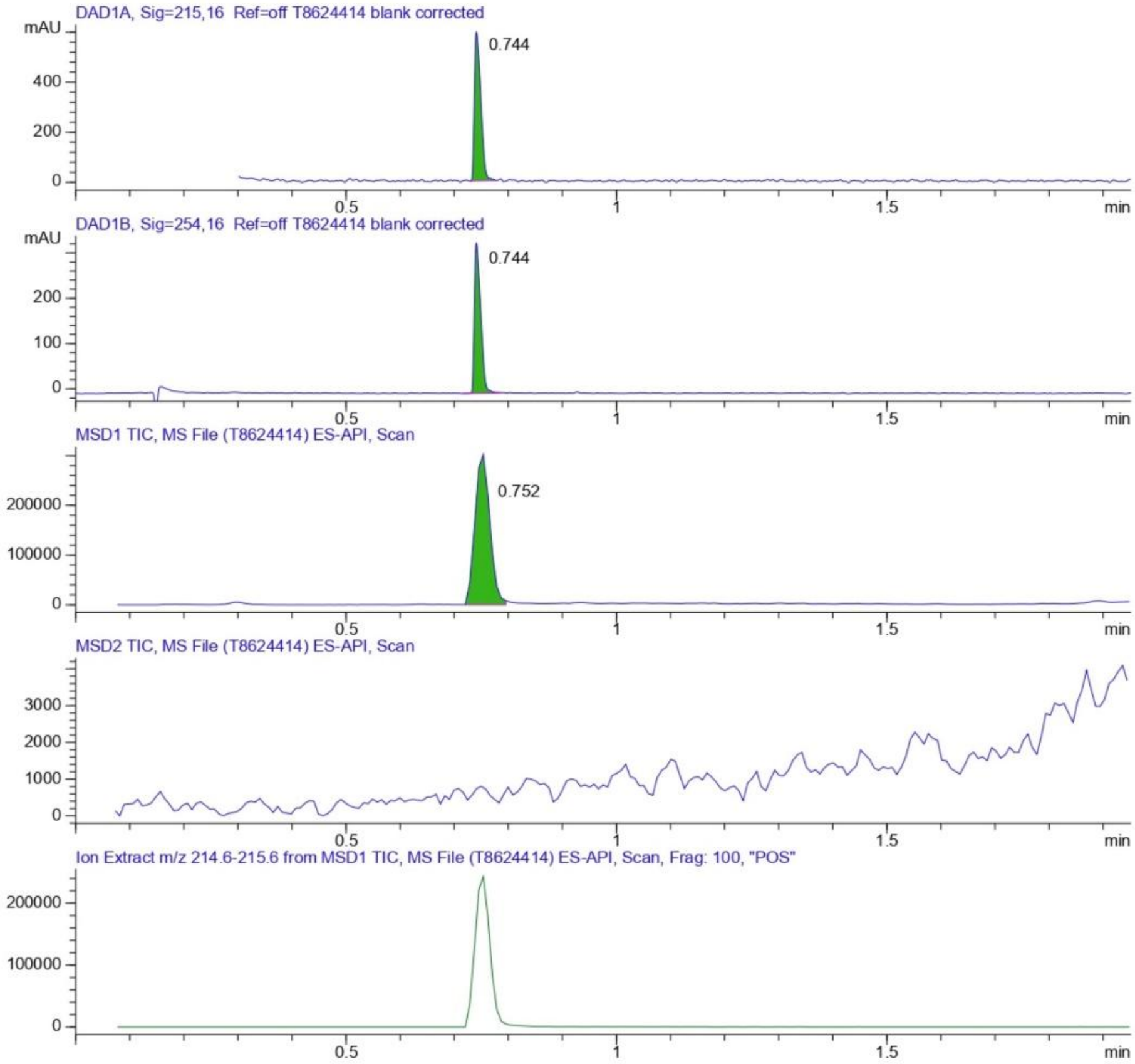

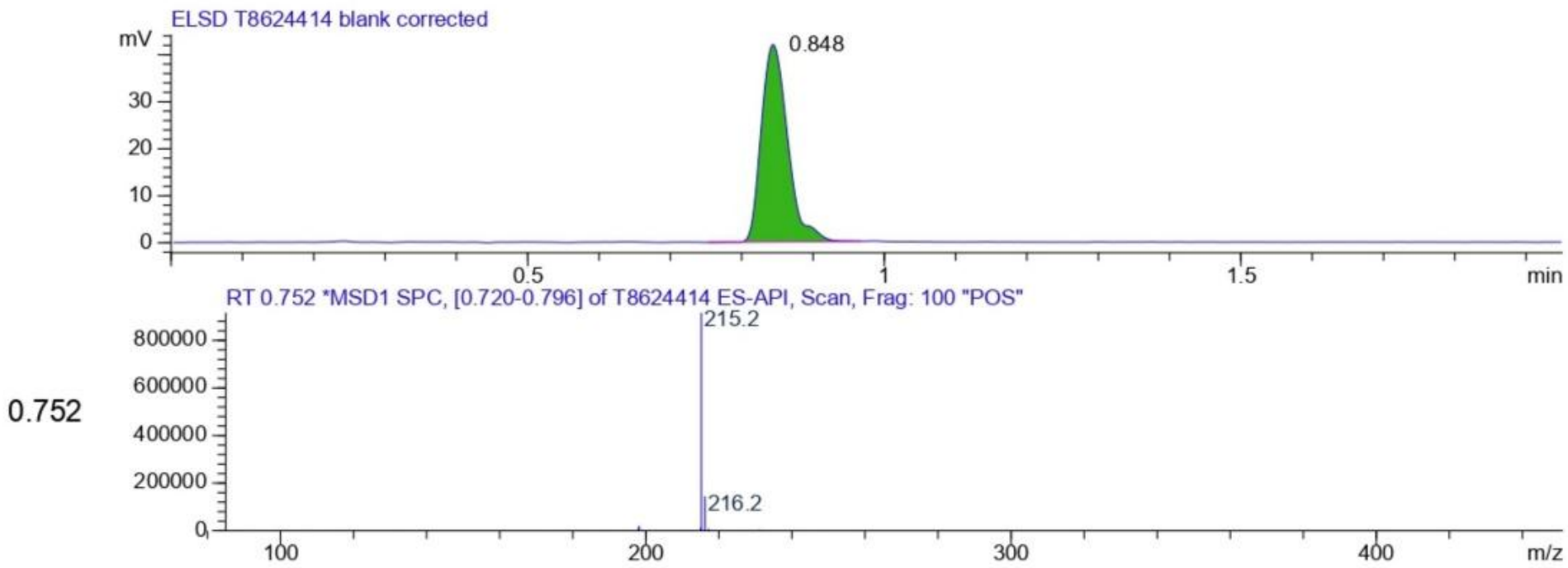

HRMS and HPLC spectra of 65

MaxPeak: 100.00%
Ret_Time: 0.757 min

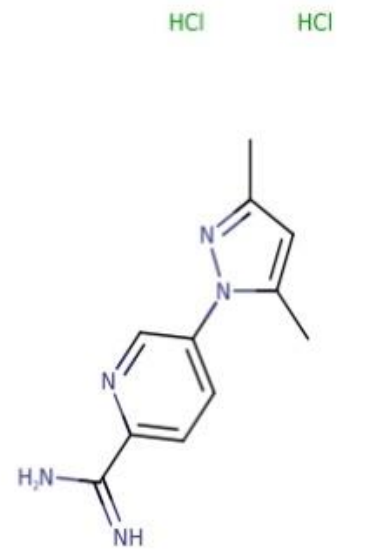

Mol Wt 288.18

Exact Mass 215.13

#	Time	Area%
1	0.757	100.00

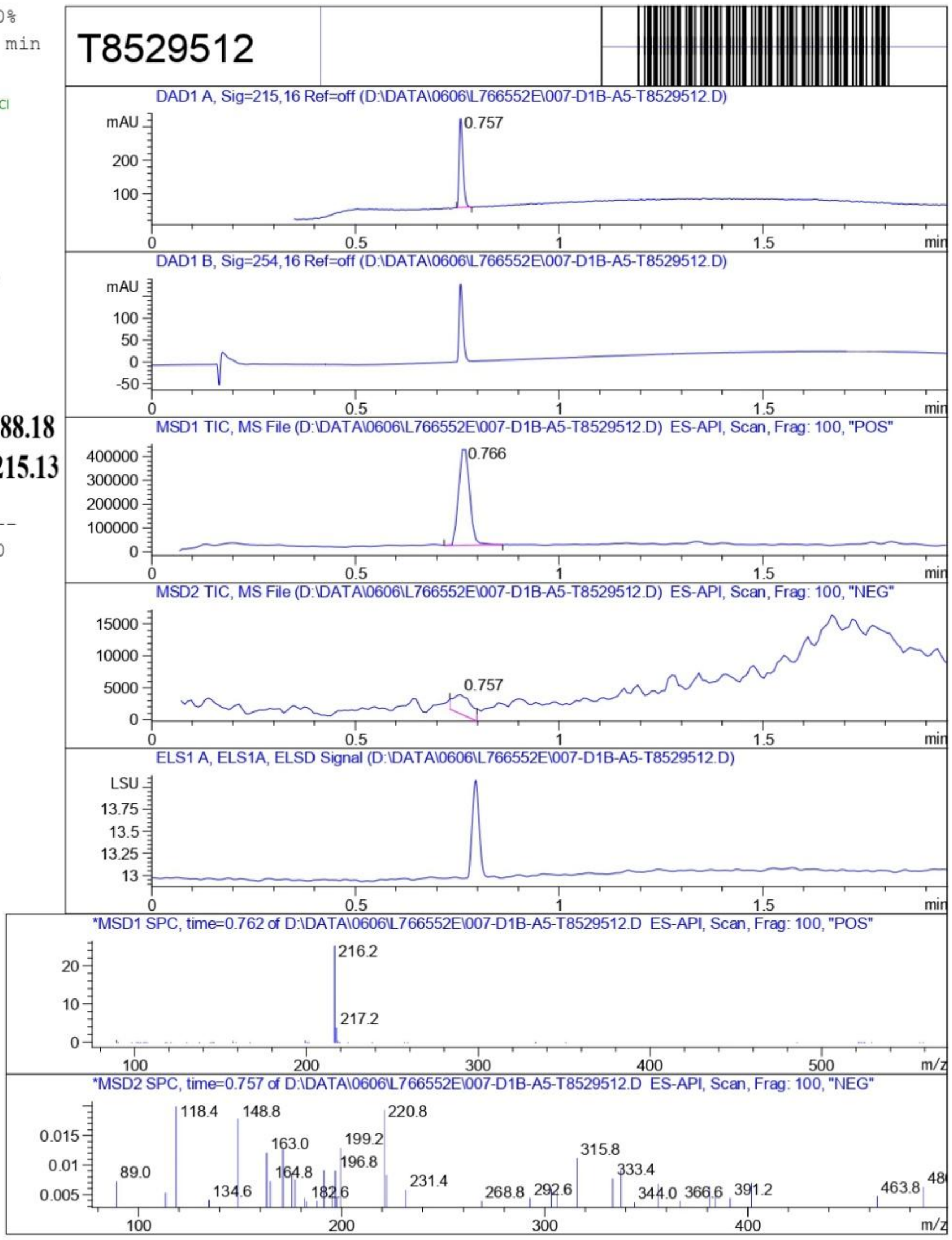

RT 0.766

RT 0.757

Inj.Date 6/6/2024 LB -17-
C:\Chem32\1\Data\06_05\L766552E\SUPOR_96.M

HRMS and HPLC spectra of 66

MaxPeak: 100.00%
Ret_Time: 0.543 min

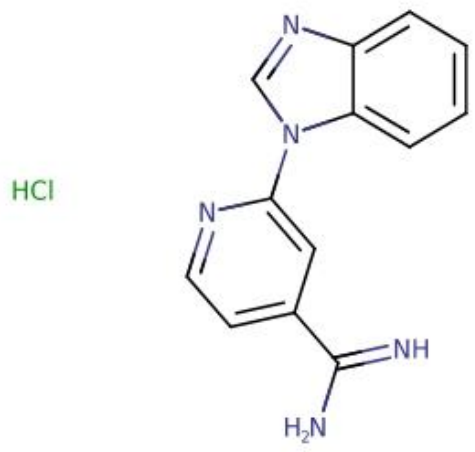

Mol Wt 273.72
Exact Mass 237.11

#	Time	Area%
1	0.543	100.00

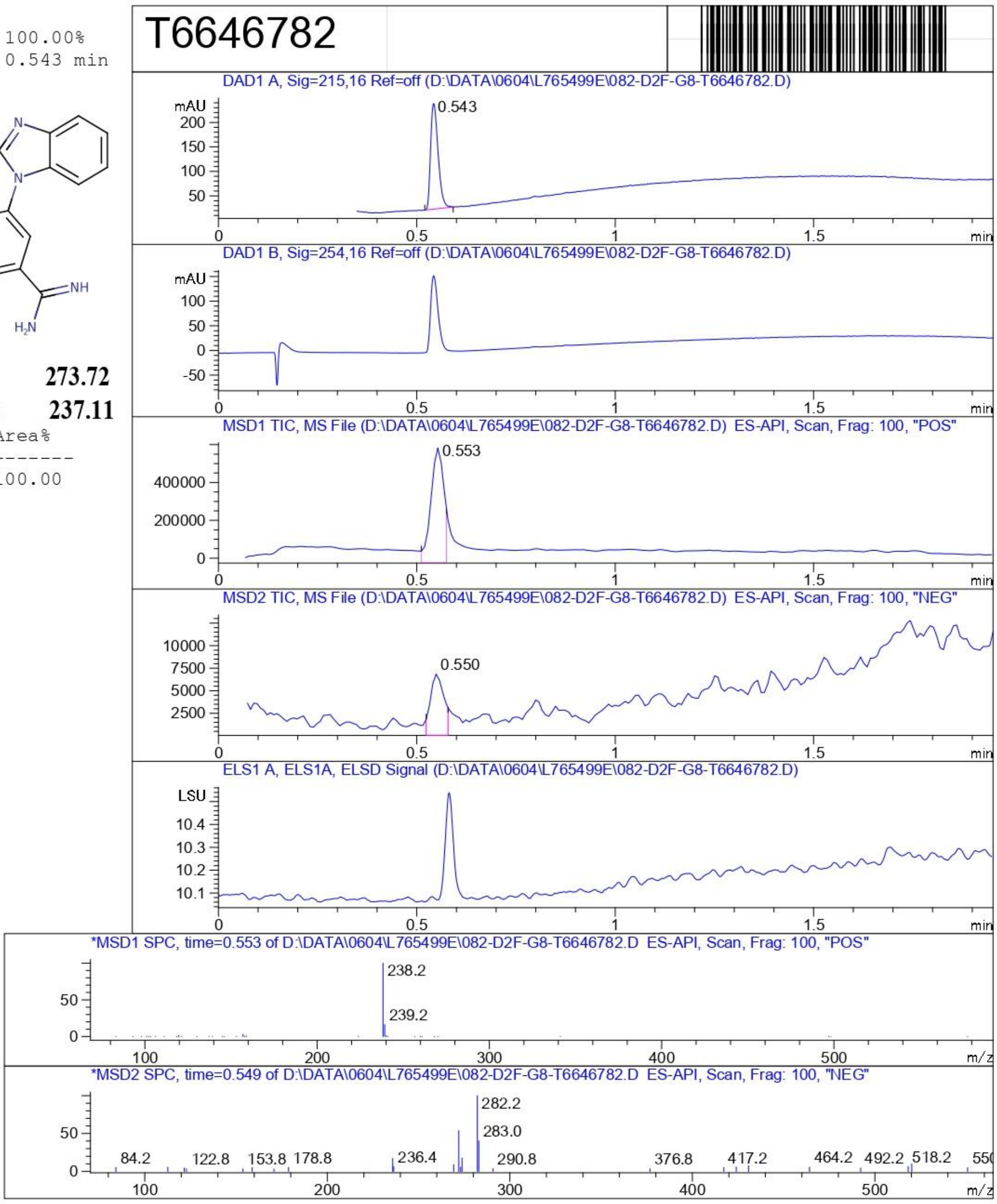

RT 0.553

RT 0.550

HRMS and HPLC spectra of 67

MaxPeak: 100.00%
Ret_Time: 0.517 min

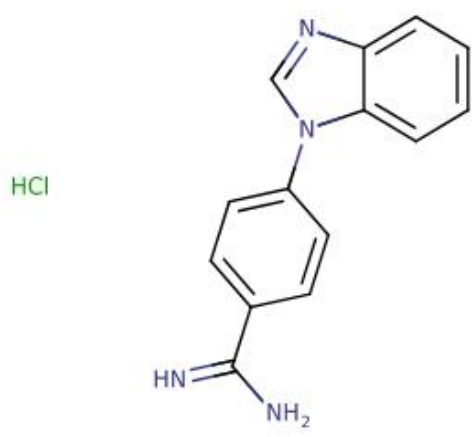

Mol Wt 272.73
Exact Mass 236.12

#	Time	Area%
1	0.517	100.00

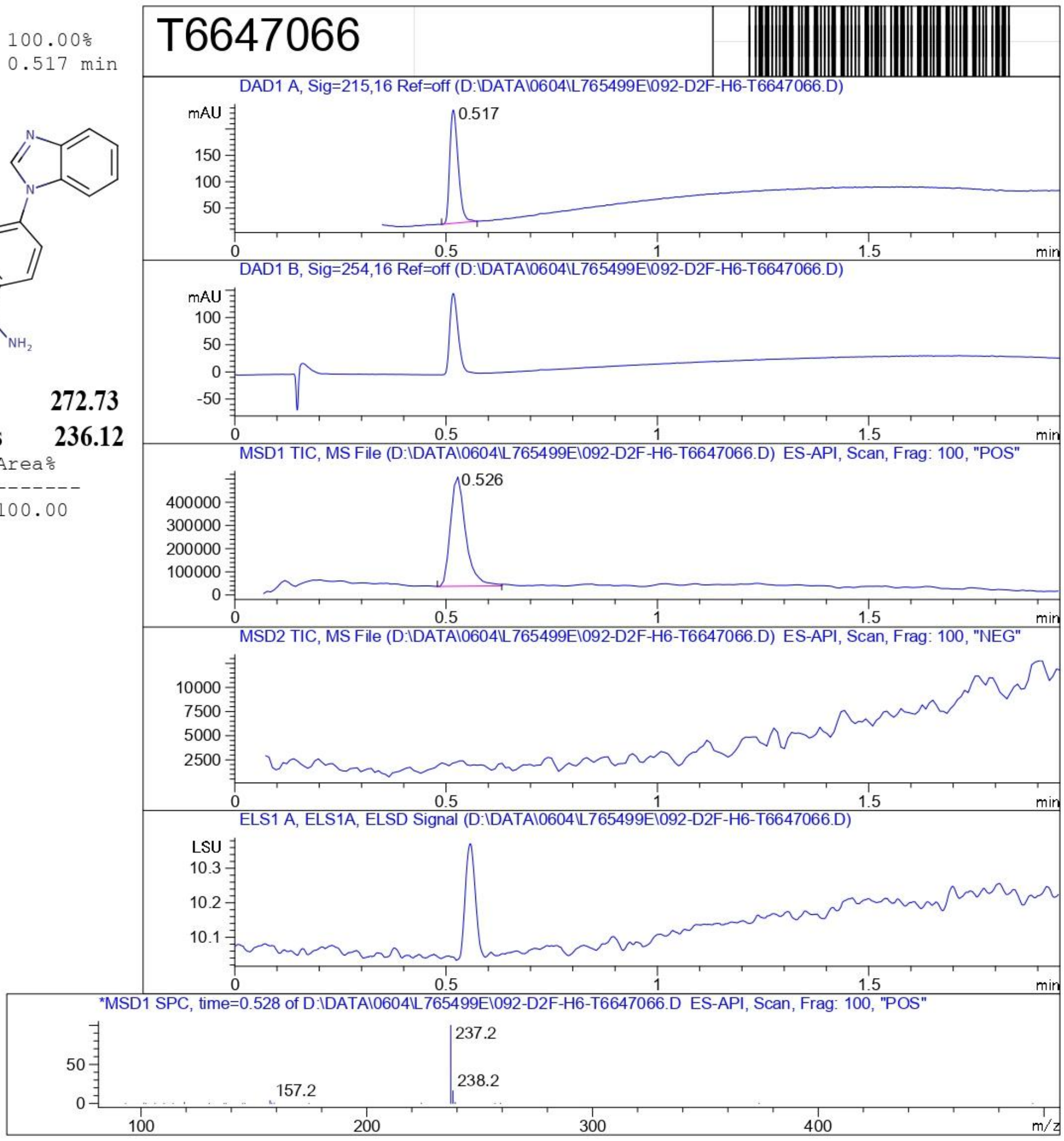

RT 0.526

HRMS and HPLC spectra of 68

MaxPeak: 100.00%
Ret_Time: 0.666 min

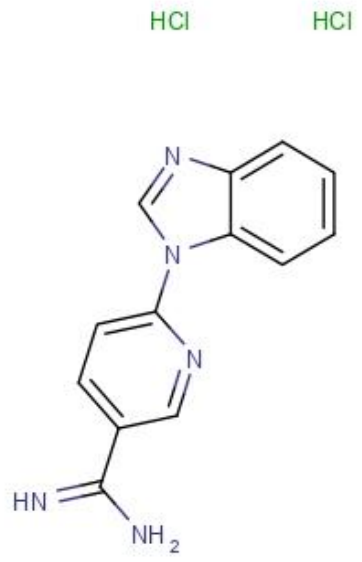

Mol Wt 310.18

Exact Mass 237.11

#	Time	Area%
1	0.666	100.00

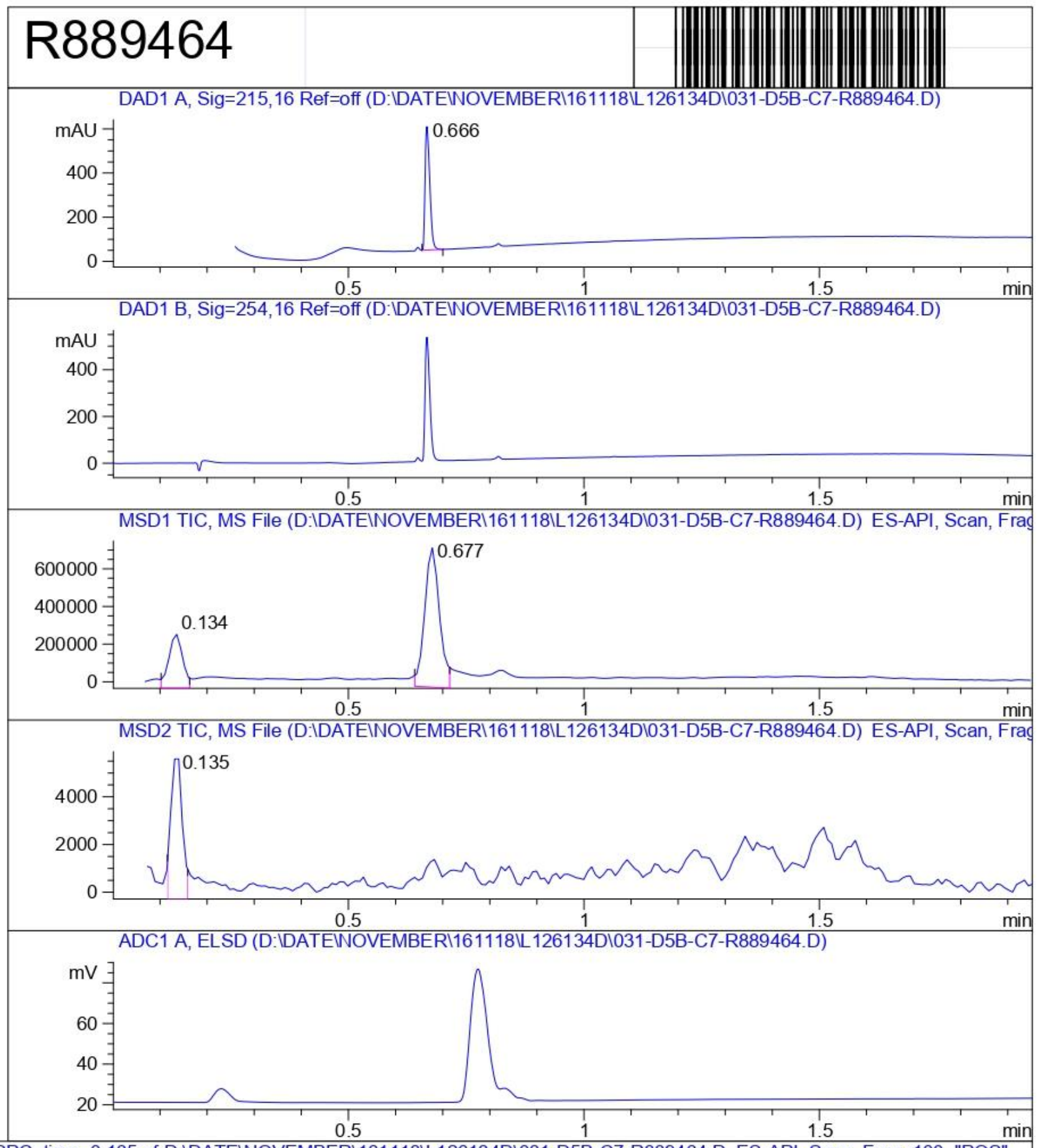

RT 0.134

RT 0.677

RT 0.135

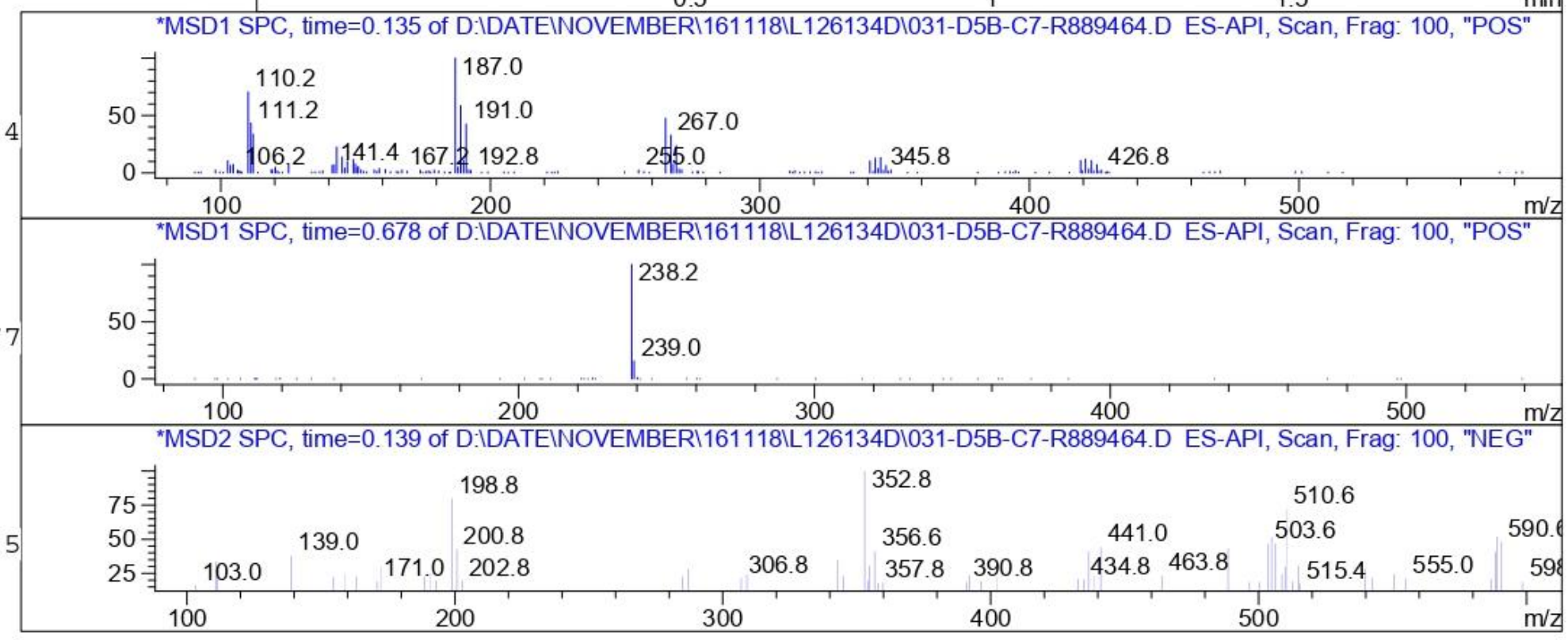

Inj.Date 11/16/2018 T <invalid> -> Acq. Method C:\Chem32\-> ->

HRMS and HPLC spectra of 69

MaxPeak: 98.77%
Ret_Time: 0.850 min

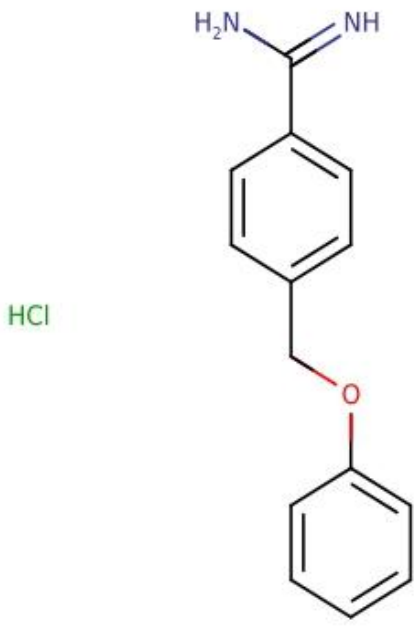

Mol Wt 262.74
Exact Mass 226.13

#	Time	Area%
1	0.850	98.77
2	1.095	1.23

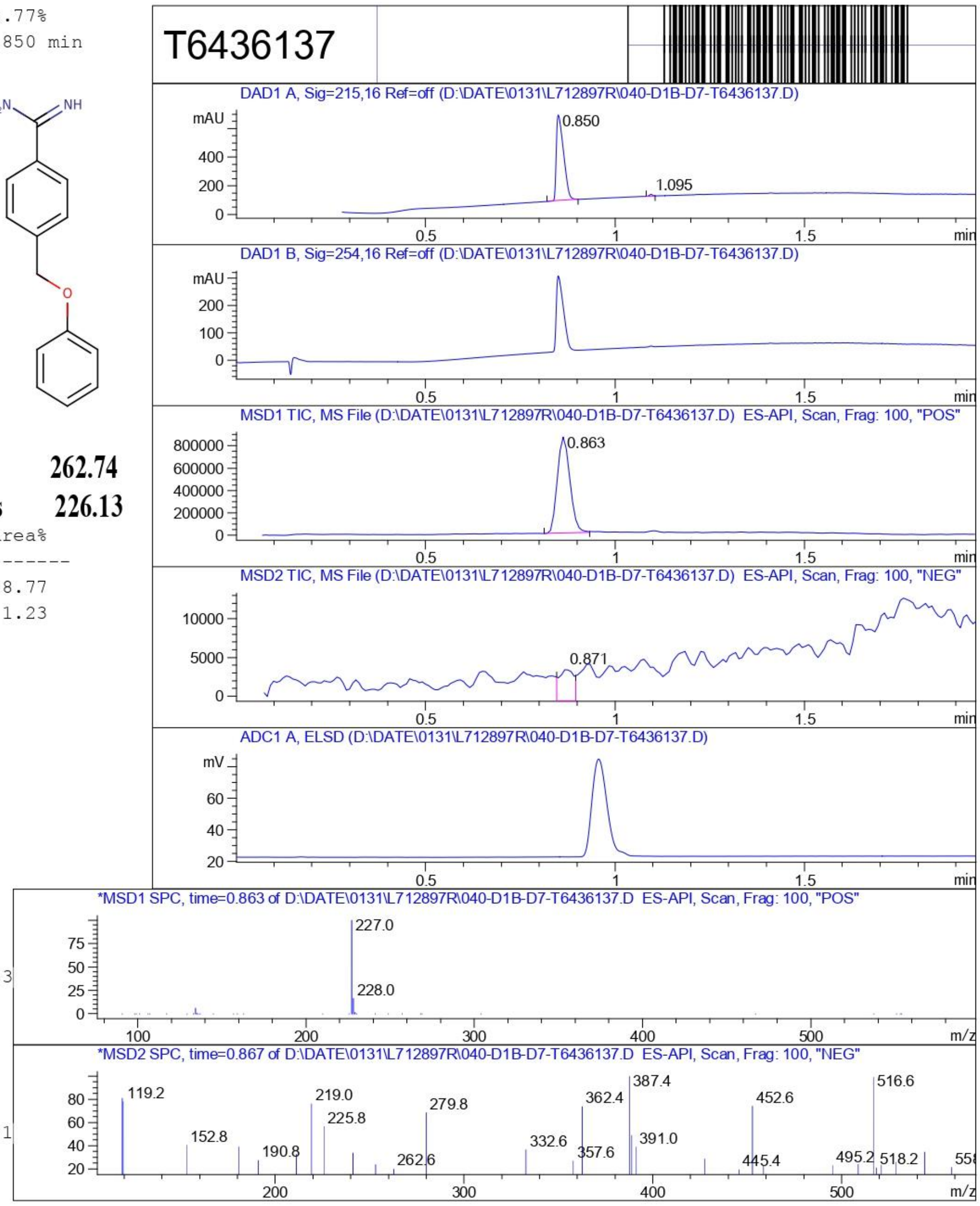

HRMS and HPLC spectra of 70

MaxPeak: 100.00%
Ret_Time: 0.667 min

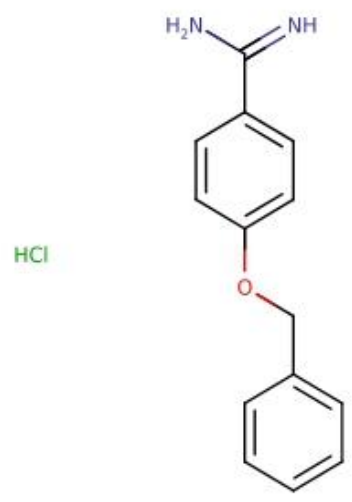

Mol Wt 262.74
Exact Mass 226.13

#	Time	Area%
1	0.667	100.00

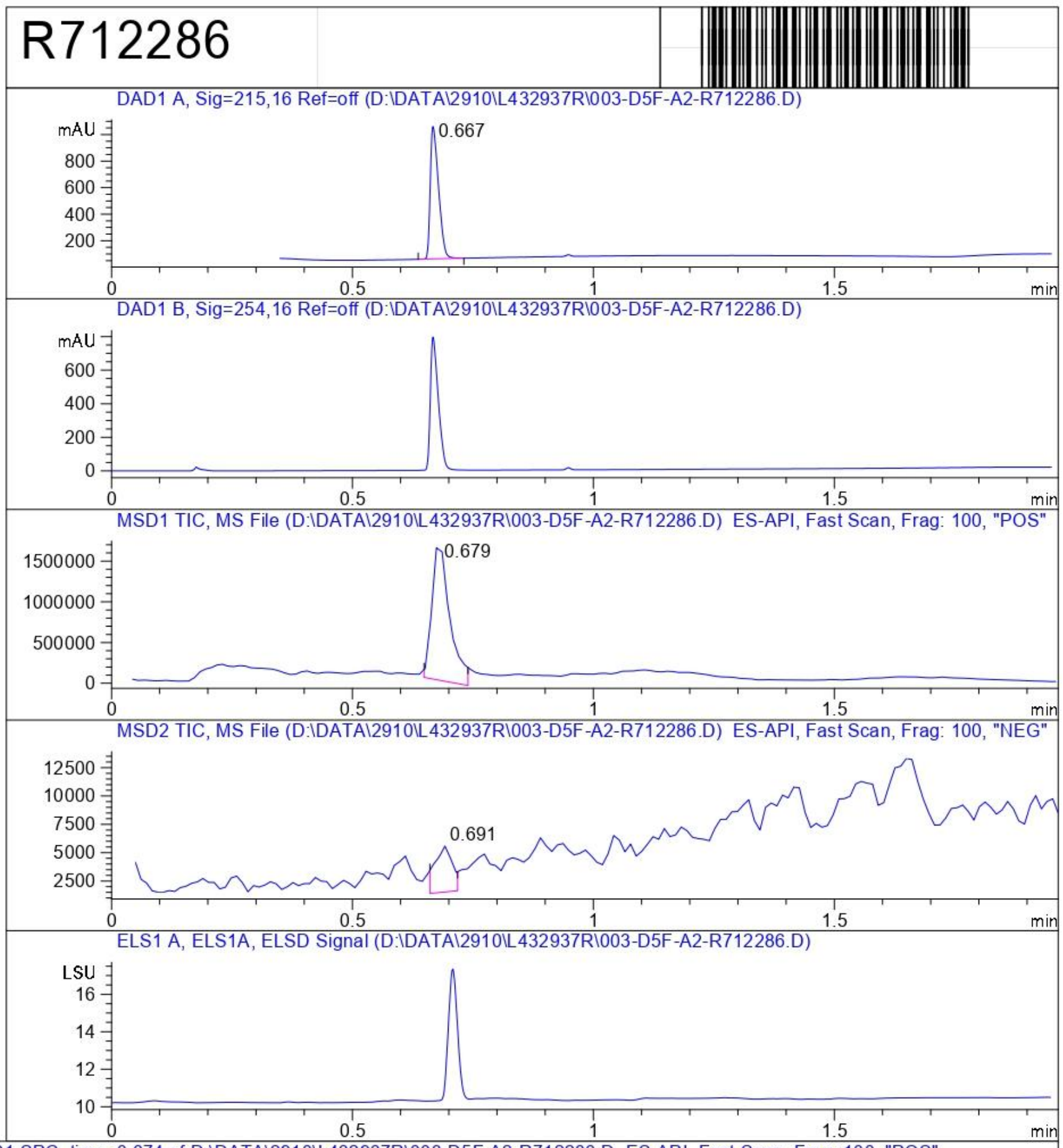

RT 0.679

RT 0.691

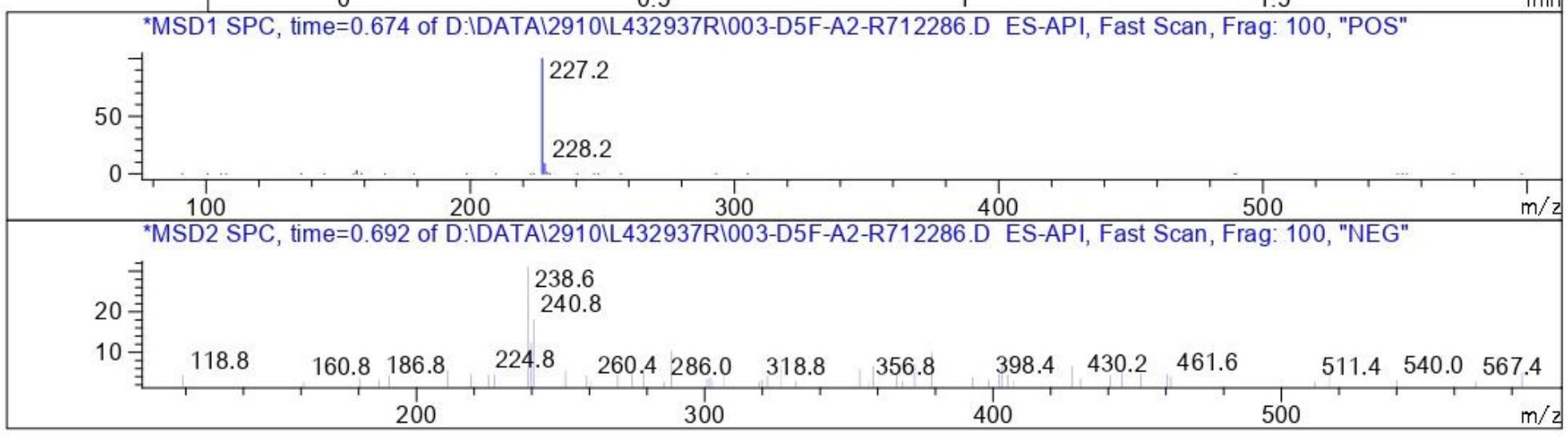

Inj.Date 10/29/2021 H Acq. Method C:\Users\ -> ->

HRMS and HPLC spectra of 71

MaxPeak: 100.00%
Ret_Time: 0.908 min

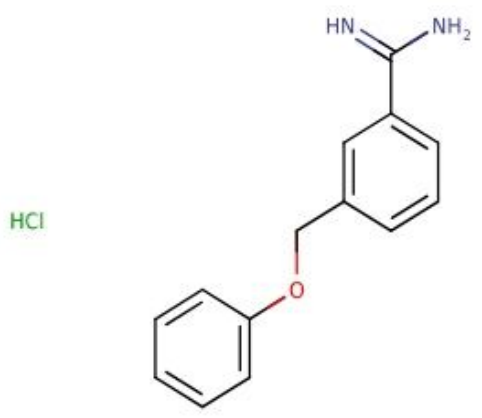

Mol Wt 262.74
Exact Mass 226.13

#	Time	Area%
1	0.908	100.00

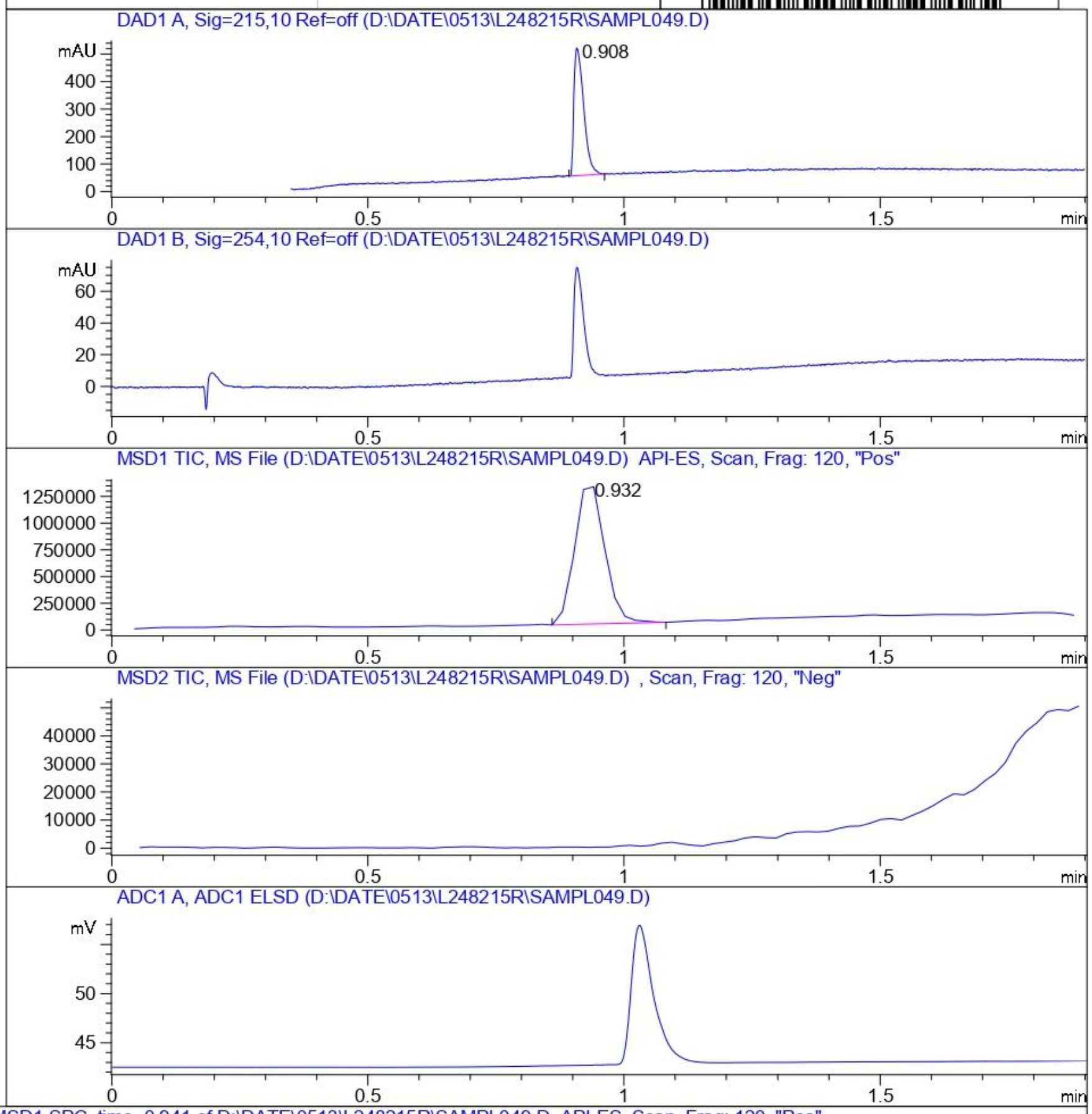

RT 0.932

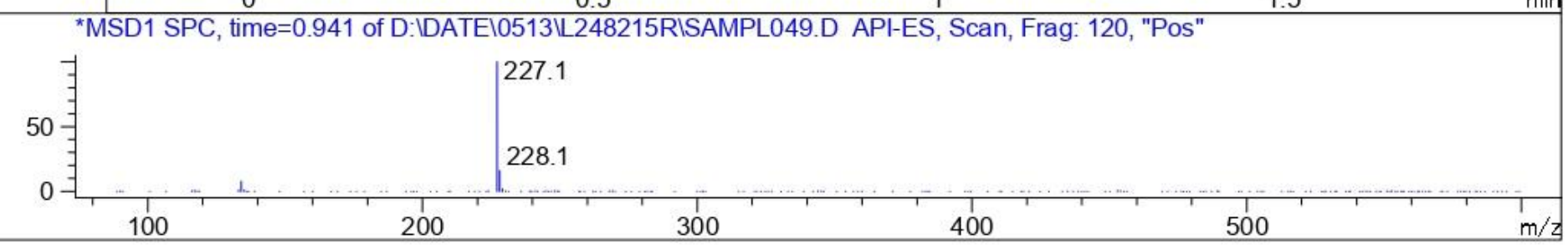

Inj.Date 5/14/2020 O -VL- Acq. Method C:\HPCHEM\-> ->

^{1}H NMR spectrum of 72

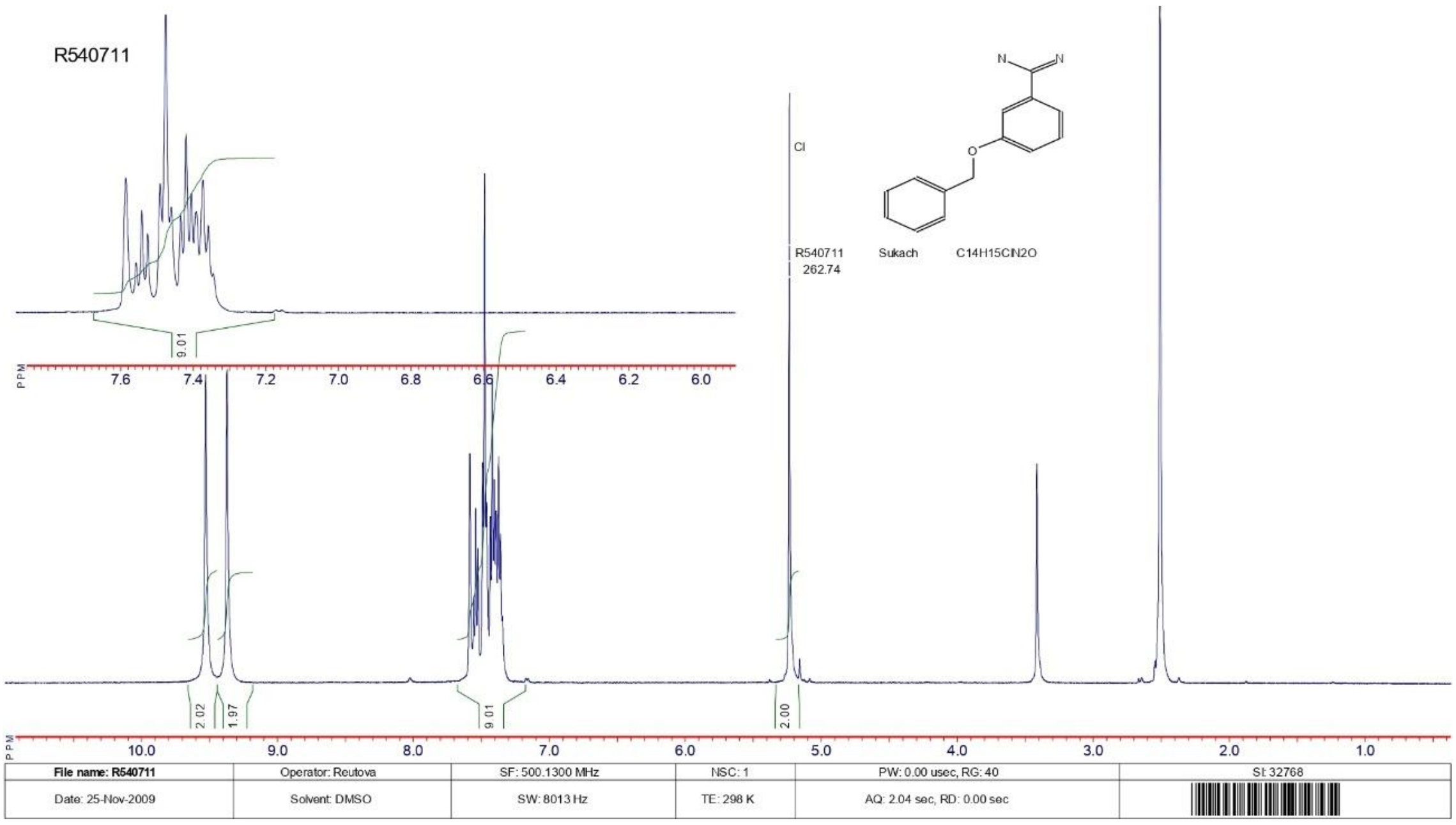

HRMS and HPLC spectra of 73

MaxPeak: 97.89%
Ret_Time: 0.892 min

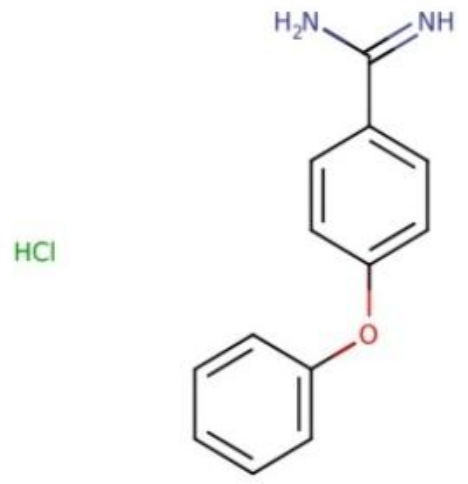

Mol Wt 248.71
Exact Mass 212.11

#	Time	Area%
1	0.892	97.89
2	0.980	1.04
3	1.191	1.07

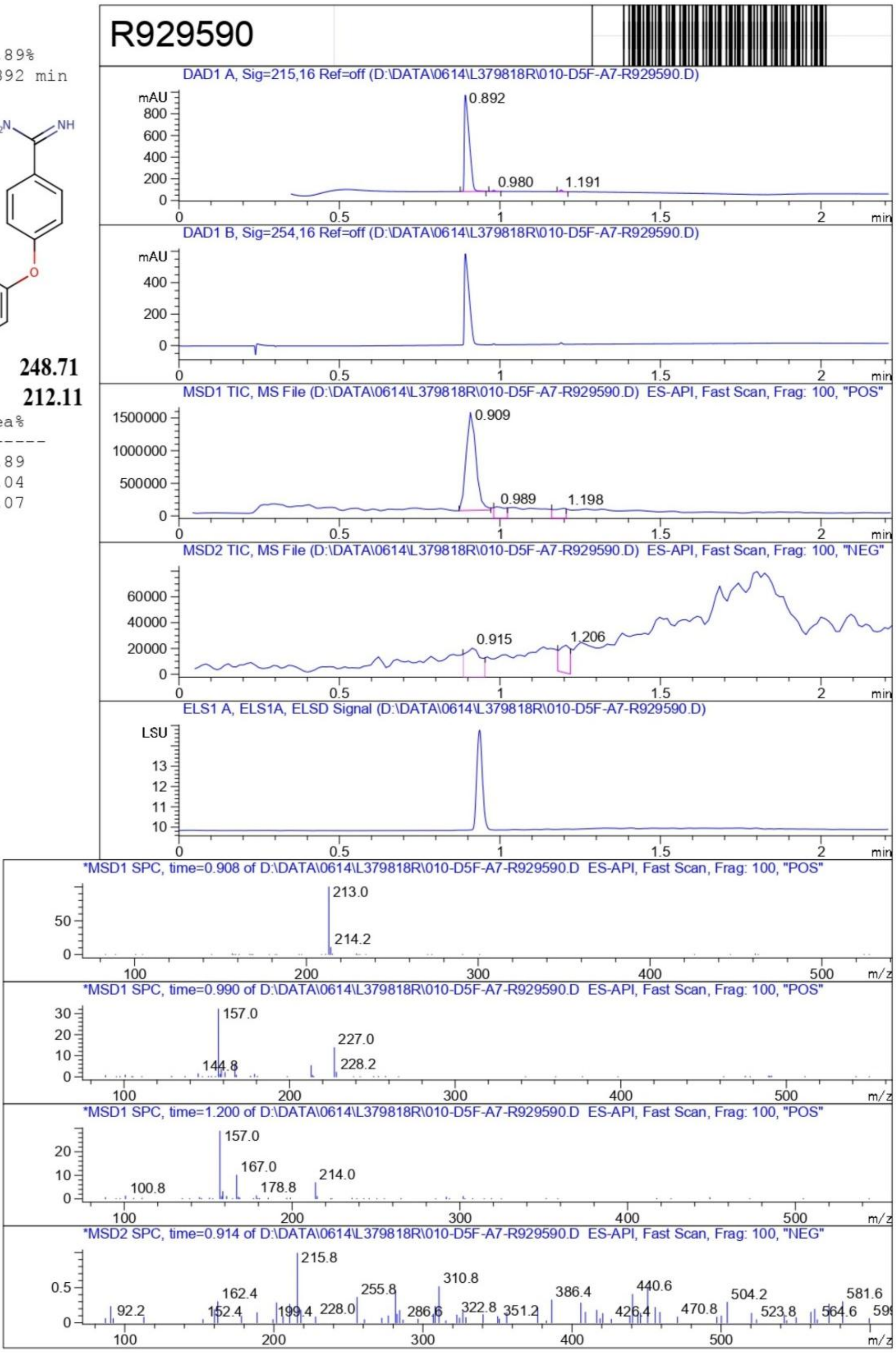

RT 0.909

RT 0.989

RT 1.198

RT 0.915

Inj.Date 6/14/2021 OA Acq. Method C:\Users\ -> ->

RT 1.206

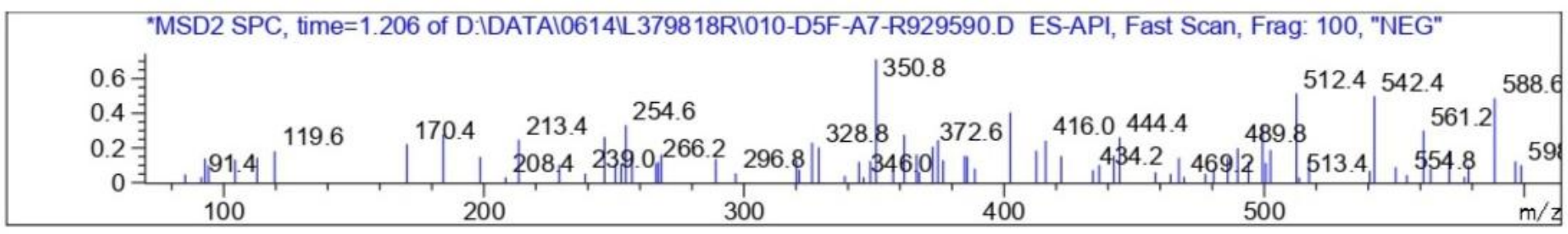

HRMS and HPLC spectra of 74

MaxPeak: 97.96%
Ret_Time: 0.437 min

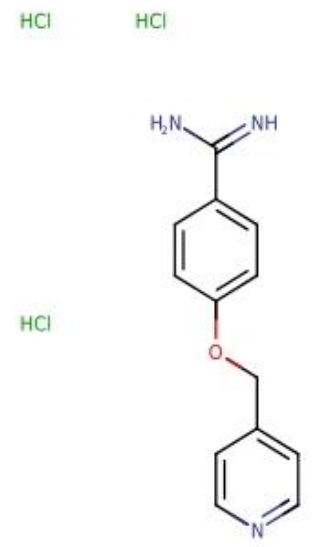

Mol Wt 336.64
Exact Mass 227.12

#	Time	Area%
1	0.437	97.96
2	0.644	2.04

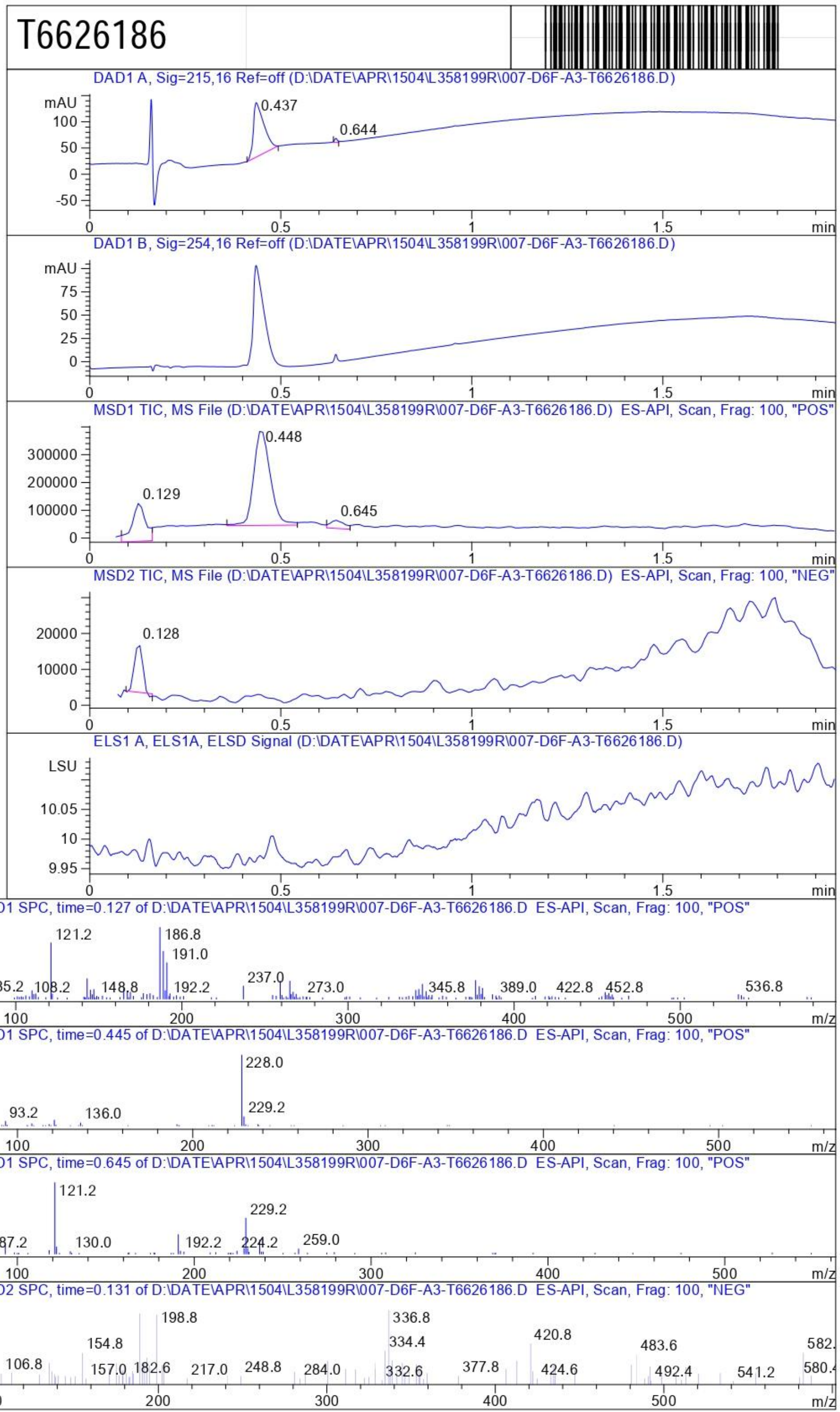

HRMS and HPLC spectra of 75

```
MaxPeak:  95.91%
Ret_Time: 2.380 min
```

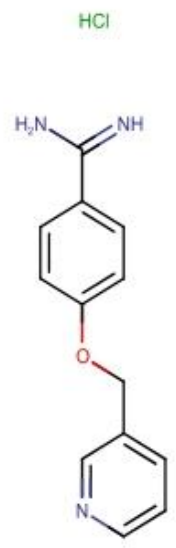

Mol Wt 300.18

Exact Mass 227.12

#	Time	Area%
1	1.975	4.09
2	2.380	95.91

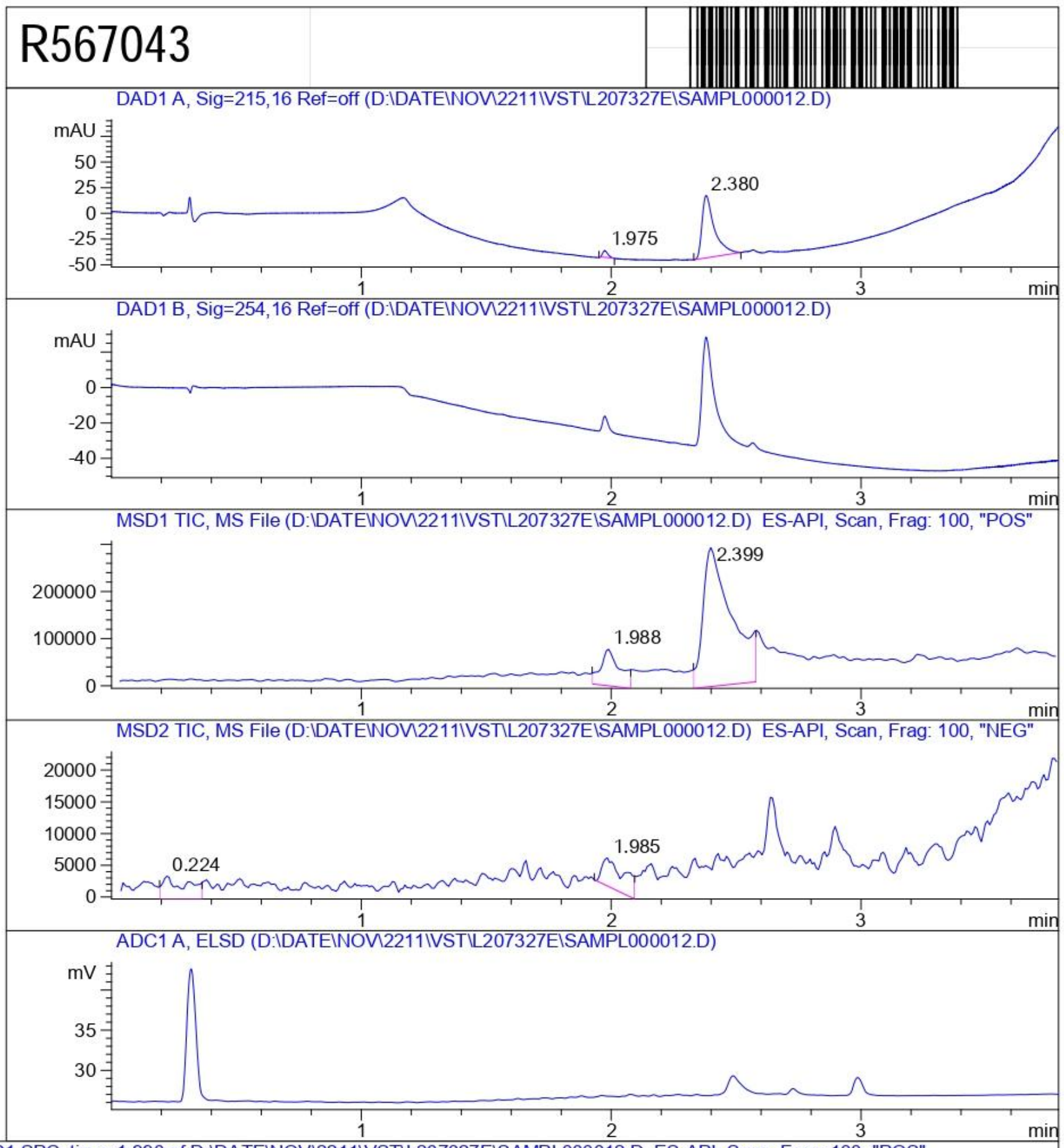

RT 1.988

RT 2.399

RT 0.224

RT 1.985

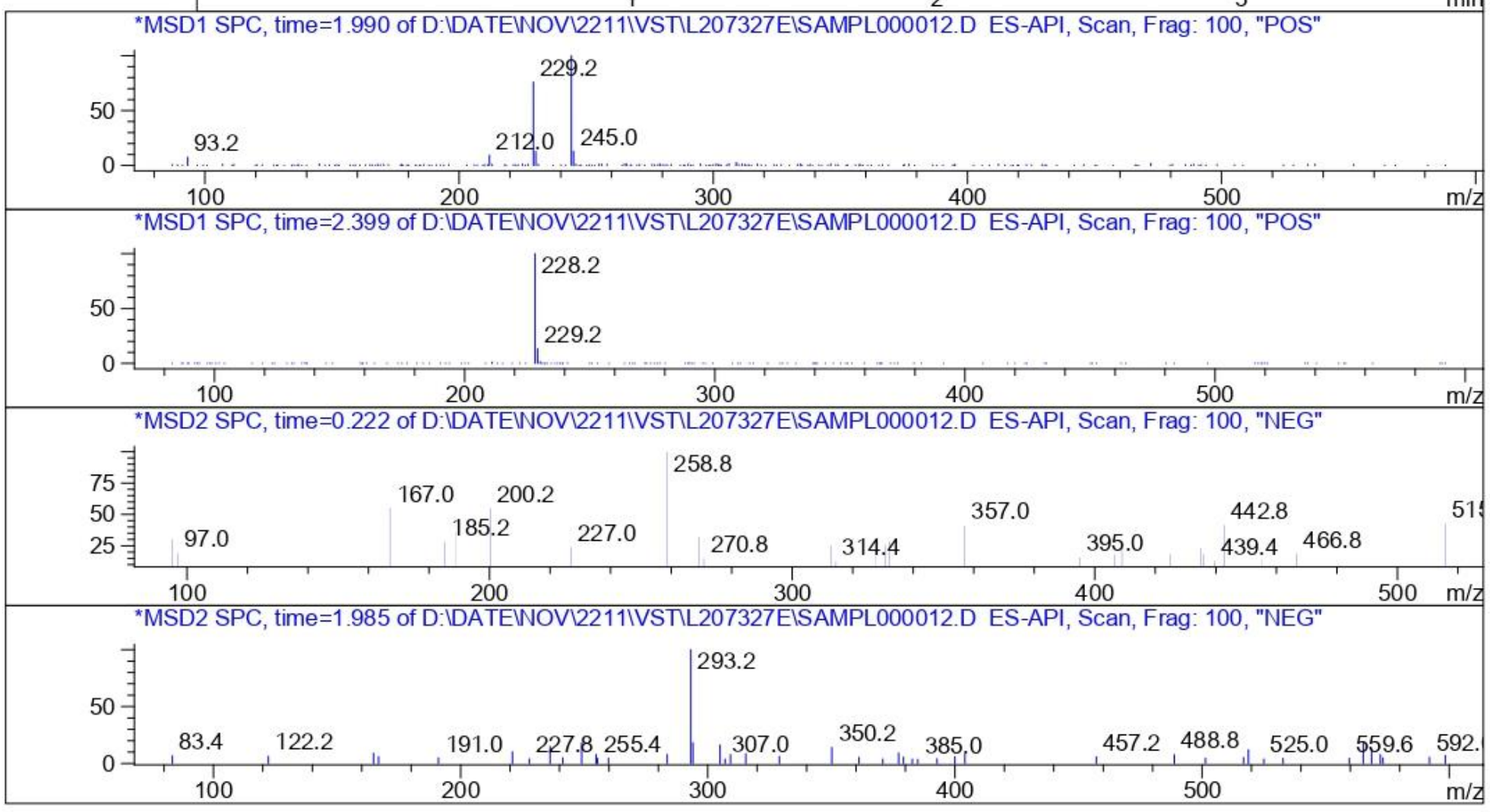


```
Inj.Date 11/22/2019        Y      P1-B-02    -6-      Acq. Method C:\CHEM32\->    ->
```

HRMS and HPLC spectra of 76

```
MaxPeak:  100.00%
Ret_Time: 0.698 min
```

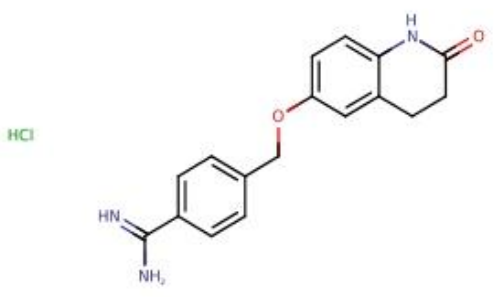

Mol Wt 331.8
Exact Mass 295.15

```
#  Time   Area%
----------------
1  0.698  100.00
```

RT 0.711

RT 0.714

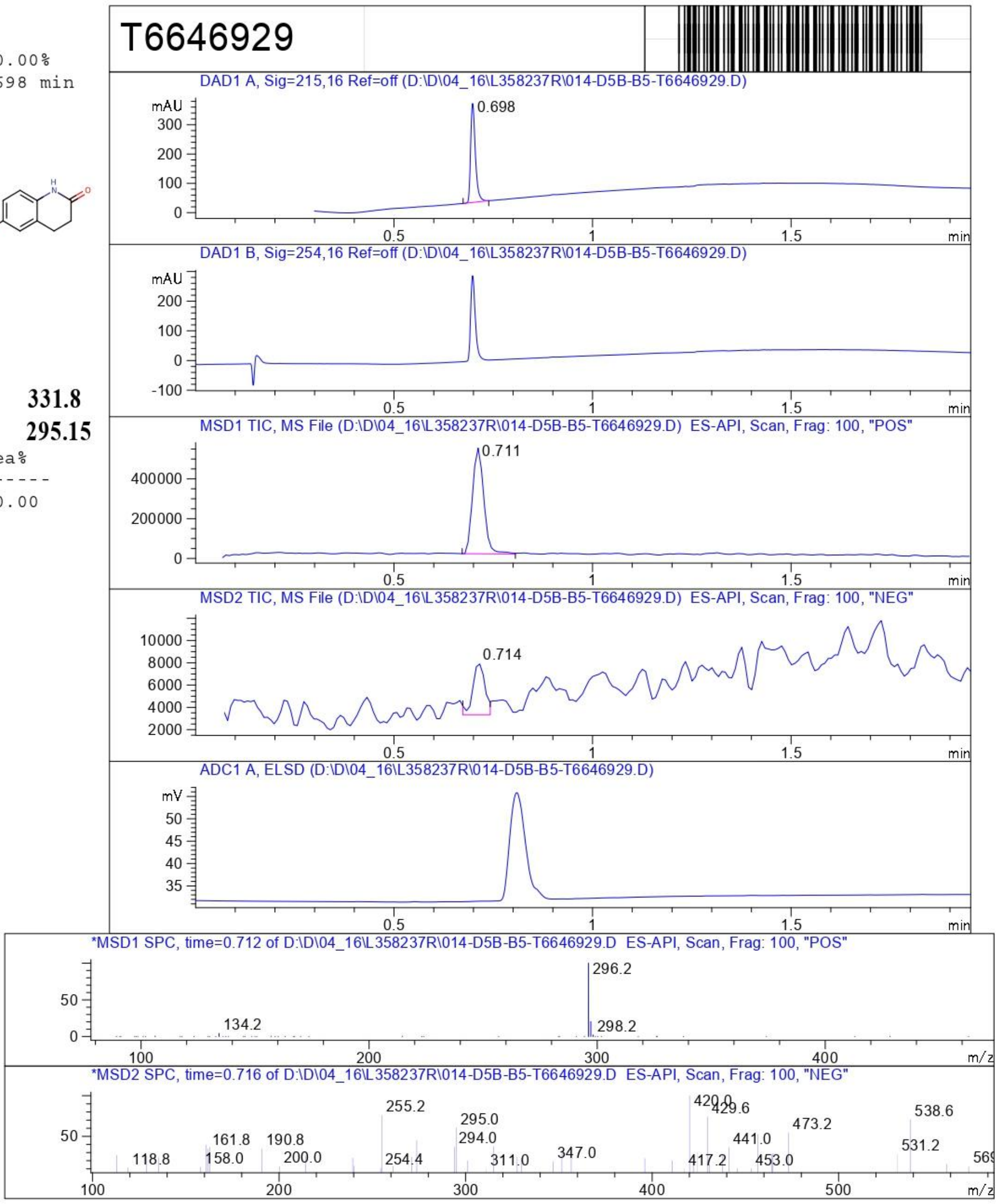

HRMS and HPLC spectra of 77

MaxPeak: 96.79%
Ret_Time: 0.604 min

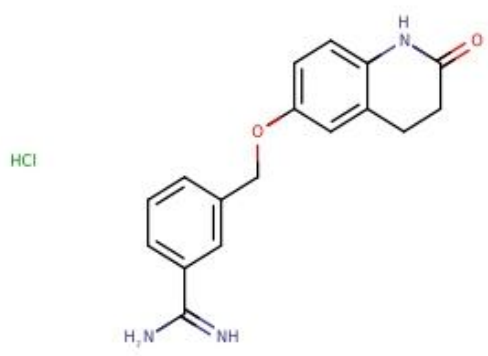

Mol Wt 331.8
Exact Mass 295.15

#	Time	Area%
1	0.604	96.79
2	0.792	3.21

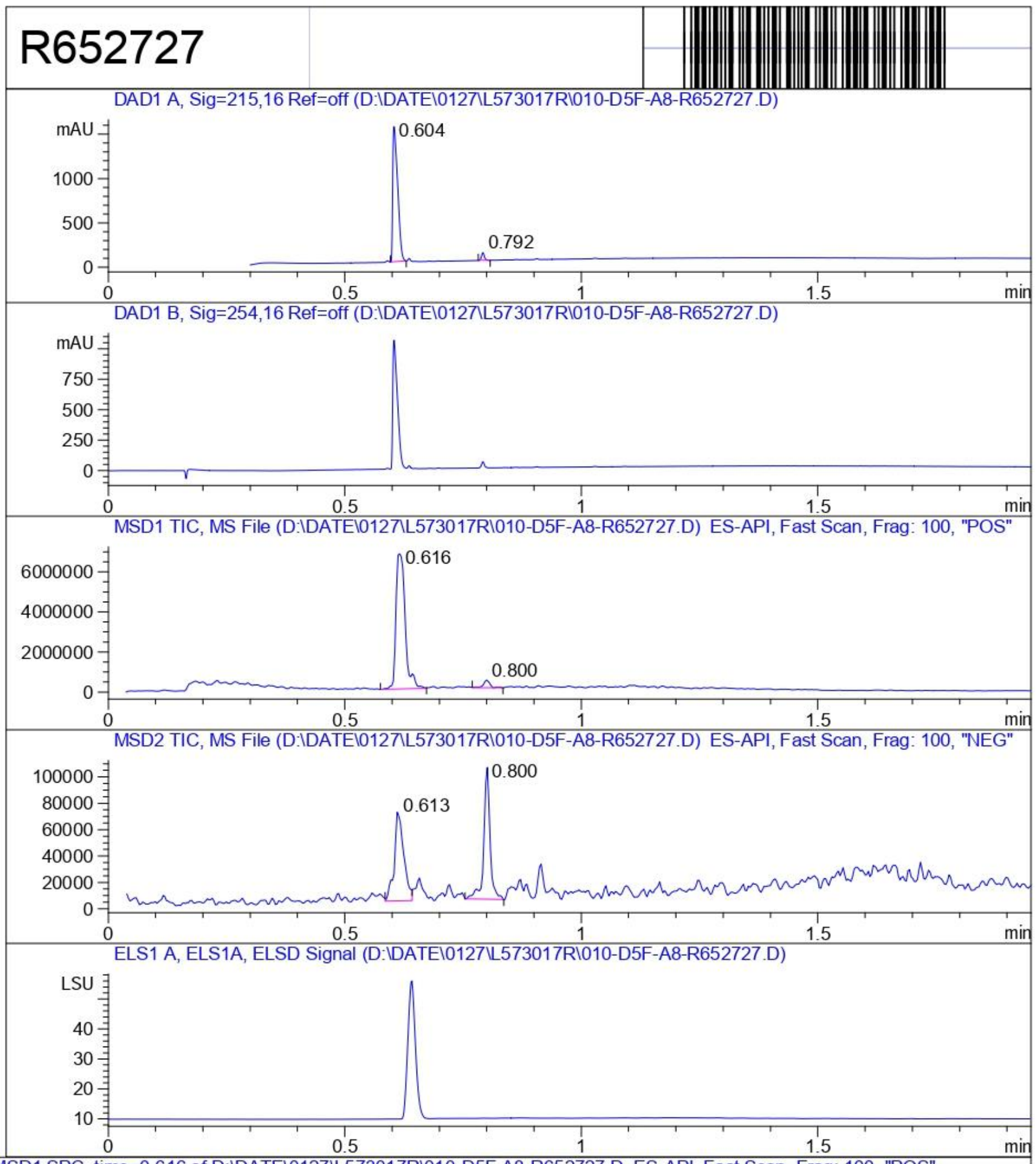

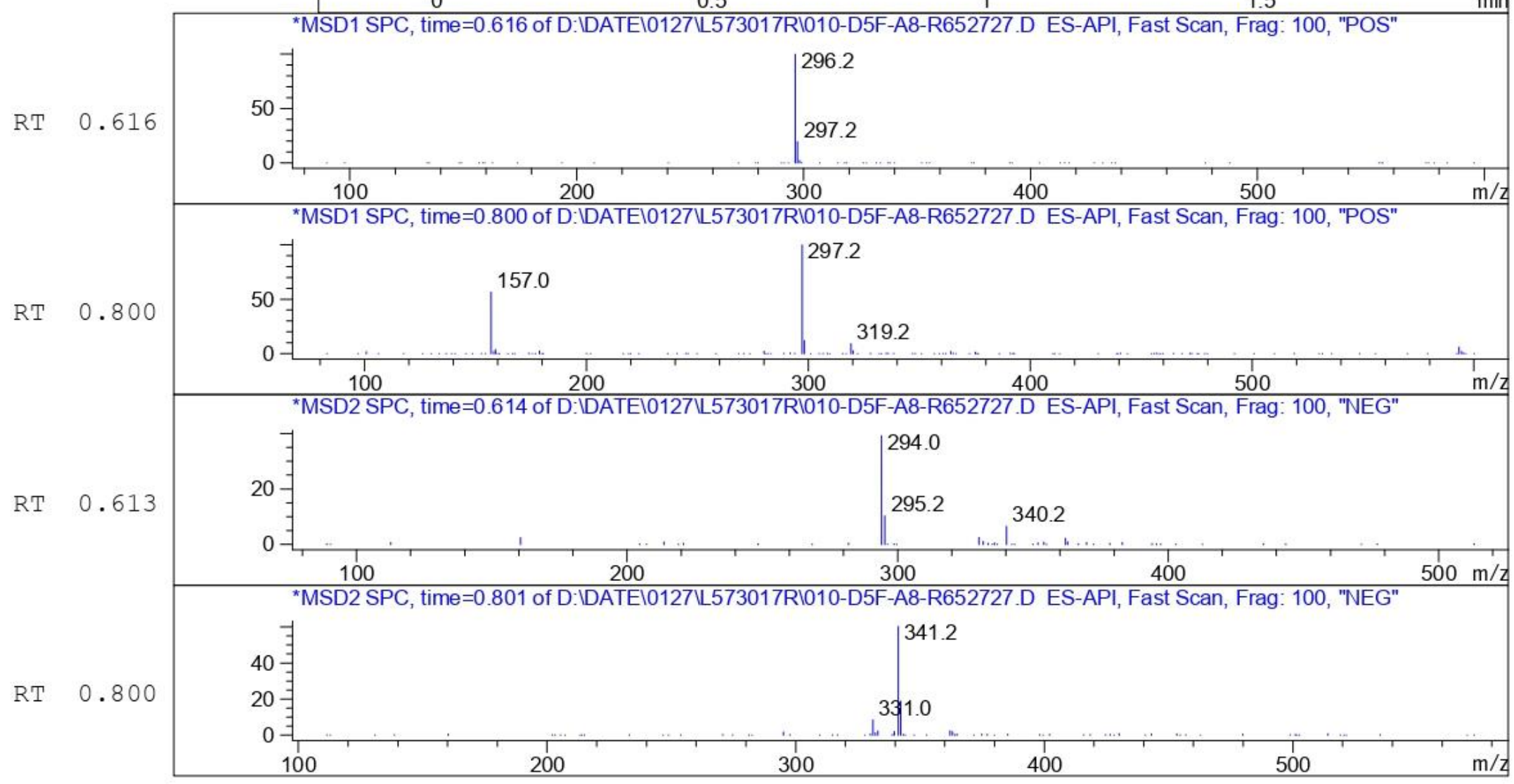

Inj.Date 1/27/2023 E Acq. Method C:\Users\ -> ->
